# Molecular Pathological Diagnosis of Thyroid Tumors Using Spatially Resolved Metabolomics

**DOI:** 10.3390/molecules27041390

**Published:** 2022-02-18

**Authors:** Luojiao Huang, Xinxin Mao, Chenglong Sun, Tiegang Li, Xiaowei Song, Jiangshuo Li, Shanshan Gao, Ruiping Zhang, Jie Chen, Jiuming He, Zeper Abliz

**Affiliations:** 1 State Key Laboratory of Bioactive Substance and Function of Natural Medicines, Institute of Materia Medica, Chinese Academy of Medical Sciences and Peking Union Medical College, Beijing 100050, China; hluojiao@outlook.com (L.H.); sunchl@qlu.edu.cn (C.S.); litiegang@imm.ac.cn (T.L.); soongxw@sina.com (X.S.); lijiangshuo.pumc@gmail.com (J.L.); gaoss5796@outlook.com (S.G.); rpzhang@imm.ac.cn (R.Z.); zeper@imm.ac.cn (Z.A.); 2 Department of Pathology, Peking Union Medical College Hospital, Chinese Academy of Medical Sciences and Peking Union Medical College, Beijing 100730, China; maoxinxin@pumch.cn; 3 NMPA Key Laboratory for Safety Research and Evaluation of Innovative Drug, Institute of Materia Medica, Chinese Academy of Medical Sciences and Peking Union Medical College, Beijing 100050, China; 4 Center for Imaging and Systems Biology, School of Pharmacy, Minzu University of China, Beijing 100081, China

**Keywords:** mass spectrometry imaging, tumor heterogeneity, tumor metabolism, molecular diagnosis model, in situ pathology diagnosis

## Abstract

The pathological diagnosis of benign and malignant follicular thyroid tumors remains a major challenge using the current histopathological technique. To improve diagnosis accuracy, spatially resolved metabolomics analysis based on air flow-assisted desorption electrospray ionization mass spectrometry imaging (AFADESI-MSI) technique was used to establish a molecular diagnostic strategy for discriminating four pathological types of thyroid tumor. Without any specific labels, numerous metabolite features with their spatial distribution information can be acquired by AFADESI-MSI. The underlying metabolic heterogeneity can be visualized in line with the cellular heterogeneity in native tumor tissue. Through micro-regional feature extraction and in situ metabolomics analysis, three sets of metabolic biomarkers for the visual discrimination of benign follicular adenoma and differentiated thyroid carcinomas were discovered. Additionally, the automated prediction of tumor foci was supported by a diagnostic model based on the metabolic profile of 65 thyroid nodules. The model prediction accuracy was 83.3% when a test set of 12 independent samples was used. This diagnostic strategy presents a new way of performing in situ pathological examinations using small molecular biomarkers and provides a model diagnosis for clinically indeterminate thyroid tumor cases.

## 1. Introduction

Thyroid cancer is a common type of endocrine cancer that occurs in the thyroid gland at the throat’s base. Its global incidence has nearly doubled in the last three decades and now accounts for 2% of all cancers [1,2,3]. Thyroid nodules are classified as benign or malignant tumors according to their pathology. Follicular adenoma (FA) is a benign neoplasm with no cancerization. Papillary thyroid carcinomas (PTC) and follicular thyroid carcinomas (FTC) are both well-differentiated carcinomas that represent 90% of all thyroid malignancies [4]. Given that malignant tumors require more surgical intervention than benign tumors, it is critical to perform a timely and precise thyroid tumor diagnosis during surgery. The gold standard for thyroid tumor diagnosis is histopathology, and the differential diagnosis between benign FA, PTC, and FTC highly depends on pathologists’ experiences [5]. However, morphological evaluation alone, based on histological or cytological patterns, is not necessarily accurate for detecting tumor types that have similar characteristics. For example, the only distinction between FA and FTC is the capsular or vascular invasion in FTC. Because of the limited materials and limited section quality, it is difficult to observe clear capsular or vascular infiltration on a frozen tissue section. In addition, the encapsulated follicular variant of papillary thyroid cancer (fvPTC) is frequently misdiagnosed as benign FA [6,7]. Therefore, conventional histopathological diagnosis increasingly calls for the development of a novel pathological diagnosis method.

Metabolic alterations associated with diseased conditions provide important insights into understanding etiological mechanisms and help the discovery of disease biomarkers [8,9]. Metabolomics was thus developed for the characterization of global or targeted metabolite changes in response to pathological changes [10,11], environmental pollutants’ exposure [11], and pharmacological treatment [12], etc. Mass spectrometry imaging (MSI) extends metabolomics by analyzing metabolites in situ on a tissue slice [13,14,15,16]. Without labeling, the relative abundance and spatial distribution of endogenous metabolites can be examined simultaneously. Because of its unique advantage of ionizing metabolites from samples in an ambient environment with minimum pretreatment, the ambient mass spectrometric technique has been rapidly developing in recent years [17,18]. Notably, it has been successfully applied to many disease diagnoses through in situ lipid profiling [19,20,21]. Our group reported an air-flow assisted desorption electrospray ionization (AFADESI)-MSI method under ambient conditions. AFADESI improves in situ collection and sampling of droplets by introducing high-rate air flow into a self-built ion source. As a result, this method can globally map over 1500 endogenous metabolites from biotissue [22]. In addition, using high-throughput capture of metabolite distribution related to tissue histological structure and bio-functions, we successfully discovered alterations in metabolic pathway-related metabolites and metabolic enzymes [23].

In terms of thyroid tumor diagnosis, early studies focused primarily on peptides and lipids as potential in situ biomarkers for distinguishing between PTC and normal tissue, PTC and FA, undifferentiated and differentiated cancer, primary and metastatic tumors, and medullary thyroid carcinoma and carcinoma deteriorated from follicular epithelium [24,25,26,27,28,29]. In a recent study, DESI-MSI was used to diagnose thyroid carcinomas and benign thyroid tissues based on the metabolic profiles obtained from fine-needle aspiration (FNA) biopsy samples. It demonstrated the potential for small molecule metabolites to aid in the diagnosis of thyroid tumors [30]. Thus, in situ profiling of a broader range of endogenous metabolites would provide more chances for diagnosing various pathological types of thyroid tumors, particularly the tough challenge of discriminating malignant FTC from benign FA.

In this study, we propose a molecular pathological diagnosis method for thyroid tumors based on spatially resolved metabolomics analysis using the AFADESI-MSI technique. Tumor region-specific metabolite profiles in high MS resolution were first characterized for each pathological type. Potential diagnostic biomarkers for discriminating between benign follicular adenoma and malignant thyroid cancer were identified by completing multivariate statistical analysis for in situ metabolomics data. Candidate metabolite biomarkers showing high diagnostic accuracy were selected and combined as visual biomarker sets to achieve a rapid diagnostic judgment for FA and PTC (classical and follicular variant of papillary thyroid cancer, cvPTC and fvPTC), FA, and FTC. Next, we built a predictive model analysis based on the metabolite profiles and pattern recognition for further diagnosis validation. Given the difficulty of tumor heterogeneity, the diagnostic model can help determine suspicious foci in some indeterminate cases. Pathologists can position these suspicious foci from MSI images and make further observations in the overlaid H&E stain micrograph during histopathology examination.

## 2. Results

### 2.1. Spatially Resolved Metabolic Profiling

**Figure 1:**
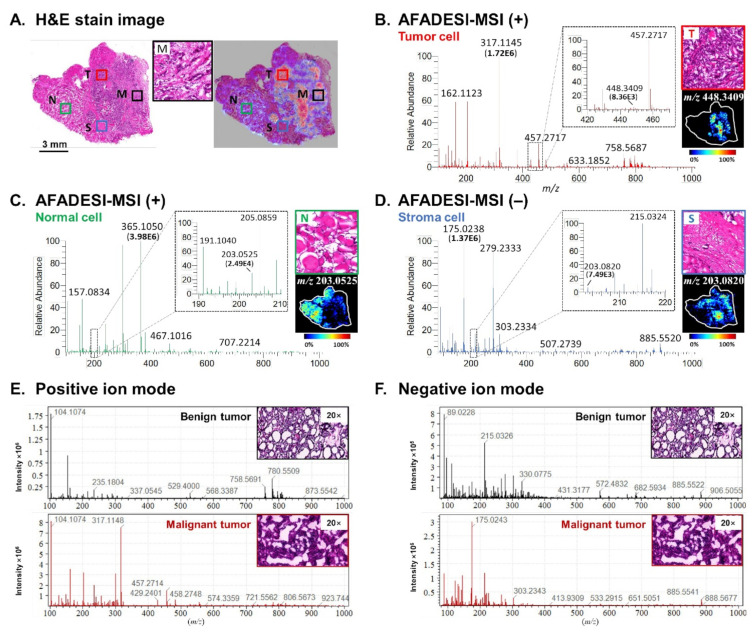
Spatially resolved metabolic profiling of endogenous metabolites in thyroid tumors using AFADESI-MSI. (**A**) H&E stain image of one thyroid tumor section and microscopy-MSI overlaid image. The healthy adjacent thyroid tissue was composed of colloid-filled follicles, which were lined by normal follicular epithelial cells (N, normal follicular epithelial cell). The tumor region consisted of compact small follicles with neoplastic epithelial follicular cells (T, tumor cell). Collagen fibrils in mesh or bundles were seen in the stromal tissue (S, stromal cell). The fibrous stroma had epithelial follicular cells entrapped or attached to the surface (M, mixed stromal cells with tumor cells). (**B**–**D**) Representative mass spectra of different cell types (tumor cells, normal cells, and stromal cells), with an enlarged view of intratumor cytomorphology. (**E**,**F**) Average mass spectra of one benign thyroid tumor and one malignant tumor.

In the thyroid tumor tissue, tumor cells, normal follicular epithelial cells, and stromal cells showed uneven distributions, as seen from H&E stain images (Figure 1A). In part M, stromal cells densely distributed around the tumor cells in a mixed pattern. An image overlay helped locate the microregions for each specific cell type and achieved precise mass spectra extraction. In the average mass spectrum, metabolites below *m*/*z* 500 included amino acids (tryptophan (M–H)^–^ ion: *m*/*z* 203.0820), choline ((M+H)^+^ ion: *m*/*z* 104.1074), carnitine ((M+H)^+^ ion: *m*/*z* 162.1123), fatty acids (arachidonic acid (M–H)^–^ ion: *m*/*z* 303.2334), nucleotides, and peptides, while metabolites above *m*/*z* 500 were mostly lipids, such as phosphatidylcholine (PC(34:2), (M+H)^+^ ion: *m*/*z* 758.5687), phosphatidylinositols (PI(38:4), (M–H)^–^ ion: *m*/*z* 885.5522), lysophospholipids (LysoPC(22:6), (M+H)^+^ ion: *m*/*z* 568.3387), and so on. Apart from the wide range of metabolite information, each cell type also showed a characteristic metabolic profile (Figure 1B–D). The ion intensities of tumor metabolites varied over a broad range from the level of 10^3^ to 10^6^. The good sensitivity of AFADESI-MSI analysis allowed us to detect more metabolites that were in low abundance but still specific for a particular region in the thyroid tumor samples. One benign tumor’s representative metabolic profile showed global differences from that of one malignant tumor (Figure 1E,F). Pairwise statistical classification models were then built for benign and malignant thyroid tumor cells (FA vs. cvPTC vs. fvPTC; FA vs. FTC) and followed by cross-validation (Figures S2–S4, Table S5).

### 2.2. Biomarkers for Discriminating Benign FA from Malignant PTC

A discovery set and a validation set were designed to be analyzed time-independently to explore reliable and reproducible diagnostic biomarkers between benign adenomas and malignant papillary carcinomas. Differential variables discovered from both the discovery set and the validation set were regarded as reproducible variables. The discovery set included 12 FA, 13 cvPTC, and 11 fvPTC, while the validation set included 7 FA, 3 cvPTC, and 8 fvPTC. Both analytical sets gained good classification between FA, cvPTC, and fvPTC in the score plot based on the metabolome of each pathological type (Figures S2 and S3). Finally, 73 reproducible variables between FA and cvPTC, 8 reproducible variables between FA and fvPTC, and 2 reproducible variables between cvPTC and fvPTC were found as potential biomarker candidates (Table S6), and parts of them with identified structures were shown in Table S8.

**Figure 2:**
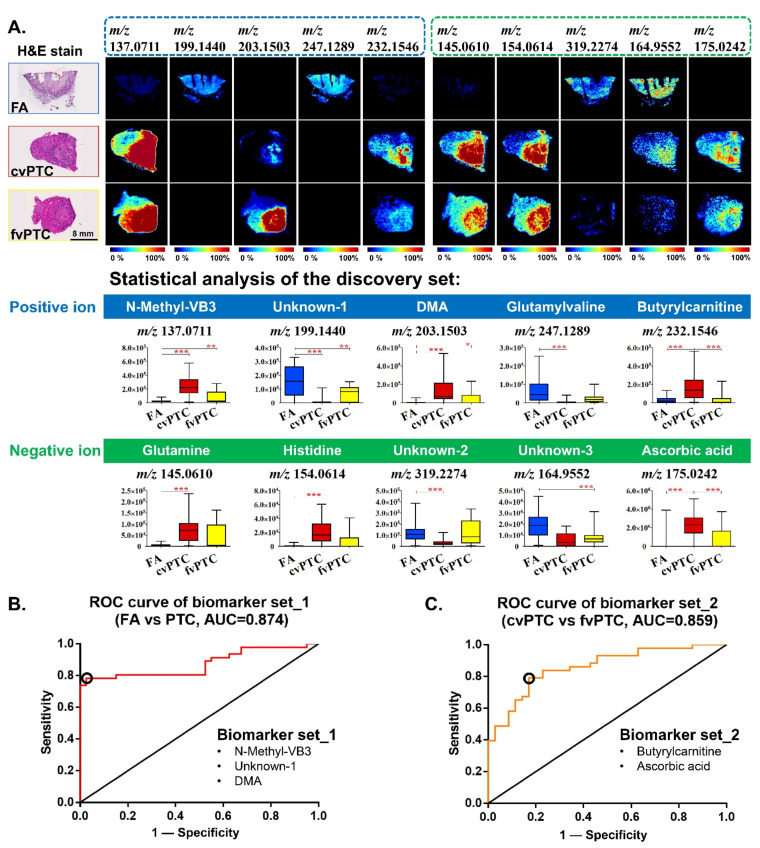
In situ visualization and statistical evaluation of biomarkers for discriminating benign FA from malignant PTC. (**A**) The biomarker panel for differentiation among benign FA, malignant cvPTC, and fvPTC in the discovery set. The upper part is the in situ visualization in three types of thyroid tumor tissues, and the lower part is the statistical analysis of each biomarker in a box plot. (*** *p* < 0.0005, ** *p* < 0.005, * *p* < 0.02) (**B**) ROC analysis of biomarker set_1 between FA and PTC. The diagnostic sensitivity and specificity of the cut-off point were 78.3% and 97.5%, respectively. (**C**) ROC analysis of biomarker set_2 between cvPTC and fvPTC. The diagnostic sensitivity and specificity of the cut-off point were 79.1% and 82.9%, respectively.

The visualization of differential biomarkers helped to rapidly distinguish between benign FA and two malignant PTC subtypes. A diagnostic biomarker panel composed of ten representative differential ions was first determined for fast discrimination (Figure 2A). The ion selection criteria include *p*-value below 0.05, fold change above 2 or below 0.5, good diagnostic efficacy as assessed by receiver operating characteristic (ROC) curve analysis, and a qualitative visual assessment of clear tissue shape. In the positive-ion biomarker panel, three compounds showed their capacity to simultaneously distinguish benign adenoma from malignant papillary carcinoma, which were N-methylnicotinamide (N-methyl-VB3, *m*/*z* 137.0711), unknown-1 (*m*/*z* 199.1440), and dimethylarginine (DMA, *m*/*z* 203.1503). N-methyl-VB3 and DMA were significantly downregulated and barely visible in most FA, while compound unknown-1 showed higher abundance in FA than in malignant tumors. Besides, glutamylvaline (*m*/*z* 247.1289) showed a significant distinction between FA and cvPTC, which was rather low or not even detectable in cvPTC. Butyrylcarnitine (*m*/*z* 232.1546) was an upregulated metabolite specifically for cvPTC compared with FA and fvPTC. Similarly, in the negative-ion biomarker panel (Figure 2A), three compounds were able to simultaneously distinguish FA from cvPTC, which were glutamine (*m*/*z* 145.0602), histidine (*m*/*z* 154.0605), and unknown-2 (*m*/*z* 319.2274). Besides, compound unknown-3 (*m*/*z* 164.9552) was significantly upregulated in FA compared to fvPTC. Ascorbic acid (*m*/*z* 175.0233) showed the highest abundance in cvPTC. The validation set shared consistent results with the discovery set (Figure S5).

To assist in resolving the uncertainty in clinical diagnosis, combined biomarker diagnostic sets were then determined for benign follicular adenoma (FA) and malignant papillary thyroid cancer (cvPTC and fvPTC). The first diagnostic biomarker set consisting of N-methylnicotinamide, unknown-1, and dimethylarginine showed good diagnostic capabilities in distinguishing FA and PTC. The area value under its ROC curve analysis was high at 0.874 (Figure 2B). Next, to determine the subtypes of malignant PTC, the combination of butyrylcarnitine and ascorbic acid was able to differentiate between cvPTC and fvPTC with an AUC value of 0.859 (Figure 2C). The diagnostic efficacy of combined markers was significantly improved compared to that of a single biomarker (Table S10) [31]. The average diagnostic sensitivity was greater than 75%, and the specificity was greater than 80%. Moreover, the performance of the two diagnostic marker sets was repeatable, and the biological errors were minimized with the help of an independent validation sample set.

### 2.3. Biomarkers for Discriminating Benign FA from Malignant FTC

**Figure 3:**
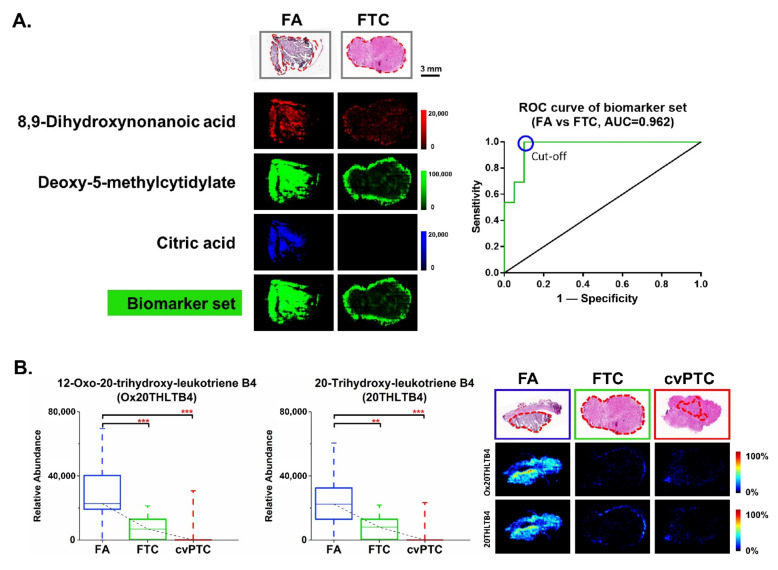
In situ visualization and statistical evaluation of biomarkers for discriminating benign FA from malignant FTC. (**A**) The combined diagnostic biomarker set for benign FA and malignant FTC. Merged visualization was performed by overlapping three ion images (8,9-dihydroxynonanoic acid, citric acid, and deoxy-5-methylcytidylate) via a mixture of different ion channels in the MassImager software (maximum intensity threshold of 100,000). ROC evaluation of this biomarker set between FA and FTC showed the AUC value was 0.962. The diagnostic sensitivity and specificity of the cut-off point were 100% and 90.0%, respectively. (**B**) The comparison of 12-oxo-20-trihydroxy-leukotriene B4 and 20-trihydroxy-leukotriene B4 expression among three thyroid tumor types, FA, FTC, and cvPTC (*** *p* < 0.0005, ** *p* < 0.005). Tumor regions are outlined in red on the H&E slides.

As malignant follicular thyroid cancer is a clinically uncommon case, five FTCs were finally collected and analyzed as a discovery sample set in our study. Good discrimination was observed between FA and FTC in the score plot (Figure S4). The cross-validated predictive ability (Q2) of the model was over 50%, and the model passed 100 random permutation tests, which suggested a good classification capacity without being overfitted. A total of 85 differential ions were rigorously screened as potential biomarker candidates, and finally, the structure of 20 metabolites was identified. (Tables S7 and S9). Nineteen metabolites were significantly downregulated in FTC, while one metabolite, histamine, was upregulated in FTC (Figure S6). Following that, a diagnostic biomarker set was then determined for the clinical diagnosis of FA and FTC using the same selection criteria as previously described. Three metabolites, 8,9-dihydroxynonanoic acid, citric acid, and deoxy-5-methylcytidylate, showing good diagnostic efficacy (Table S10) together with a standard *p*-value < 0.05, fold change > 2 or < 0.5, and a good visual effect, were finally selected. The combined ROC curve analysis showed that this diagnostic biomarker set achieved good diagnostic efficiency (Figure 3A). The AUC value was fairly high, above 0.9, and resulted in 100% diagnostic sensitivity and 90.0% diagnostic specificity.

Interestingly, several eicosanoid metabolites were observed to have significantly higher expression in benign FA than in malignant FTC and PTC. Two molecular ions were identified as 12-oxo-20-trihydroxy-leukotriene B4 (Ox20THLTB4) and 20-trihydroxy-leukotriene B4 (20THLTB4), which were omega-oxides of leukotriene B4 (LTB4) [32,33]. Figure 3B depicts in situ visualization of the two oxidative LTB4 metabolites in FA, FTC, and PTC. They both had very low expression in malignant thyroid tumors and were barely visible in PTC, but they had a relatively high abundance in FA.

### 2.4. Diagnostic Model and Discriminant Analysis

**Figure 4:**
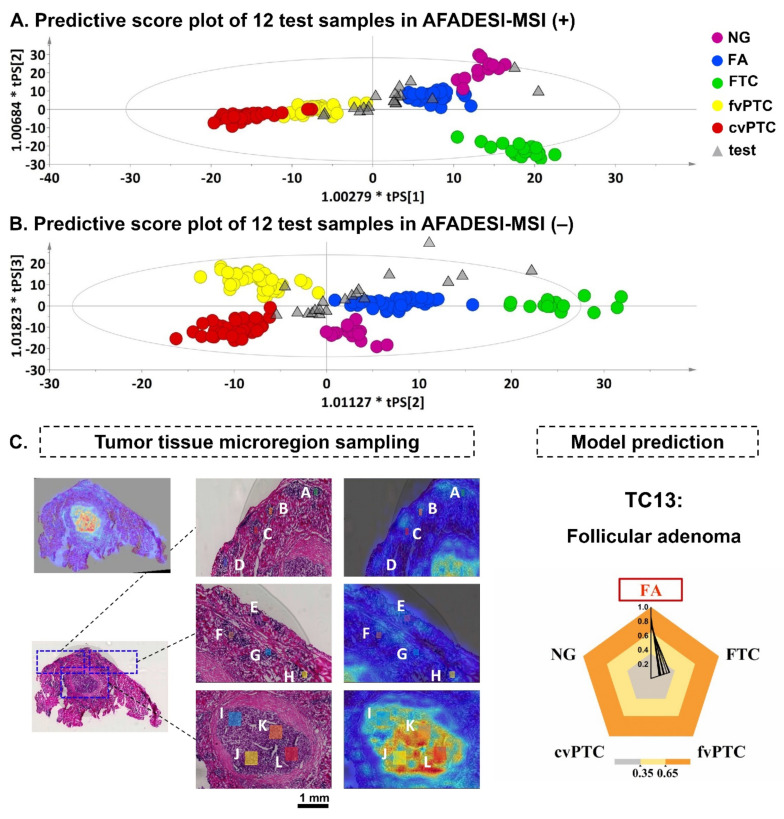
Diagnostic model performance in the test sample set and indeterminate sample cases. (**A**,**B**) Model predictive score plot of 12 test samples based on the mass spectral profile under positive ion mode (**A**) and negative ion mode (**B**). (**C**). Model prediction analysis of indeterminate case TC13. Micro-region with suspicious foci were delineated on an enlarged view of the H&E stain image. Underneath is the MSI image. The integrated result of computational prediction from both positive and negative models is shown on the right. The predicted value is below 0.35: it does not belong to this classification; the predicted value is between 0.35~0.65: it may belong to this classification; the predicted value is above 0.65: it belongs to this classification. Malignancy is the basic output if having both benign and malignant predictions.

This section concentrates on modeling diagnosis based on the metabolic profile and pattern recognized from various thyroid nodules and tumors in order to achieve rapid and automatic evaluations of indeterminate samples. A supervised classification model was constructed based on the metabolic profile extracted from 65 thyroid nodules representing five pathological types, including 5 FTC, 16 cvPTC, 19 fvPTC, and 19 FA from discovery and validation sets and 6 nodular goiters (NG, common benign thyroid lesion). Each type was separated well in the score plot, and there was also a clear distinction between benign nodules, adenomas, and malignant tumors (Figure 4A). Next, a test set consisting of 12 independent samples was examined unbiasedly to assess the predictive ability of this diagnostic model. Several sample points from the same tumor sample were positioned and extracted. By importing each sample point into the model, the 12 test samples intuitively showed their distributions closer to FA and fvPTC types in the predictive score plots (Figure 4A). Meanwhile, the model output evaluated the probability of the possible pathological type for each sample point, which was represented as an average predictive score from both positive and negative models. A predictive score greater than 0.35 was used to determine the final predictive class. The predictive results of 12 test samples were compared with clinical judgments. (Figure S7) There were no errors in identifying malignant types in a total of ten samples. In samples TC4, 5, 8, 9, and 12, benign adenomas were predicted concurrently with malignant tumors. Two samples’ model predictions were inconsistent with the clinical diagnosis. Although sample TC7 was diagnosed as fvPTC, the model output indicated that it belonged to another PTC subtype, cvPTC. The histopathology of sample TC11 showed no nipple-like structure, yet the model predicted it to be cvPTC. TC11 was diagnosed pathologically as a follicular neoplasm. Overall, the diagnostic model established on the metabolic profiles of different thyroid tumor types showed a final predictive accuracy of 83.3%.

The diagnostic model was further used to predict two atypical thyroid tumor cases. For the first indeterminate case of TC13, the pathological type was diagnosed as thyroid adenoma-like hyperplasia by the pathologist, and it was also consistent with an undetermined potential follicular tumor. It is necessary to confirm the possibility of an FTC type. The histopathological examination excluded the possibility of PTC. With the H&E stain image guidance, lesions of dense tumor cells A~L were selected (Figure 4B). The corresponding mass spectral data were imported into the diagnostic submodel consisting of FA and FTC. The model predictive score of suspicious foci in TC13 is shown in Table 1. All sample lesions showed a high predictive score (>0.7) in class FA and a low predictive score (<0.35) in class FTC. This case was finally predicted as FA by the diagnostic model. For the second indeterminate case of TC14, the pathological type was determined as PTC (classical and follicular variation), involving the capsule and surrounding soft tissue. It was also observed that there was a coexistence of benign follicular epithelial cells within the PTC tumor tissue. Likewise, mass spectral data of sample lesions were imported into the submodel without nodular goiter according to histopathological judgment. The model predictive score of all lesions in TC14 is shown in Table S11. More than half of the lesions showed the possibility of PTC, and both cvPTC and fvPTC were identified in the model prediction. Besides, there were two lesion points, B and J, identified as FA. Finally, TC14 was predicted as a papillary thyroid cancer with benign adenoma by the diagnostic model (Figure S8). The predictive result agreed with the actual pathological diagnosis.

## 3. Discussion

Spatially resolved metabolomics analysis based on AFADESI-MSI showed several advantages in investigating diagnostic metabolite features from various thyroid tumor tissues. The advantages of high-throughput and high-sensitivity in metabolic profiling allowed for a broader metabolite coverage without any specific labels. Multiple metabolite classes were detected in thyroid tumor tissue, including amino acids, amines, carnitines, fatty acids, nucleosides, nucleotides, lipids, and peptides. Besides, spatially resolved data extraction reduced the risk of interfering metabolic profiles from nearby cell types and guarantees the most representative metabolic profile from each specific tumor region. All of these advantages contributed to the successful differentiation of benign and malignant thyroid tumors with distinct metabolic compositions and abundances.

The discovery of metabolite biomarkers for discriminating between benign FA and malignant PTC, FA, and malignant FTC also revealed that thyroid cancer undergoes extensive metabolic reprogramming (Tables S6–S9). Several metabolic alterations showed that tumor cells seek to produce sufficient energy and synthetic building blocks for cellular proliferation [34]. Our study found that essential amino acids phenylalanine and histidine and nonessential amino acids serine, asparagine, glutamine, glutamic acid, and arginine showed significant increases in thyroid classic papillary carcinoma. The higher abundance of amino acids in PTC reflected more carbon sources demands as essential nutrients in tumor metabolic remodeling [35,36]. Increased level of phosphatidylethanolamine (PE) class, a major component of the cell membrane, were also observed in PTC, which coincided with the rapid proliferation of cancer cells. Most metabolic changes found in FTC were different from those in PTC, which can be speculated about from their obvious cytological distinction. Nevertheless, some common changes were observed in FTC and PTC compared to FA. Several dipeptides showed low relative abundance in FTC, such as glutamyl-glutamate and glutamyl-valine. The latter was also significantly downregulated in PTC. Glutamyl dipeptides can accept a glutamyl moiety from glutathione degradation via gamma-glutamyltransferase. The varied level of glutamyl dipeptides also indicated an altered glutathione metabolism in follicular adenomas, follicular, and papillary carcinomas [37]. More interestingly, the significantly low expression of several eicosanoids was observed in FTC and PTC compared to benign FA. It is well known that eicosanoids are involved in diverse inflammatory metabolism processes in cells, either anti-inflammatory or pro-inflammatory. Besides, eicosanoids can promote tumor growth by directly activating tumor epithelial cells, normal epithelial cells, or stromal cells [38,39]. It was observed in FA that eicosanoids were distributed from tumor cells to stromal cells and to normal cells. Previously published diagnostic investigations into thyroid tumors using mass spectrometry imaging technique found biomarkers mostly associated with lipids and peptides (Table S12). Our study observed more dysregulations in amino acid metabolism, lipid bio-synthesis, and signaling lipid pathways. This would provide more insights for future study into constructing a comprehensive metabolic reprogramming map and would help precisely identify diagnostic biomarkers, which might be metabolites or enzymes from specific metabolic pathways.

From the discovered metabolic dysregulations in our study, representative differential metabolites were selected and added into three diagnostic biomarker sets for fast in situ visual discrimination between FA and PTC and FA and FTC. Combined biomarker sets proved to significantly improve clinical diagnostic performance [31]. For uncertainty in clinical diagnosis between benign FA and malignant PTC, the first diagnostic biomarker set (N-methyl-VB3, unknown-1, and DMA) was able to identify PTC from FA. Next, to determine the subtypes of malignant PTC, the second diagnostic biomarker set (butyrylcarnitine, ascorbic acid) could identify cvPTC or fvPTC. The performance of the two diagnostic marker sets was repeatable, and the biological errors were minimized with the help of an independent validation sample set (seven FA, three cvPTC, eight fvPTC). To tackle the challenging problem of differentiating between malignant FTC and benign FA, the third diagnostic biomarker set (8,9-dihydroxynonanoic acid, citric acid, and deoxy-5-methylcytidylate) was evaluated with high diagnostic sensitivity and specificity. The performance of the third diagnostic marker set remains to be validated in a larger sample set for further clinical application. Given the significant individual heterogeneity within an uncertain tumor type, clinical discrimination between different thyroid tumor pathological types using only metabolic biomarkers is insufficient. The application of the molecular diagnostic model offers additional benefits in diagnosing indeterminate cases. On the one hand, it showed good diagnostic ability, which was proven by the prediction test on 12 independent samples. On the other hand, model prediction combined with the overlaid H&E stain image helped guide model adjustment by referring to the initial pathological diagnosis. Moreover, for some controversial foci, it was possible to perform an in situ recheck of the histopathological diagnosis after model prediction.

## 4. Materials and Methods

### 4.1. Sample Collection and Preparation

Thyroid tumor tissue samples were collected at Peking Union Medical College Hospital. None of these patients received preoperative treatment. Patient information was under privacy protection. Clinical characteristics of the collected thyroid tumor samples are shown in Table S1. The samples included 22 FA, 5 FTC, 19 cvPTC, 28 fvPTC, 2 follicular neoplasms, and 6 NG. Three samples were diagnosed as possessing two different pathological types. Nodular goiter (NG) is the most commonly encountered benign thyroid lesion in clinics and is quite easy to identify. Except for NG, samples with distinct pathological characteristics were classified into discovery set and validation set for biomarker discovery. NG was additionally added for diagnostic model construction. The remaining samples were used as a test sample set for model prediction. Tumor tissues were immediately snap-frozen in liquid nitrogen after surgical resection and quickly stored at −80 °C until the experiments. For each tissue sample, sections were consecutively cut into 8-μm slices at −20 °C by a cryomicrotome (CM3050S; Leica, Wetzler, Germany) and thaw-mounted onto the microscope glass slides (Superfrost Plus slides, Thermo Fisher Scientific, Waltham, MA, USA). Pathological diagnosis was achieved by H&E staining on one adjacent cryosection. The other sections were stored in a sealed container at −80 °C until analyzed in an ambient environment. Before AFADESI-MSI analysis, tissue sections were dried in a vacuum desiccator from −20 °C to room temperature for 1 h.

### 4.2. AFADESI-MSI Experiment

The AFADESI-MS imaging analysis was performed in both positive and negative ion modes on adjacent tissue sections using a Q-Orbitrap mass spectrometer (Q Exactive, Thermo Fisher Scientific, Waltham, MA, USA) equipped with a home-built AFADESI ion source. The MSI data were acquired in full scan MS mode with *m*/*z* (mass-to-charge ratio) ranging from 100 to 1000. The details of this imaging platform can be found in our previous work [40,41]. The spray voltage was set at (±) 7000 V, and the transport tube voltage was (±) 3000 V. Acetonitrile: water (8:2, *v*/*v*) was optimized as the spray solvent for MSI acquisition. The solvent flow rate was 5 μL/min, and the extraction flow rate was 45 L/min. For each sample analysis, a rectangular positioning frame was drawn on the back side of the slide with the tissue section wholly enclosed in the frame (Figure S1). The entire rectangular region, including the tissue surface and the background part, was continuously scanned on a 2D computer-controlled platform at a horizontal velocity of 200 μm/s in the x direction, followed by a 200-μm vertical step in the y direction. The scanning time for each row was determined by dividing the length by the horizontal velocity, and the total number of data rows was determined by dividing the width by the vertical step. More details about the experimental parameters can be seen in Tables S2 and S3.

### 4.3. Data Processing

Raw data files were converted into .cdf format and imported into the self-developed software MassImager for MSI image reconstruction [42]. An overlaid H&E stain image was also imported to help with the precise delineation of interested cell type regions. The average mass spectral profile was extracted from a specific tumor region associated with different cell types and exported into a txt file as one mass spectral dataset. Multiple regions can be extracted from each tissue sample. All mass spectral datasets were arranged together into one 2D-matrix via MarkerView software 1.2.1 (AB Sciex, Framingham, MA, USA). Observations (sample names) were in columns, and variables (*m*/*z* value) were in rows. Pareto scaling was applied to the 2D-matrix to reduce the relative importance of large values and keep the original data structure [43]. The scaled data matrix was introduced into the SIMCA version 14.0.1 (Umetrics AB, Umea, Sweden) for multivariate statistical analysis. Between FA, cvPTC, fvPTC, and FTC, pairwise orthogonal partial least squares discriminant analysis (OPLS-DA) was performed to explore potential biomarkers. Differential variables were strictly screened step by step based on their respective classification loadings, independent *t*-tests, anomalous change exclusion, and retrospective imaging tests (details on each setup can be found in Supplementary Materials 1.3). Receiver Operating Characteristic (ROC) curve analysis and the Student’s *t*-test analysis were performed using SPSS version 17.0.1 (SPSS Inc., Chicago, IL, USA) and GraphPad Prism version 7.0 (GraphPad software Inc., San Diego, CA, USA). Using the OPLS-DA method, a diagnostic model covering five pathological types was later constructed in SIMCA version 14.0.1. This model included samples from the discovery and validation sets (FA, cvPTC, fvPTC, and FTC) and six NG samples.

### 4.4. Metabolite Identification

Metabolite identification was performed using a liquid chromatography-tandem mass spectrometry (LC-MS/MS) method. Thyroid tissue extracts were pooled together from each tumor type (details on sample preparation can be found in Supplementary Materials 1.4). A Dionex UltiMate3000 HPLC system coupled to a Q-Orbitrap mass spectrometer was used for targeted metabolite analysis (Supplementary Materials 1.5, Table S4) [44]. In the acquired data results (Xcalibur 2.3 software, Thermo Fisher Scientific, Waltham, MA, USA), the exact mass of the molecule and product ions can be matched with literature data and online metabolite databases, such as HMDB, METLIN, and LIPID MAPS. Secondary confirmation was used to match experimental retention time and fragment behavior using readily available standard substances (Supplementary Appendix).

## 5. Conclusions

In summary, our study successfully demonstrated the feasibility of the molecular pathological diagnosis strategy to diagnose thyroid tumors by using spatially resolved metabolomics analysis based on the AFADESI-MSI technique. Differentiated thyroid carcinoma (FTC, cvPTC, and fvPTC) and benign follicular adenoma (FA) can be discriminated visually by three biomarker sets. Additionally, because of the unique advantage of MSI in preserving tissue morphology, the pathological type of the foci of interest in native tumor tissue can be automatically predicted by the diagnostic model. Combining these two approaches results in a new pathological diagnostic solution for thyroid tumor clinical diagnosis based on small molecular biomarkers. Although only a small number of FTC samples were investigated in the discovery sample set, the candidate biomarkers between FA and FTC are still helpful for related studies due to the paucity of research to date. The preliminary findings of dysregulation in several metabolic pathways help understand the pathogenesis mechanisms of thyroid tumors. In future studies, more validation, including a larger sample cohort, can be investigated to corroborate the reliability of the significant biomarkers and the diagnostic model. Moreover, a quality-control system can be implemented for the intraoperative thyroid tumor sample analysis, allowing for higher comparability between samples examined at various measurement periods and an increase in diagnostic accuracy.

## Figures and Tables

**Table 1:** Predictive score results of suspicious focuses in indeterminate case TC13 based on OPLS-DA model.

	Positive Mode		Negative Mode		Computational Prediction	
	Class FA	Class FTC	Class FA	Class FTC	Class FA	Class FTC
TC13-P-1-A	0.9262	0.0738	0.6859	0.3141	0.8060	0.1940
TC13-P-1-B	0.9632	0.0368	0.7837	0.2163	0.8735	0.1265
TC13-P-1-C	0.9195	0.0805	0.8438	0.1562	0.8817	0.1183
TC13-P-1-D	0.8737	0.1263	0.7340	0.2660	0.8038	0.1962
TC13-P-2-E	0.9160	0.0840	0.5535	0.4465	0.7347	0.2653
TC13-P-2-F	0.7397	0.2603	0.7103	0.2897	0.7250	0.2750
TC13-P-2-G	0.9898	0.0102	0.7180	0.2820	0.8539	0.1461
TC13-P-2-H	1.5810	−0.5810	0.6761	0.3239	1.1286	−0.1286
TC13-P-3-I	0.8098	0.1902	0.7215	0.2785	0.7656	0.2344
TC13-P-3-J	0.8937	0.1063	0.8215	0.1785	0.8576	0.1424
TC13-P-3-K	0.9020	0.0980	0.8260	0.1740	0.8640	0.1360
TC13-P-3-L	0.8442	0.1558	0.7925	0.2075	0.8183	0.1817

## Data Availability

The data presented in this study are contained within the article and Supplementary Materials. Raw data are available from the corresponding author on request.

## Supplementary Materials

The following are available online. Figure S1: Illustration of drawing a rectangular box to define the scan area of a tissue section. Figure S2: Model classification result of discovery set. Figure S3: Model classification result of validation set. Figure S4: Model classification result for FA and FTC. Figure S5: The biomarker panel for diagnosis between benign FA, malignant cvPTC, and fvPTC in the validation set. Figure S6: Fold change of differential metabolites in discriminating FA and FTC. Figure S7: Predictive analysis of each test sample based on the diagnostic OPLS-DA model compared with clinical pathological diagnosis. Figure S8: Model prediction analysis of indeterminate case TC14. Table S1: Clinical pathological information of collected thyroid tumors. Table S2: Parameters of AFADESI-MSI platform for in situ analysis. Table S3: Acquisition parameters of Q Exactive mass spectrometer. Table S4: Acquisition parameters of Q-Orbitrap spectrometer in LC-MS/MS. Table S5: Model cumulative R2X, R2Y, and Q2(cum) and Intercepts of R2 and Q2 in one hundred times permutations of each classification model between thyroid tumor subtypes. Table S6: Reproducible differential variables for benign (FA) and malignant thyroid tumor (cvPTC, fvPTC). Table S7: Reproducible differential variables for benign (FA) and malignant thyroid tumor (FTC). Table S8: Identified biomarkers for diagnosis between benign (FA) and malignant thyroid tumor (cvPTC, fvPTC). Table S9: Identified biomarkers for diagnosis between follicular adenoma (FA) and follicular thyroid cancer (FTC). Table S10: Receiver operating characteristic curves (ROC) analysis result of potential diagnostic biomarkers. Table S11: Predictive score results of suspicious focuses in indeterminate case TC14 based on OPLS-DA model. Table S12: A summary of diagnostic studies performed on thyroid tumor using mass spectrometry imaging technique. Figure S9, Figure S10, Figure S11, Figure S12, Figure S13, Figure S14, Figure S15, Figure S16, Figure S17, Figure S18, Figure S19, Figure S20, Figure S21, Figure S22, Figure S23, Figure S24, Figure S25, Figure S26, Figure S27, Figure S28, Figure S29, Figure S30, Figure S31, Figure S32, Figure S33, Figure S34 and Figure S35: Metabolite identification mass spectrum Appendix Figures. References [19,24,25,26,27,28,29,30,45,46] have been cited in the Supplementary Materials.

## Sample Availability

Samples of the metabolite standard substances are available from the authors.
