# Supplementary material for: Molecular Pathological Diagnosis of Thyroid Tumors Using Spatially Resolved Metabolomics
Source: Molecules. 2022 Feb 18;27(4):1390. doi: 10.3390/molecules27041390 (PMC8876246; doi:10.3390/molecules27041390)
Supplement: Supplementary file 1 [file molecules-27-01390-s001.zip › molecules-1548443-supplementary.pdf]

Supplementary Information for

Molecular pathological diagnosis of thyroid tumors using spatially resolved metabolomics

Luojiao Huang[1, ‡], Xinxin Mao[2, ‡], Chenglong Sun[1], Tiegang Li[1], Xiaowei Song[1], Jiangshuo Li[1], Shanshan Gao[1], Ruiping Zhang[1, 4], Jie Chen[2, *], Jiuming He[1, 4, *], and Zeper Abliz[1, 3]

1. State Key Laboratory of Bioactive Substance and Function of Natural Medicines, Institute of Materia Medica, Chinese Academy of Medical Sciences and Peking Union Medical College, Beijing 100050, China.
2. Department of Pathology, Peking Union Medical College Hospital, Chinese Academy of Medical Sciences and Peking Union Medical College, Beijing 100730, China.
3. Center for Imaging and Systems Biology and School of Pharmacy, Minzu University of China, Beijing 100081, China.
4. NMPA Key laboratory for safety research and evaluation of innovative drug, Institute of Materia Medica, Chinese Academy of Medical Sciences and Peking Union Medical College, Beijing 100050, China.

‡ These authors contributed equally to this work.

* Corresponding Authors

To whom correspondence may be addressed. Email: hejiuming@imm.ac.cn.

File ‘Supplementary information_I’ includes:

Supplementary text

Figures S1 to S8

Tables S1 to S12

Appendix

SI References

Contents

1. Experimental details 4

1.1 Thyroid tumor sample information 4

Table S1 Clinical pathological information of collected thyroid tumors 4

1.2 AFADESI-MSI experiment 4

Table S2 Parameters of AFADESI-MSI platform for *in-situ* analysis 4

Table S3 Acquisition parameters of Q Exactive mass spectrometer 4

1.3 Data processing for screening differential variables 5

1.4 Sample pretreatment method for metabolite identification 5

1.5 Analytical method for metabolite identification 5

Table S4 Acquisition parameters of Q-Orbitrap spectrometer in LC-MS/MS 6

2. Results 6

2.1 Figures and Tables 6

Figure S1. Illustration of drawing a rectangular box to define the scan area of a tissue section 6

Figure S2. Model classification result of discovery set 7

Figure S3. Model classification result of validation set 8

Figure S4. Model classification result for FA and FTC 9

Figure S5. The biomarker panel for diagnosis between benign FA, malignant cvPTC and fvPTC in the validation set. 10

Figure S6. Fold change of differential metabolites in discriminating FA and FTC. 11

Figure S7. Predictive analysis of each test sample based on the diagnostic OPLS-DA model compared with clinical pathological diagnosis. 12

Figure S8. Model prediction analysis of indeterminate case ‘TC14’. 13

Table S5 Model cumulative R2X、R2Y and Q2(cum) and Intercepts of R^2 and Q^2 in one hundred times permutations of each classification model between thyroid tumor subtypes 14

Table S6 Reproducible differential variables for benign (FA) and malignant thyroid tumor (cvPTC, fvPTC) 15

Table S7. Reproducible differential variables for benign (FA) and malignant thyroid tumor (FTC) 17

Table S8 Identified biomarkers for diagnosis between benign (FA) and malignant thyroid tumor (cvPTC, fvPTC) 18

Table S9 Identified biomarkers for diagnosis between follicular adenoma (FA) and follicular thyroid cancer (FTC) 20

Table S10 Receiver operating characteristic curves (ROC) analysis result of potential diagnostic biomarkers 22

Table S11 Predictive score results of suspicious focuses in indeterminate case 'TC14' based on OPLS-DA model 23

Table S12 A summary of diagnostic studies performed on thyroid tumor using mass spectrometry imaging technique [1] 24

Supplementary Appendix 26

Metabolite identification 26

(1) Metabolite identification of 8,9-dihydroxynonanoic acid 26

(2) Metabolite identification by standards 27

SI References 52

1. Experimental details

1.1 Thyroid tumor sample information

Table S1 Clinical pathological information of collected thyroid tumors

Pathological Type	Number	M / F	Median Age
FA	22	1:3	47
FTC	5	1:2.2	47
cvPTC	19		
fvPTC	28		
follicular neoplasm	2		
NG	6	1:1.5	39

1.2 AFADESI-MSI experiment

Detailed parameters of the AFADESI-MSI platform are shown in the below tables, including ambient ion source, moving stage and mass analyzer.

Table S2 Parameters of AFADESI-MSI platform for *in-situ* analysis

Parameters	Value
Spray voltage	7000 V（-7000 V）
Tube voltage	3000 V（-3000 V）
Spray gas pressure	80 MPa
Spray angle	60°
Distance from sprayer to surface	0.7 mm
Distance from spray to guide tube	3 mm
Distance from orifice to guide tube	10 mm
Extracting gas	45 L/min
X axis move speed	0.2 mm/s
Y axis step size	0.2 mm

Table S3 Acquisition parameters of Q Exactive mass spectrometer

Parameters	Value
Spray solvent	ACN : H_2O = 8:2 (V/V)
Solvent flow rate	5 μL/min
Scan mode	Full MS
Scan range	100-1000 Da
Maximum inject time	200 ms
AGC target	3e6
Resolution	70000
Scan rate	around 2.0 scans/sec
Sheath gas flow rate	0
Aux gas flow rate	0
Sweep gas flow rate	0
Capillary temperature	350 ℃
Aux gas heater temperature	0

1.3 Data processing for screening differential variables

(1) The value of variable importance (VIP) was set above 1, which reflected the influence of each variable on classification and did Jack-knifed confidence interval analysis.

(2) The significance of each differential variable was assessed using an independent t-test (SPSS Statistics 17.0.1, USA). Statistically differential variables were determined with a p-value less than 0.05.

(3) Actual variations between groups were observed by creating a line plot for each potential biomarker in SIMCA, which ruled out anomalous changes caused by only one sample within one group.

(4) Retrospective imaging tests in MassImager were used to further validate whether their ion images showed relevant contours to the shape of tissue sections.

1.4 Sample pretreatment method for metabolite identification

(1) Thyroid tumor tissues (FA, FTC, cvPTC, fvPTC) were weighed at 100 mg, cut into small pieces, and placed in a 2 mL homogenization tube. 400 uL 4℃ water and three ceramic beads (3.0 mm in diameter) were added into the tube.

(2) Homogenization tubes were fixed in a tissue homogenizer (MasterPrepTM-24). The homogenization parameters were set as follows: frequency 30 s/time, speed 4.0 m/s, interval time 30 s, repeat 4 times.

(3) 100 uL of homogenate was pipetted into a clean 1.5mL EP tube, followed by 400 L of acetonitrile.To precipitate protein, samples were mixed for 2 minutes before being centrifuged at 10,000 rpm for 5 minutes at 4°C.

(4) Supernatant was transferred into a new clean 1.5 mL EP tube and dried for about 2 h using a speed vacuum concentrator (Savant SPD121P-230 SpeedVac, Thermo Fisher Scientific, USA). Extracts were redissolved in 100 uL solvent of ACN/0.1%FA·H2O (2:98). 50 uL of each type of tumor extract was pipetted to make the final pooled extract.

1.5 Analytical method for metabolite identification

Metabolites were separated on a Waters UPLC HSS T3 column (1.8 μm, 2.1×100 mm) at room temperature. Aqueous 0.1% formic acid and acetonitrile were selected as mobile phase with linear gradient elution at the flow velocity of 0.25 mL/min. The linear gradient elution procedure is as follows: Equilibrium for 8 minutes (2% acetonitrile), 0-9 minutes (2% acetonitrile to 60% acetonitrile), 9-18 minutes (60% acetonitrile), 18-20 minutes (60% acetonitrile to 100% acetonitrile), 20-27 minutes (100% acetonitrile). Full MS and PRM scan mode in both positive and negative ion modes were used in mass spectrometric analysis.

Table S4 Acquisition parameters of Q-Orbitrap spectrometer in LC-MS/MS

Parameters	Value
Spray voltage	3500 V （-3500 V）
Capillary temp	350 ℃
Sheath gas flow rate	40 psi
Aux gas heater temp	220 ℃
S-Lens RF level	55
Solvent flow rate	0.25 mL/min
Scan mode	Full MS, PRM
Scan range	70-1000 Da
Maximum inject time	100 ms
AGC target	3e6, 1e6
Resolution	35000, 17500
N(CE)/ stepped N(CE)	nce: 25, 30, 35

2. Results

2.1 Figures and Tables

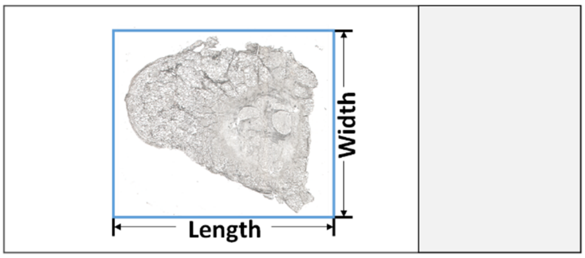

Figure S1. Illustration of drawing a rectangular box to define the scan area of a tissue section

The sample shown in the figure was measured with a length of 19 mm, width of 17 mm, The calculated scanning time for each row was 95 s, and the total row count was 85. The total analytical time for this sample was 2.24 hours.

Pairwise statistical classification modeling of three thyroid tumor types: FA, cvPTC and fvPTC

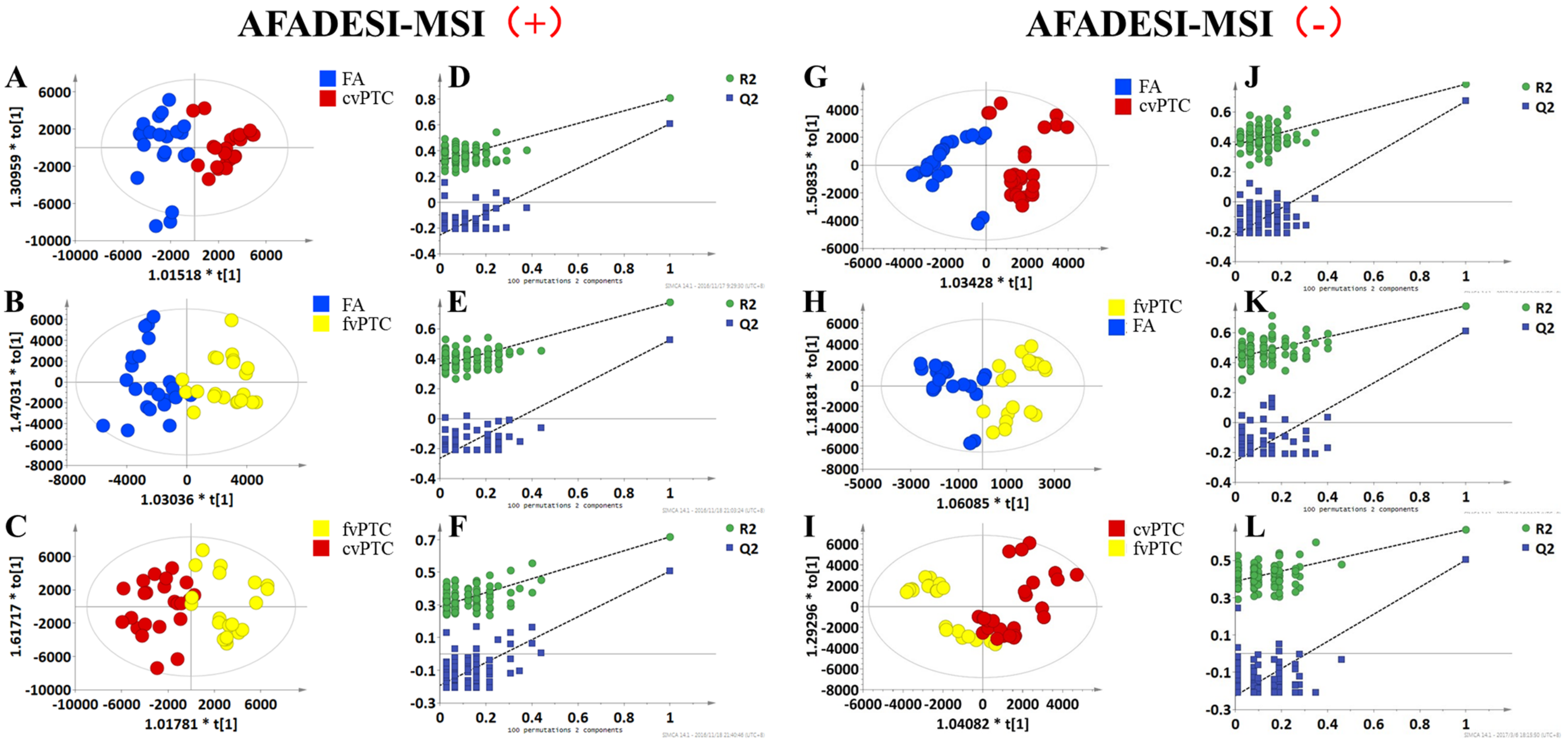

Figure S2. Model classification result of discovery set

(A) (B) (C) score plot of OPLS-DA analysis between FA, cvPTC, fvPTC in positive scan mode; (G) (H) (I) score plot of OPLS-DA analysis between FA, cvPTC, fvPTC in negative scan mode; (D) (E) (F) (J) (K) (L) 100 permutation test results of the corresponding OPLS-DA score plot. FA: follicular adenoma; cvPTC: classical variant of papillary thyroid cancer; fvPTC: follicular variant of papillary thyroid cancer.

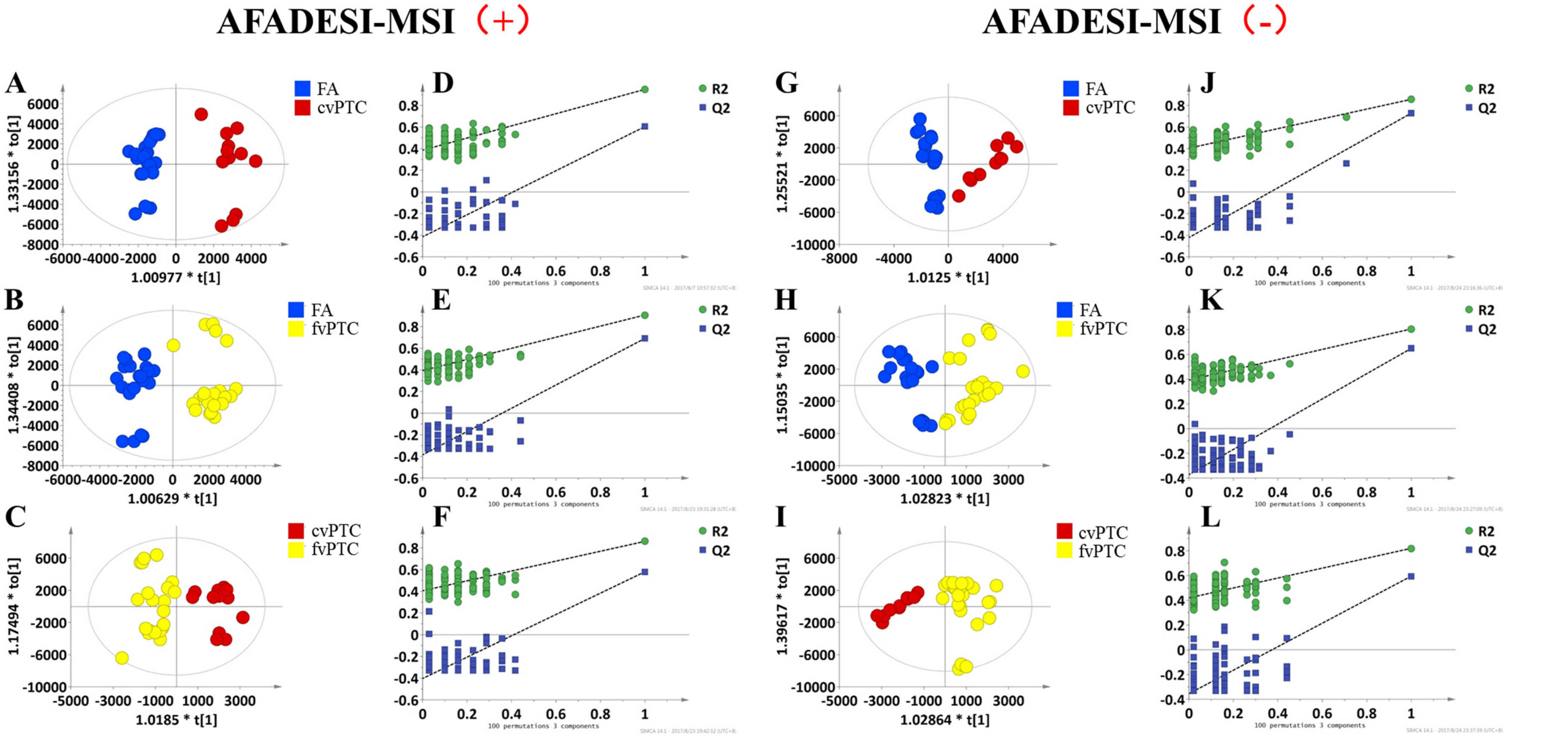

Figure S3. Model classification result of validation set

(A) (B) (C) score plot of OPLS-DA analysis between FA, cvPTC, fvPTC in positive scan mode; (G) (H) (I) score plot of OPLS-DA analysis between FA, cvPTC, fvPTC in negative scan mode; (D) (E) (F) (J) (K) (L) 100 permutation test results of the corresponding OPLS-DA score plot. FA: follicular adenoma; cvPTC: classical variant of papillary thyroid cancer; fvPTC: follicular variant of papillary thyroid cancer.

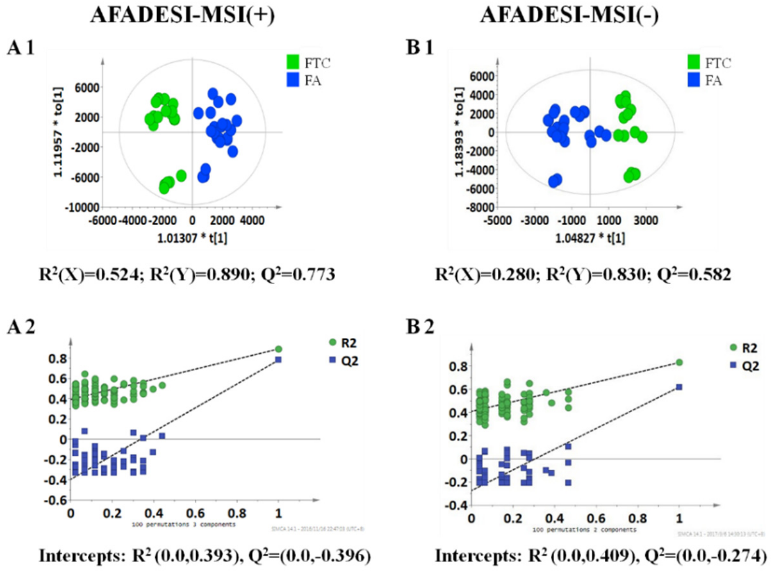

Figure S4. Model classification result for FA and FTC

A1, B1: Score plot of OPLS-DA analysis under positive scan mode and negative scan mode;

A2, B2: 100 random permutation test results in positive scan mode and negative scan mode.

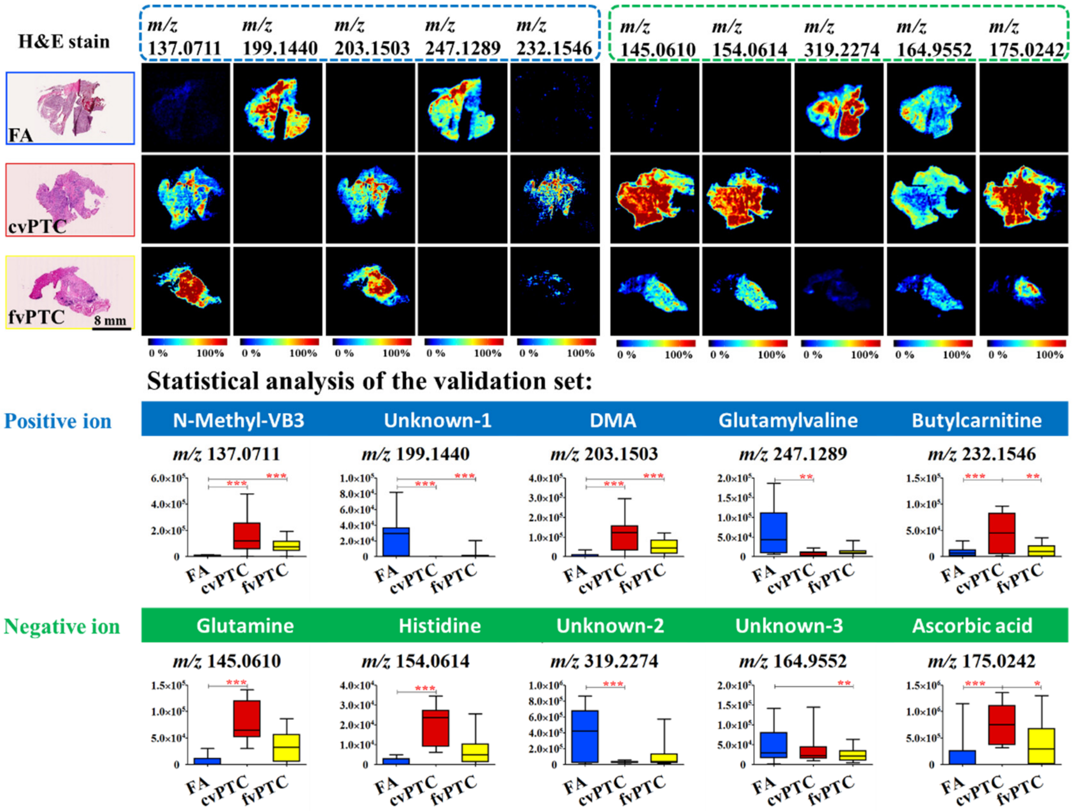

Figure S5. The biomarker panel for diagnosis between benign FA malignant cvPTC and fvPTC in the validation set.

The upper part is the in-situ visualization in different types of thyroid tumor tissue and the lower part is the statistical analysis of each biomarker in box plot. (***p < 0.0001, **p < 0.001, *p < 0.02)

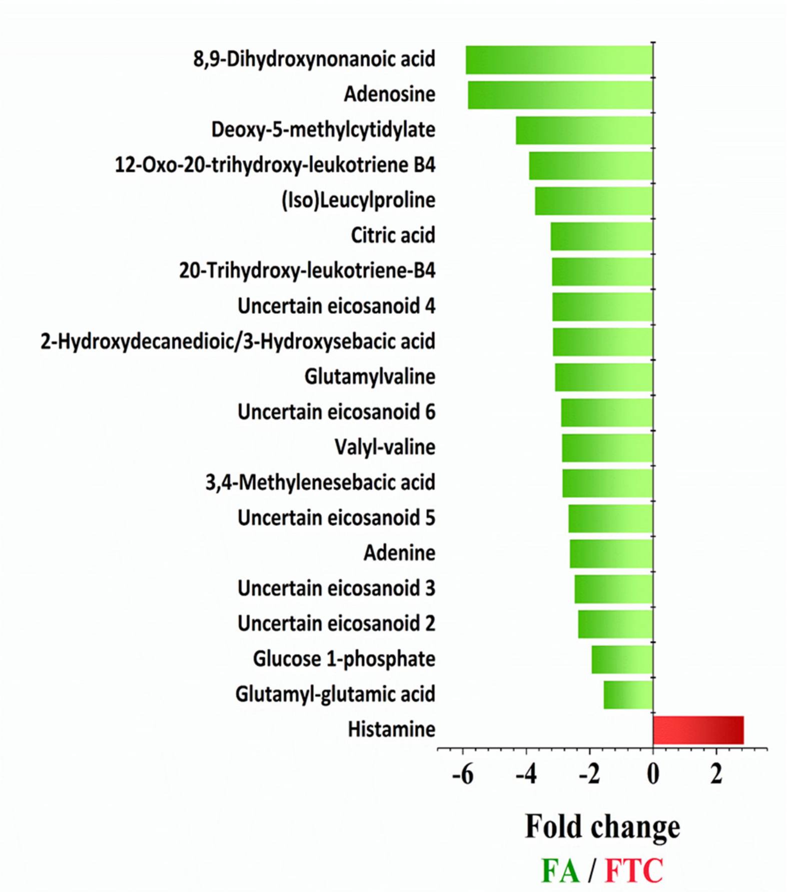

Figure S6. Fold change of differential metabolites in discriminating FA and FTC.

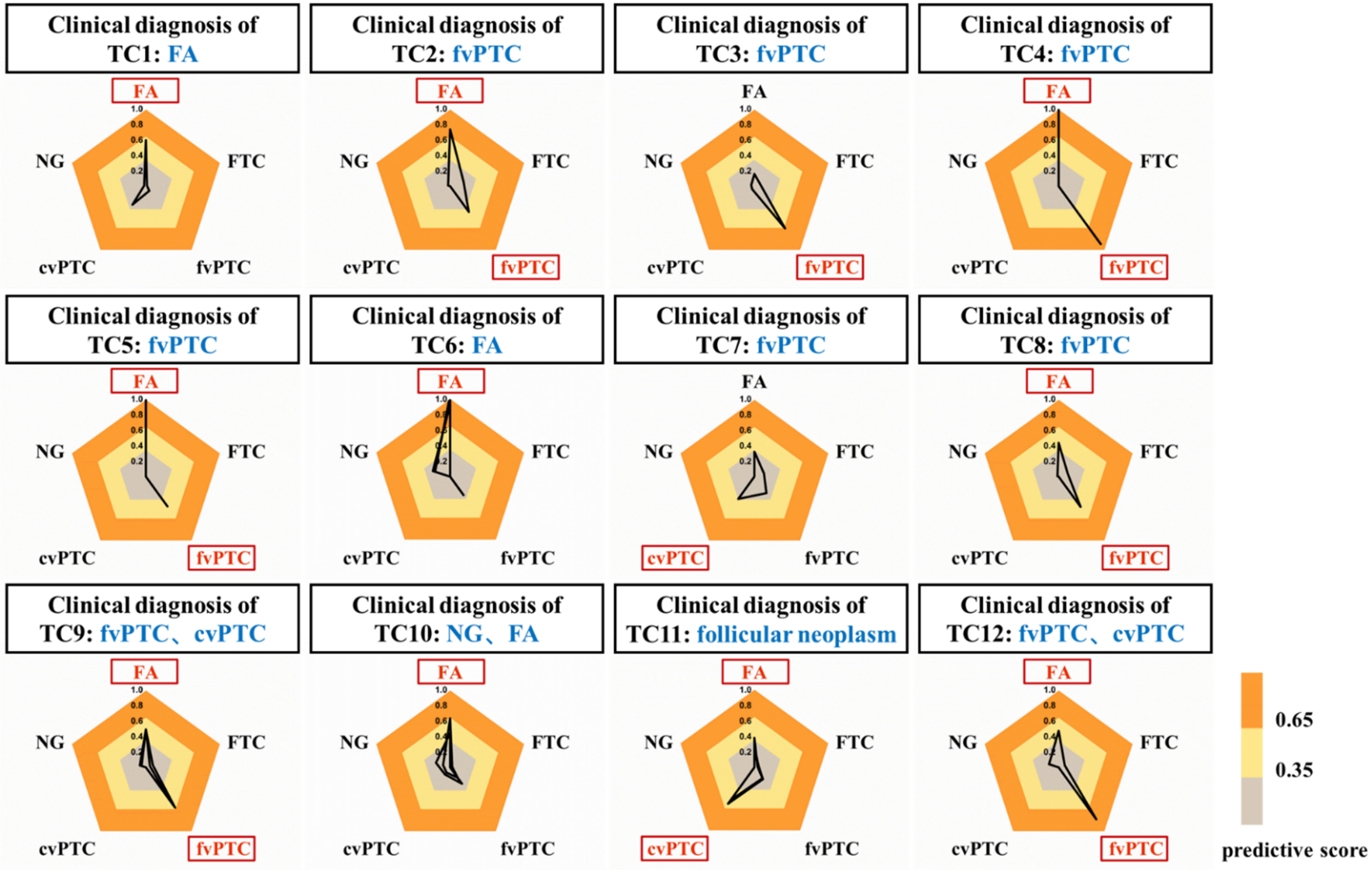

Figure S7. Predictive analysis of each test sample based on the diagnostic OPLS-DA model compared with clinical pathological diagnosis.

The average predicted value was calculated from both results under positive and negative model. If the predicted value is below 0.35, it means it is unlikely to be this class; If the predicted value is between 0.35 and 0.65, it is possible to be this class; if the predicted value is greater than 0.65, it is highly possible to be this class. Malignancy is the basic output if having both benign and malignant predictions.

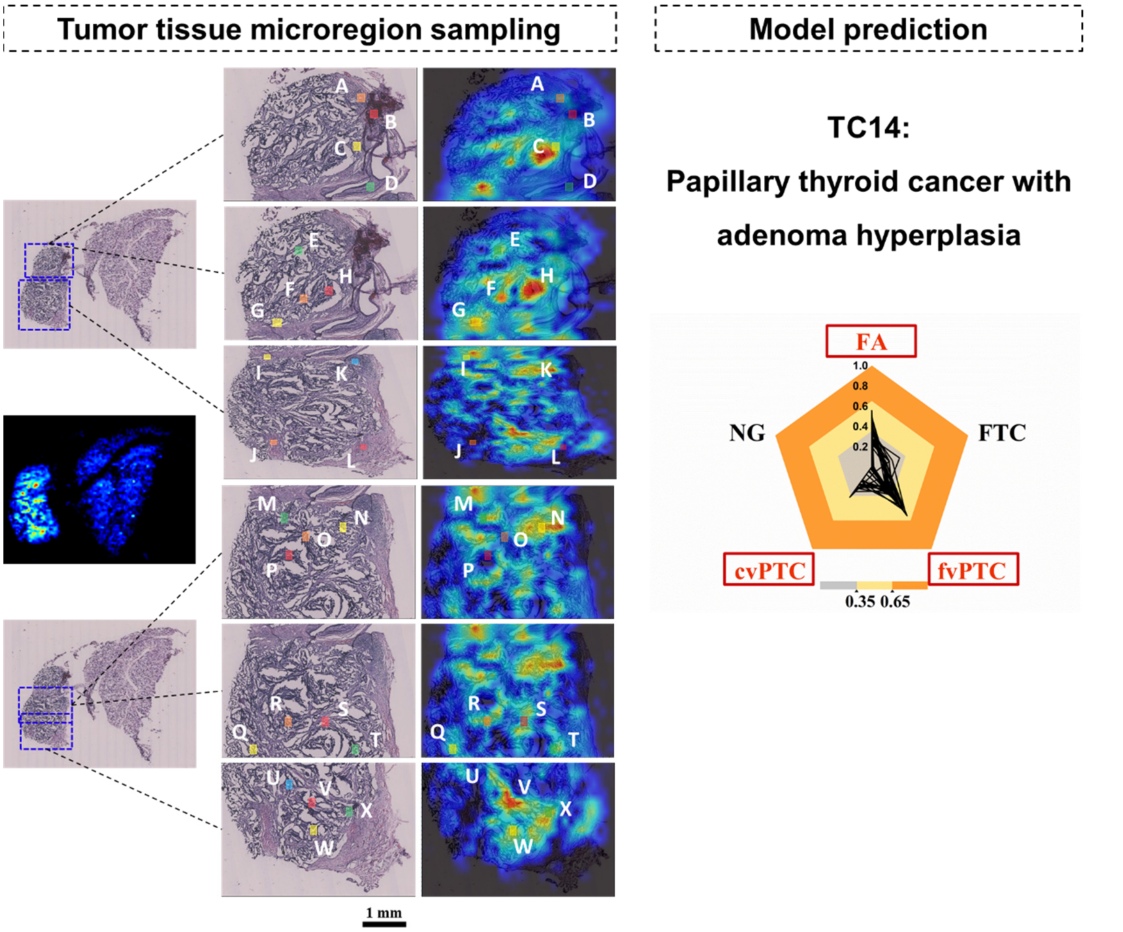

Figure S8. Model prediction analysis of indeterminate case 'TC14'.

Microregion with suspicious foci were delineated on an enlarged view of the H&E staining image. Underneath is the MSI image. The result of computationally predicted value from the integrated model prediction (positive and negative) was shown on the right. The predicted value below 0.35: it does not belong to this classification; The predicted value between 0.35~0.65: it may belong to this classification; The predicted value above 0.65: it belongs to this classification. Malignancy is the basic output if having both benign and malignant predictions.

Table S5 Model cumulative R2X、R2Y and Q2(cum) and Intercepts of R^2 and Q^2 in one hundred times permutations of each classification model between thyroid tumor subtypes

Group	Batch analysis	Scan mode	R^2X	R^2Y	Q^2(cum)	R^2	Q^2
**FA** vs **cvPTC**	Discovery	positive	0.360	0.806	0.659	0.321	-0.254
		negative	0.350	0.783	0.663	0.380	-0.221
	Validation	positive	0.448	0.947	0.760	0.386	-0.414
		negative	0.562	0.855	0.738	0.400	-0.420
**FA** vs **fvPTC**	Discovery	positive	0.292	0.775	0.593	0.352	-0.265
		negative	0.304	0.777	0.602	0.432	-0.257
	Validation	positive	0.507	0.906	0.718	0.394	-0.384
		negative	0.468	0.805	0.647	0.392	-0.371
**fvPTC** vs **cvPTC**	Discovery	positive	0.438	0.717	0.508	0.290	-0.192
		negative	0.376	0.665	0.510	0.389	-0.226
	Validation	positive	0.544	0.859	0.581	0.400	-0.402
		negative	0.526	0.820	0.644	0.420	-0.354
**FA** vs **FTC**	Discovery	positive	0.524	0.890	0.773	0.393	-0.396
		negative	0.280	0.830	0.582	0.409	-0.274

Table S6 Reproducible differential variables for benign (FA) and malignant thyroid tumor (cvPTC□fvPTC)

Differential variables between FA and cvPTC

m/z	scan mode	VIP* 1	VIP* 2	p value 1	p value 2	FC* 1	FC* 2
114.0665	positive	1.41	2.26	1.07E-05	2.47E-04	0.14	0.12
118.0872	positive	2.40	3.15	4.76E-07	3.67E-05	0.18	0.11
127.1232	positive	6.04	2.36	5.87E-10	2.25E-02	0.14	0.43
137.0458	positive	4.36	5.83	2.68E-08	6.55E-05	0.28	0.30
137.0711	positive	3.38	5.14	5.18E-09	5.47E-06	0.10	0.04
139.1230	positive	3.30	2.01	1.96E-07	6.44E-04	0.17	0.44
141.1134	positive	2.88	4.97	2.66E-02	2.25E-02	29.40	7737.55
148.0038	positive	1.43	2.40	1.20E-08	9.00E-08	0.14	0.08
152.0233	positive	1.05	1.35	4.40E-06	3.94E-04	0.16	0.17
155.1290	positive	9.00	8.62	1.41E-04	4.25E-04	13.17	4436.39
159.0277	positive	2.81	4.51	1.03E-05	6.98E-04	0.43	0.39
162.1123	positive	12.64	9.31	1.04E-04	2.52E-04	0.25	0.32
169.0583	positive	1.14	2.07	8.14E-11	5.19E-06	0.06	0.11
175.0015	positive	3.84	5.16	2.87E-04	3.99E-04	0.29	0.30
175.1190	positive	3.82	8.39	1.86E-04	2.72E-06	0.10	0.15
199.1440	positive	3.28	1.77	2.90E-07	2.46E-04	13.00	461.38
203.1503	positive	2.42	4.34	4.81E-04	2.69E-06	0.07	0.05
204.1230	positive	10.87	9.66	3.09E-05	7.64E-06	0.11	0.12
232.1546	positive	2.97	2.32	9.71E-05	2.17E-04	0.21	0.19
239.2229	positive	1.21	2.94	5.24E-06	1.34E-05	0.18	0.18
247.1289	positive	1.67	2.29	1.12E-04	2.21E-03	9.79	6.91
307.0436	positive	3.84	4.12	1.90E-03	3.87E-03	0.36	0.36
317.2081	positive	2.41	1.38	1.53E-03	4.14E-03	5.62	20.44
319.2240	positive	1.72	1.35	2.40E-03	8.98E-03	4.24	26.01
333.2030	positive	2.18	1.22	5.86E-04	8.16E-04	6.57	50.45
335.2190	positive	2.35	1.74	1.33E-03	3.54E-04	4.25	18.06
359.2188	positive	1.76	1.06	4.80E-06	1.23E-03	9.25	225.71
483.0757	positive	3.88	7.35	1.45E-03	4.84E-04	0.54	0.51
124.0056	negative	8.21	7.34	2.18E-07	3.22E-10	0.13	0.09
126.0016	negative	1.69	1.52	3.70E-07	5.83E-10	0.07	0.03
132.0107	negative	3.12	3.04	2.00E-08	7.01E-10	0.10	0.13
135.0295	negative	6.52	3.33	2.54E-06	3.67E-04	0.19	0.53
145.0602	negative	3.43	3.45	1.94E-06	1.51E-09	0.08	0.08
146.0442	negative	7.52	7.24	7.70E-06	5.49E-08	0.11	0.15
164.0701	negative	1.73	2.19	1.46E-05	2.04E-11	0.14	0.13
174.0394	negative	10.79	4.31	2.67E-03	1.08E-07	0.39	0.09
175.0233	negative	20.76	8.34	2.24E-05	5.56E-04	0.21	0.27
226.9955	negative	7.27	2.14	1.44E-04	1.62E-03	0.24	0.31
293.2119	negative	5.04	5.48	1.48E-03	2.16E-03	2.83	12.44
295.2272	negative	6.13	7.03	1.53E-03	5.89E-03	2.96	8.17
309.2067	negative	4.49	4.20	2.70E-04	2.19E-03	3.80	39.34
311.2224	negative	6.94	8.54	8.93E-04	1.14E-03	3.12	28.97
317.2117	negative	3.46	3.89	2.58E-06	9.05E-04	5.34	50.82
319.2274	negative	4.91	6.88	1.57E-05	6.70E-04	3.88	13.29
327.2176	negative	2.88	2.49	1.61E-04	1.43E-03	3.74	970.26
329.2331	negative	3.07	3.56	2.20E-02	1.02E-02	2.54	40.73
331.1912	negative	2.17	1.57	2.89E-06	4.22E-03	6.32	∞
333.2069	negative	2.62	2.47	4.18E-07	6.94E-04	5.18	106.51
335.2226	negative	4.47	6.10	4.75E-06	1.30E-03	4.31	37.99
337.2375	negative	2.34	2.37	4.45E-06	4.02E-03	5.31	42.61
339.2532	negative	1.71	1.62	3.51E-05	2.67E-02	9.10	92.57
345.2433	negative	1.07	1.54	1.07E-02	1.11E-03	2.47	11.87
347.1990	negative	1.49	1.29	8.61E-03	8.10E-03	3.18	∞
353.2327	negative	2.85	3.28	1.82E-05	1.12E-03	4.21	70.74
367.2121	negative	3.83	3.45	3.23E-06	4.94E-03	5.71	3300.41

369.2276	negative	2.06	2.16	1.10E-04	3.02E-03	4.13	13.10
375.2176	negative	1.37	1.34	1.12E-03	6.14E-03	3.19	33.43
381.1915	negative	2.36	1.40	7.94E-06	2.63E-02	5.73	∞
383.2071	negative	2.27	1.59	9.58E-06	1.80E-02	6.32	∞
385.2229	negative	1.14	1.05	1.00E-03	4.99E-03	3.85	∞
700.5280	negative	1.38	1.50	6.65E-06	1.61E-08	0.05	0.06
722.5127	negative	2.33	2.78	1.29E-04	5.15E-05	0.19	0.22
742.5390	negative	2.05	2.65	6.71E-04	6.40E-06	0.20	0.06
750.5436	negative	1.79	2.09	7.84E-05	2.49E-04	0.18	0.22
766.5386	negative	2.20	2.69	5.10E-06	1.87E-05	0.08	0.14
104.0341	negative	2.00	1.92	2.59E-06	5.03E-12	0.07	0.11
111.0182	negative	2.33	1.61	1.27E-05	6.69E-06	0.26	0.36
131.0446	negative	1.19	1.35	2.00E-04	9.20E-08	0.37	0.01
140.0101	negative	3.47	4.50	5.22E-08	3.68E-10	0.04	0.06
154.0605	negative	1.89	1.82	7.00E-07	1.20E-09	0.04	0.06
221.1174	negative	1.64	1.25	2.76E-06	1.53E-03	7.86	209.67
325.2016	negative	2.24	1.66	1.83E-04	1.33E-03	3.59	151.98
361.2384	negative	1.12	1.54	3.52E-03	1.05E-03	2.90	89.81
**Differential variables between FA and fvPTC**							
m/z	**scan mode**	**VIP* 1**	**VIP* 2**	**p value 1**	**p value 2**	**FC* 1**	**FC* 2**
137.0710	positive	1.76	3.63	9.32E-03	2.02E-07	0.30	0.09
142.9479	positive	2.04	1.33	2.44E-03	5.39E-03	2.28	3.02
199.1440	positive	2.45	1.83	1.32E-03	1.85E-05	2.42	8.81
203.1500	positive	1.34	2.69	2.98E-02	1.24E-05	0.20	0.12
154.9265	negative	2.72	2.46	1.12E-02	7.78E-03	1.99	3.10
164.9552	negative	2.26	2.61	3.55E-04	9.66E-03	2.45	1.99
277.0733	negative	2.12	1.88	1.22E-04	1.75E-02	2.84	4.72
307.0840	negative	3.24	1.53	2.95E-04	1.45E-02	6.39	3.99
**Differential variables between cvPTC and fvPTC**							
m/z	**scan mode**	**VIP* 1**	**VIP* 2**	**p value 1**	**p value 2**	**FC* 1**	**FC* 2**
232.1543	positive	3.59	2.44	2.57E-04	1.12E-03	4.66	3.58
175.0233	negative	19.05	7.77	5.58E-04	1.50E-02	2.85	1.97

*FC (fold change) = FA / cvPTC; FA / fvPTC; cvPTC / fvPTC   VIP: Variable importance
1: Discovery set   2: Validation set

Table S7. Reproducible differential variables for benign (FA) and malignant thyroid tumor (FTC)

m/z	scan mode	VIP*	p value	FC*	m/z	scan mode	VIP*	p value	FC*
112.0872	positive	1.32	2.64E-02	0.35	223.0976	negative	2.12	8.19E-04	8.30
113.1077	positive	2.11	2.21E-04	2832.28	225.1134	negative	1.76	1.03E-03	2.86
129.1024	positive	2.45	1.12E-03	14.61	259.0240	negative	1.72	2.92E-02	1.94
141.1134	positive	8.06	1.83E-02	0.23	272.0625	negative	1.99	7.47E-03	11.63
143.1180	positive	2.68	8.28E-04	5.41	296.8611	negative	1.35	1.52E-02	3.06
158.9220	positive	1.12	6.53E-04	2.51	301.8992	negative	1.57	4.32E-03	5.67
159.0277	positive	2.11	3.35E-03	2.50	302.0543	negative	2.01	2.62E-02	2.12
167.1180	positive	1.23	4.67E-04	10.97	307.0853	negative	2.21	1.78E-02	2.86
169.1336	positive	1.87	3.52E-04	9.12	320.0635	negative	2.44	5.90E-04	4.33
177.0884	positive	1.44	1.61E-03	0.39	323.8758	negative	2.20	8.95E-04	5.61
180.9029	positive	2.11	5.77E-04	3.09	330.0776	negative	3.13	2.31E-02	3.27
184.0945	positive	2.57	4.30E-03	2.57	331.1926	negative	1.27	1.69E-02	2.04
187.1443	positive	1.32	4.06E-04	7.44	333.2083	negative	1.78	1.45E-03	2.37
196.8772	positive	1.05	3.56E-03	2.78	335.2238	negative	2.29	2.06E-02	1.79
199.1442	positive	3.41	1.04E-04	3.65	343.0273	negative	1.16	7.19E-03	7.21
211.1441	positive	1.59	1.36E-04	6.64	347.2007	negative	1.66	1.28E-02	4.64
217.1547	positive	1.48	8.39E-04	2.87	349.2035	negative	3.43	1.41E-04	2.67
225.0346	positive	1.07	1.61E-03	2.95	353.2340	negative	2.26	9.01E-04	2.48
226.1052	positive	1.66	2.07E-03	3.59	359.0972	negative	1.48	3.40E-04	8.37
229.1549	positive	2.25	5.51E-03	3.72	367.2138	negative	3.81	3.89E-04	3.18
235.1804	positive	14.82	1.01E-05	0.20	369.2294	negative	2.04	1.34E-03	2.90
237.1869	positive	2.87	5.05E-03	0.45	374.8276	negative	1.05	2.80E-03	14.17
247.1293	positive	2.01	4.50E-03	3.09	381.1938	negative	2.71	2.39E-04	3.91
257.1623	positive	5.09	6.51E-05	0.20	383.2089	negative	2.26	1.28E-03	3.19
265.1910	positive	1.02	2.31E-03	0.29	385.1802	negative	1.36	5.20E-03	12.88
268.1037	positive	1.23	1.42E-04	5.84	385.2246	negative	1.22	4.07E-03	3.14
277.1047	positive	2.40	2.38E-02	1.56	387.1958	negative	1.70	8.20E-03	5.72
280.0919	positive	1.97	9.42E-04	1.92	389.1080	negative	1.28	2.09E-03	3.39
321.1308	positive	2.04	9.99E-03	1.92	393.0744	negative	1.63	6.30E-03	∞
333.1820	positive	1.01	1.15E-02	2.46	395.2457	negative	1.41	2.71E-04	5.43
391.1879	positive	1.15	1.73E-03	3.19	397.1885	negative	1.28	8.60E-04	6.54
391.2565	positive	2.02	7.53E-03	0.21	399.2038	negative	1.52	2.47E-02	2.65
407.1828	positive	1.40	1.09E-04	4.21	403.1899	negative	1.19	1.77E-02	3.98
463.3027	positive	1.20	2.58E-02	0.37	409.2253	negative	1.47	1.31E-04	4.89
491.3353	positive	1.25	5.31E-04	0.04	412.1995	negative	3.36	5.24E-04	12.72
134.0451	negative	1.11	4.04E-03	2.62	414.2152	negative	3.09	8.48E-04	17.24
154.9240	negative	2.73	7.44E-03	2.62	416.2309	negative	1.31	8.20E-03	39.71
159.9644	negative	1.92	4.72E-03	6.33	428.1942	negative	2.23	2.24E-03	16.14
189.1127	negative	2.30	4.50E-04	5.91	430.2097	negative	3.05	2.00E-03	11.42
191.0194	negative	11.84	2.78E-02	3.23	432.2257	negative	1.49	6.63E-03	30.97
210.9629	negative	1.07	4.38E-02	2.81	444.1893	negative	2.48	1.17E-03	11.67
217.1070	negative	2.32	9.65E-03	3.16	446.2050	negative	2.25	3.64E-03	9.26
221.1183	negative	1.09	3.61E-03	3.00					

*FC (fold change) = FA / FTC   VIP: Variable importance

Table S8 Identified biomarkers for diagnosis between benign (FA) and malignant thyroid tumor (cvPTC[illegible]fvPTC)

FA and cvPTC

m/z	Identification	HMDB ID	Adduct Ion	Elemental Composition	Delta ppm	Product Ions	Sub Class
114.0665	Creatinine	HMDB0000562	$[M+H]^+$	$C_4H_7N_3O$	2.731	86.0741, 72.0474	Amino acids derivatives
118.0866	Betaine*	HMDB0000043	$[M+H]^+$	$C_5H_{11}NO_2$	2.920	59.0739, 58.0660	Amino acids derivatives
137.0458	Hypoxanthine*	HMDB0000157	$[M+H]^+$	$C_5H_4N_4O$	0.092	119.0351, 110.0348, 94.0403, 82.0406	Purines derivatives
137.0711	N-Methylnicotinamide	HMDB0003152	$[M+H]^+$	$C_7H_8N_2O$	1.171	120.0440, 110.0600, 56.0500	Nicotinamides (Pyridines derivatives)
141.1134	Methenamine	HMDB0029598	$[M+H]^+$	$C_6H_{12}N_4$	-0.517	112.0865, 71.0606, 58.0658	Polyamine
162.1123	Carnitine*	HMDB0000062	$[M+H]^+$	$C_7H_{15}NO_3$	-1.048	103.0394, 102.0920, 85.0291, 60.0819	Carnitines
169.0583	Glutamine*	HMDB0000641	$[M+Na]^+$	$C_5H_{10}N_2O_3$	-0.375	84.0452, 130.0503	Amino acids
175.1190	Arginine	HMDB0000517	$[M+H]^+$	$C_6H_{14}N_4O_2$	0.273	158.0927, 130.0983, 116.0705, 70.0656, 60.0563	Amino acids
203.1503	Dimethylarginine*	HMDB0001539	$[M+H]^+$	$C_8H_{18}N_4O_2$	0.234	158.1285, 116.0708, 88.0875, 70.0657	Amino acids
204.1230	Acetylcarnitine*	HMDB0000201	$[M+H]^+$	$C_9H_{17}NO_4$	-0.169	145.0488, 144.1013, 85.0275, 60.0815	Carnitines
232.1546	Butyryl-L-carnitine*	HMDB0002013	$[M+H]^+$	$C_{11}H_{21}NO_4$	1.143	173.0821, 85.0295, 60.0818	Carnitines
247.1289	Glutamylvaline	HMDB0028832	$[M+H]^+$	$C_{10}H_{18}N_2O_5$	1.828	229.1185, 211.1077, 130.0503, 112.0400, 102.0556, 85.0292, 84.0452,	Dipeptides
124.0063	Taurine*	HMDB0000251	$[M-H]^-$	$C_2H_7NO_3S$	-8.766	106.9814, 94.9808, 79.9570, 64.9709	Organic acids
104.0341	Serine	HMDB0000187	$[M-H]^-$	$C_3H_7NO_3$	-11.692	74.0250, 58.0304	Amino acids
111.0189	Uracil*	HMDB0000300	$[M-H]^-$	$C_4H_4N_2O_2$	-9.914	67.0172	Pyrimidines and pyrimidine Derivatives
131.0452	Asparagine*	HMDB0000168	$[M-H]^-$	$C_4H_8N_2O_3$	-6.222	131.0454, 114.0193, 113.0341, 70.0304	Amino acids
135.0305	Hypoxanthine*	HMDB0000157	$[M-H]^-$	$C_5H_4N_4O$	-5.436	106.0274, 92.0250, 64.0145	Purines derivatives
140.0110	O-Phosphorylethanolamine*	HMDB0000224	$[M-H]^-$	$C_2H_8NO_4P$	-5.839	78.9595	Glycerophospholipids
145.0610	Glutamine*	HMDB0000641	$[M-H]^-$	$C_5H_{10}N_2O_3$	-5.966	127.0501, 109.0401, 84.0449, 74.0254	Amino acids
146.0451	Glutamate*	HMDB0000148	$[M-H]^-$	$C_5H_9NO_4$	-5.348	102.0559, 128.0347, 85.0267	Amino acids
154.0614	Histidine*	HMDB0000177	$[M-H]^-$	$C_6H_9N_3O_2$	-5.192	137.0358, 110.0712, 93.0457, 81.0463	Amino acids
164.0709	Phenylalanine*	HMDB0000159	$[M-H]^-$	$C_9H_{11}NO_2$	-4.888	147.0445, 103.0549, 72.0094	Amino acids
174.0401	N-acetylaspartate*	HMDB0000812	$[M-H]^-$	$C_6H_9NO_5$	-3.997	130.0491, 114.0179, 88.0397, 58.0297,	Amino acids derivatives
175.0242	Ascorbic acid*	HMDB0000044	$[M-H]^-$	$C_6H_8O_6$	-3.492	115.0020, 87.0082, 71.0135, 59.0133	Vitamin C
293.2119	9-OxoODE; 9/13-HOTE; 9(10)/15(16)-EpODE	\	$[M-H]^-$	$C_{18}H_{30}O_3$	-1.085	249.2243, 185.1158, 125.0969, 59.0137, 57.0349	Long-chain fatty acids
295.2272	9/13-HODE; 9,10-Epoxyoctadecenoic acid;	\	$[M-H]^-$	$C_{18}H_{32}O_3$	-2.263	277.2176, 195.1403, 171.1041, 113.0946, 59.0137	Long-chain fatty acids

	13S-hydroxyoctadecadienoic acid						
309.2067	12(13)Ep-9-KODE	HMDB0013623	$[M-H]^-$	$C_{18}H_{30}O_4$	-1.399	295.1965, 209.1183, 193.1234, 171.1026, 137.0971	Long-chain fatty acids
311.2224	9-HPODE	HMDB0006940	$[M-H]^-$	$C_{18}H_{32}O_4$	-1.230	293.2122, 211.1352, 197.1202, 185.1193, 183.0122, 171.1031	Long-chain fatty acids
317.2117	one of the eicosanoid 1	\	$[M-H]^-$	$C_{20}H_{30}O_3$	-1.633	273.2230, 179.1074, 147.1163, 139.1113, 115.0385, 113.0956	Long-chain fatty acids
329.2331	9,10,13-TriHOME	HMDB0004710	$[M-H]^-$	$C_{18}H_{34}O_5$	-0.751	229.1445, 211.1339, 171.1026, 139.1128, 127.1120, 99.0815	Long-chain fatty acids
333.2083	one of the eicosanoid 2	\	$[M-H]^-$	$C_{20}H_{30}O_4$	3.503	317.2119, 209.1174, 193.1227, 173.1326, 139.1116, 59.0120	Long-chain fatty acids
353.2327	one of the eicosanoid 3	\	$[M-H]^-$	$C_{20}H_{34}O_5$	-1.833	317.2122, 167.1077, 127.0764,	Long-chain fatty acids
367.2121	one of the eicosanoid 4	\	$[M-H]^-$	$C_{20}H_{32}O_6$	-1.394	305.2122, 99.0452, 59.0139	Long-chain fatty acids
381.1933	12-Oxo-20-trihydroxy-leukotriene B4	HMDB0012553	$[M-H]^-$	$C_{20}H_{30}O_7$	3.734	319.1950, 301.1796, 235.0998, 205.0859, 151.1115, 123.0803, 59.0121	Long-chain fatty acids
383.2089	20-Trihydroxy-leukotriene-B4	HMDB12643	$[M-H]^-$	$C_{20}H_{32}O_7$	3.584	177.0909, 155.0701, 115.0384, 99.0434, 59.0120	Long-chain fatty acids
722.5127	PE(36：4)*	HMDB0009150	$[M-H]^-$	$C_{41}H_{74}NO_7P$	-0.434	303.2329, 259.2439	Glycerophospholipids
742.5390	PE(36：2)	HMDB0008843	$[M-H]^-$	$C_{41}H_{78}NO_8P$	-0.307	281.2480, 140.0101	Glycerophospholipids
750.5436	PE(P-38：4)	HMDB0005779	$[M-H]^-$	$C_{43}H_{78}NO_7P$	-0.951	303.2329	Glycerophospholipids
766.5386	PE(38：4)*	HMDB0009583	$[M-H]^-$	$C_{43}H_{78}NO_8P$	-0.819	303.2329, 281.2480	Glycerophospholipids

FA and fvPTC

m/z	**Identification**	**HMDB ID**	**Adduct Ion**	**Elemental Composition**	**Delta ppm**	**Product Ions**	**Sub Class**
137.0710	N-Methylnicotinamide	HMDB0003152	$[M+H]^+$	$C_7H_8N_2O$	0.442	120.0440, 110.0600, 56.0500	Nicotinamides (pyridines derivatives)
203.1500	Dimethylarginine*	HMDB0001539	$[M+H]^+$	$C_8H_{18}N_4O_2$	-1.242	158.1285, 116.0708, 88.0875, 70.0657	Amino acids

cvPTC and fvPTC

m/z	**Identification**	**HMDB ID**	**Adduct Ion**	**Elemental Composition**	**Delta ppm**	**Product Ions**	**Sub Class**
232.1543	Butyryl-L-carnitine*	HMDB0002013	$[M+H]^+$	$C_{11}H_{21}NO_4$	-0.149	173.0821, 85.0295, 60.0818	Carnitines
175.0242	Ascorbic acid*	HMDB0000044	$[M-H]^-$	$C_6H_8O_6$	-3.492	115.0020, 87.0082, 71.0135, 59.0133	Vitamin C

* Metabolites have been confirmed using standard compounds.

Table S9 Identified biomarkers for diagnosis between follicular adenoma (FA) and follicular thyroid cancer (FTC)

m/z	Identification	HMDB ID	Adduct Ion	Elemental Composition	Delta ppm	Product Ions	Sub Class
112.0872	Histamine*	HMDB0000870	$[M+H]^+$	$C_5H_9N_3$	2.463	95.0612, 83.0609, 68.0515	Amino acids derivatives
217.1547	Valyl-valine	HMDB0029140	$[M+H]^+$	$C_{10}H_{20}N_2O_3$	0.143	200.1289, 171.1490, 118.0860, 100.0760	Dipeptides
229.1549	(Iso)leucylproline	HMDB0011175/4	$[M+H]^+$	$C_{11}H_{20}N_2O_3$	1.008	211.1441, 114.0919, 86.0970	Dipeptides
247.1293	Glutamylvaline	HMDB0028832	$[M+H]^+$	$C_{10}H_{18}N_2O_5$	1.828	229.1185, 211.1077, 130.0503, 112.0400, 102.0556, 85.0292, 84.0452, 70.0660	Dipeptides
268.1037	Adenosine*	HMDB0000050	$[M+H]^+$	$C_{10}H_{13}N_5O_4$	-1.232	136.0622, 119.0358	Purine nucleosides
277.1044	Glutamyl-glutamic acid	HMDB0028818	$[M+H]^+$	$C_{10}H_{16}N_2O_7$	4.954	260.0771, 214.0718, 148.0610, 130.0505, 102.0557, 84.0444	Amino acids
134.0463	Adenine*	HMDB0000034	$[M-H]^-$	$C_5H_5N_5$	-6.852	107.0346, 92.0237, 80.0237, 65.0127	Purine derivatives
189.1127	8,9-Dihydroxynonanoic acid	HMDB0000666	$[M-H]^-$	$C_9H_{17}O_4$	-2.815	129.0902, 73.0275, 59.0120	Medium-chain fatty acids
191.0194	Citric acid*	HMDB0000094	$[M-H]^-$	$C_6H_8O_7$	-1.706	173.0075, 111.0072, 87.0070, 85.0296	Organic acids
217.1081	2-Hydroxydecanedioic acid /3-Hydroxysebacic acid	HMDB00350/424	$[M-H]^-$	$C_{10}H_{18}O_5$	-0.216	181.0875, 157.0974	Medium-chain fatty acids
225.1134	3,4-Methylenesebacic acid	HMDB0059729	$[M-H]^-$	$C_{12}H_{18}O_4$	0.745	207.1013, 59.0118	Medium-chain fatty acids
259.0230	Glucose 1-phosphate	HMDB0001586	$[M-H]^-$	$C_6H_{13}O_9P$	2.156	259.0221, 241.0103, 138.9786, 96.9694, 78.9573	Monosaccharide
320.0639	Deoxy-5-methylcytidylate		$[M-H]^-$	$C_{10}H_{16}N_3O_7P$	-4.404	302.0517, 284.0412, 240.0509, 216.0506, 196.0603, 128.0338, 110.0230, 59.0120	Pyrimidine derivatives
333.2083	one of the eicosanoid 2		$[M-H]^-$	$C_{20}H_{30}O_4$	3.503	317.2119, 273.2230, 235.1354, 209.1174, 193.1227, 173.1326, 139.1116, 59.0120	Long-chain fatty acids
349.2035	one of the eicosanoid 5		$[M-H]^-$	$C_{20}H_{30}O_5$	4.160	305.1771, 269.1919, 181.1225, 155.1064, 137.0957, 95.0484, 84.0199, 59.0120	Long-chain fatty acids
353.2340	one of the eicosanoid 3		$[M-H]^-$	$C_{20}H_{34}O_5$	1.848	235.1340, 197.1173, 167.1065, 139.1113, 99.0797, 85.0277, 59.0120	Long-chain fatty acids

367.2138	one of the eicosanoid 4		[M-H]-	$C_{20}H_{32}O_6$	3.235	305.2127, 289.1811, 245.1910, 235.0975, 187.0967, 157.0858, 109.0642, 59.0120	Long-chain fatty acids
369.2294	one of the eicosanoid 6		[M-H]-	$C_{20}H_{34}O_6$	3.082	195.1017, 169.0858, 157.0852, 115.0379	Long-chain fatty acids
381.1933	12-Oxo-20-trihydroxy-leukotriene B4	HMDB0012553	[M-H]-	$C_{20}H_{30}O_7$	3.734	319.1950, 301.1796, 235.0998, 205.0859, 151.1115, 123.0803, 59.0121	Long-chain fatty acids
383.2089	20-Trihydroxy-leukotriene-B4	HMDB12643	[M-H]-	$C_{20}H_{32}O_7$	3.584	177.0909, 155.0701, 115.0384, 99.0434, 59.0120	Long-chain fatty acids

* Metabolites have been confirmed using standard compounds.

Table S10 Receiver operating characteristic curves (ROC) analysis result of potential diagnostic biomarkers

Biomarker	m/z	Mode	Specificity	Sensitivity	AUC value	Group
N-Methylnicotinamide	137.0711	positive	90.90%	76.90%	0.846	FA vs PTC
Unknown-1	199.1440	positive	80.80%	77.30%	0.743	FA vs PTC
Dimethylarginine	203.1503	positive	90.90%	69.20%	0.772	FA vs PTC
Butyryl-L-carnitine	232.1546	positive	88.60%	69.00%	0.806	FA vs cvPTC
			88.40%	71.40%	0.821	cvPTC vs fvPTC
Glutamylvaline	247.1289	positive	93.10%	68.20%	0.857	FA vs cvPTC
Glutamine	145.0610	negative	100.0%	82.80%	0.945	FA vs cvPTC
Histidine	154.0614	negative	95.50%	89.70%	0.930	FA vs cvPTC
Unknown-2	319.2274	negative	89.70%	81.80%	0.855	FA vs cvPTC
Unknown-3	164.9552	negative	63.00%	78.70%	0.678	FA vs fvPTC
Ascorbic acid	175.0242	negative	86.40%	93.10%	0.921	FA vs cvPTC
			76.70%	74.30%	0.803	cvPTC vs fvPTC
8,9-Dihydroxynonanoic acid	189.1127	negative	100.0%	60.00%	0.800	FA vs FTC
Deoxy-5-methylcytidylate	320.0635	negative	100.0%	65.00%	0.835	FA vs FTC
Citric acid	191.0194	negative	76.90%	85.00%	0.831	FA vs FTC

Table S11 Predictive score results of suspicious focuses in indeterminate case 'TC14' based on OPLS-DA model

	Positive mode				Negative mode				Computational prediction		
	Class FA	Class FTC	Class fvPTC	Class cvPTC	Class FA	Class FTC	Class fvPTC	Class cvPTC	Class FA	Class FTC	Class PTC
**TC14-P-1-A**	0.6649	0.0434	-0.3806	**0.672**	0.0932	0.1874	**0.9044**	-0.1851	0.3791	0.1154	**0.5055**
**TC14-P-1-B**	0.9989	-0.1079	-0.2381	0.3472	0.0050	0.3401	**0.9813**	-0.3264	**0.5019**	0.1161	0.3820
**TC14-P-1-C**	0.6388	-0.0263	-0.0867	0.4743	0.1244	0.2287	**0.8102**	-0.1633	0.3816	0.1012	**0.5172**
**TC14-P-1-D**	0.7153	0.0657	0.0523	0.1666	-0.0048	0.3583	**1.0920**	-0.4456	0.3553	0.2120	**0.4327**
**TC14-P-2-E**	0.6415	0.0543	-0.3364	0.6406	0.1877	0.2057	**0.8339**	-0.2272	0.4146	0.1300	**0.4554**
**TC14-P-2-F**	0.4871	0.0026	0.0845	0.4258	0.0534	0.2997	**0.9848**	-0.3379	0.2703	0.1511	**0.5786**
**TC14-P-2-G**	0.6682	-0.1272	0.1554	0.3036	-0.0732	0.1511	**0.8760**	0.0461	0.2975	0.0120	**0.6906**
**TC14-P-2-H**	0.5665	-0.0324	0.0539	0.4120	0.0271	0.2954	**0.9917**	-0.3142	0.2968	0.1315	**0.5717**
**TC14-P-3-I**	0.3858	0.1154	-0.2716	**0.770**	0.1884	0.1281	**0.8531**	-0.1696	0.2871	0.1217	**0.5911**
**TC14-P-3-J**	0.9517	-0.0193	-0.3551	0.4227	0.0384	0.2980	**0.9805**	-0.3169	**0.4950**	0.1393	0.3656
**TC14-P-3-K**	0.7144	-0.0585	-0.2019	0.5460	0.2510	0.0871	**0.8759**	-0.2140	0.4827	0.0143	**0.5030**
**TC14-P-3-L**	0.7425	-0.0978	-0.0747	0.4299	0.0195	0.2941	**0.9372**	-0.2508	0.3810	0.0982	**0.5208**
**TC14-P-4-M**	0.6794	-0.0551	-0.2518	0.6276	0.0439	0.3247	**0.8174**	-0.1860	0.3616	0.1348	**0.5036**
**TC14-P-4-N**	0.7827	-0.0612	-0.1564	0.4349	-0.0014	0.0766	**1.3174**	-0.3926	0.3907	0.0077	**0.6016**
**TC14-P-4-O**	0.4717	0.0225	0.0993	0.4066	0.0226	0.2677	**1.0866**	-0.3769	0.2471	0.1451	**0.6078**
**TC14-P-4-P**	0.6455	0.0674	-0.1998	0.4869	-0.0860	0.5107	**0.7240**	-0.1486	0.2797	0.2890	**0.4313**
**TC14-P-5-Q**	0.6254	-0.0063	-0.2107	0.5916	0.2731	0.2029	**0.6703**	-0.1463	0.4493	0.0983	**0.4525**
**TC14-P-5-R**	0.6977	-0.0168	-0.5033	**0.822**	0.0843	0.3830	0.6203	-0.0876	0.3910	0.1831	**0.4259**
**TC14-P-5-S**	0.4145	0.0519	-0.1576	**0.691**	-0.0184	0.1959	**1.0911**	-0.2687	0.1981	0.1239	**0.6780**
**TC14-P-5-T**	0.4941	0.0818	-0.2091	0.6332	-0.0474	0.2794	**0.8889**	-0.1210	0.2234	0.1806	**0.5961**
**TC14-P-6-U**	0.5164	0.0515	-0.2541	**0.686**	0.2579	0.2081	**0.6989**	-0.1649	0.3872	0.1298	**0.4830**
**TC14-P-6-V**	0.7136	-0.0052	-0.2638	0.5554	0.0595	0.2844	**0.9377**	-0.2816	0.3866	0.1396	**0.4738**
**TC14-P-6-W**	0.6219	-0.0122	-0.1724	0.5627	0.4896	0.0516	0.4494	0.0094	**0.5558**	0.0197	0.4245
**TC14-P-6-X**	0.5619	0.0268	-0.1110	0.5223	0.0239	0.3337	**0.8422**	-0.1998	0.2929	0.1802	**0.5269**

Table S12 A summary of diagnostic studies performed on thyroid tumor using mass spectrometry imaging technique [1]

Reference	Thyroid tissue type	Analytical method	Main results
Ishikawa et al., 2012[2]	Papillary thyroid cancer(PTC),<br>normal thyroid tissues(N)	MALDI-MSI	Discrimination between PTC and N<br>Potential biomarkers:<br>(i) PTC > HC: PC(34:1), PC(34:2), SM(34:1)
Martin Nipp et al., 2012[3]	Papillary thyroid cancer(PTC)<br>with lymph node metastasis (N1) and<br>without lymph node metastasis (N0))	MALDI-MSI	Discrimination between metastatic and nonmetastatic tumors<br>Potential biomarkers:<br>(i) PTC-N1 > PTC-N0: thioredoxin, S100-A10, S100-A6
Guo et al., 2014[4]	Papillary thyroid cancer(PTC),<br>follicular thyroid cancer(FTC),<br>benign thyroid tumor<br>(thyroid adenoma, multinodular goiter),<br>healthy control(HC)	MALDI-MSI	Discrimination between malignant (MTC) and benign thyroid tumors (BTT), as well as healthy control(HC)<br>Potential biomarkers:<br>(i) MTC, BTT vs HC: PC(34:1)<br>(ii) BTT vs MTC, HC: SM(34:1), PA(36:3)<br>(iii) MTC vs BTT, HC: PC(34:1), PA(36:3), SM(34:1)
Kyueng-Whan Min et al., 2014[5]	Papillary thyroid cancer(PTC),<br>normal thyroid tissues(N)	MALDI-MSI	Discrimination between PTC and N<br>Potential biomarkers:<br>(i) PTC > N: ribosomal protein P2
Fabio Pagni et al.,2016[6]	Papillary thyroid cancer(PTC),<br>benign thyroid tumors(BTT)	MALDI-MSI	Discrimination between PTC and BTT<br>Potential biomarkers:<br>(i) PTC > BTN: PEBP1, ROA2
Monika Pietrowska et al., 2016[7]	Anaplastic thyroid cancer (ATC),<br>follicular thyroid cancer (FTC),<br>papillary thyroid cancer (cv/fvPTC)<br>(classic variant and follicular variant)<br>medullary thyroid cancer (MTC)	MALDI-MSI	Discrimination between MTC and malignancies derived from thyroid epithelium, ATC and differentiated cancers, MTC and ATC, FTC and PTC<br>Potential biomarkers:<br>(i) FTC vs PTC: 14-3-3 isoforms, ANXA5, TUBA1B, PRX6, A1AT, SELENBP1, PDIp

Manuel Galli et al., 2016[8]	Papillary thyroid cancer(PTC),<br>follicular adenomas (FA),<br>hyperplastic nodules (HP)	MALDI-MSI	Discrimination between benign thyroid nodules (BTN) and malignant (PTC) lesions, hyperplastic lesions (HP) and follicular adenomas (FA)<br>Potential biomarkers:<br>(i) PTC > BTN: Fibronectin (FINC), Cytoplasmic Actin1 (ACTB1), Prelamin-A/C (LMNA), Heat Shock cognate 71KDa Protein (HSP7C), Adenylate Cyclase Isoenzyme 1 (KAD1)<br>(ii) FTC > HP: MARCS, CATB, CAND1, TR150<br>(iii) HP > FTC: THYG, PHB, ZN484, TFDP3, NID2 and AATC
Jialing Zhang et al.,2016[9]	Oncocytic thyroid tumors<br>(Hurthle cell adenomas and carcinomas),<br>nononcocytic thyroid tumors<br>(PTC and FA) ,<br>normal thyroid tissues	DESI-MSI	Discrimination between Oncocytic thyroid tumors (O), nononcocytic thyroid tumors (NO) and normal thyroid tissues (N)<br>Potential biomarkers:<br>(i) O > NO > N: cardiolipins (CLs), MLCL, ox-CL, CL＋PC, CL＋DG
Jialing Zhang et al.,2017[10]	Lymph node with metastatic papillary thyroid cancer (metastatic PTC)，<br>normal human lymph nodes(NLN)	DESI-MSI	Discrimination between Lymph node with metastatic papillary thyroid cancer and normal human lymph nodes<br>Potential biomarkers:<br>(i) metastatic PTC > NLN: higher unsaturation in GP species with longer FA carbon chains<br>(ii) metastatic PTC < NLN: lower unsaturation in GP species with shorter FA carbon chains
Rachel J. DeHoog et al.,2019[11]	follicular adenomas (FA),<br>follicular thyroid cancer (FTC),<br>Papillary thyroid cancer(PTC),<br>normal thyroid tissues	DESI-MSI	Discrimination between FA and FTC, FA and PTC<br>Potential biomarkers:<br>(i) FA vs FTC: FA (20:4), PS (36:1), PS (36:2), PG (32:1), PI (38:4), Cer (d36:1), PE (36:1), succinate, malate.<br>(ii) FA vs PTC: PS (36:2), Cer (d42:0), PE (38:4), PI (36:1), PA (36:1), CL (74:8),

Supplementary Appendix
Metabolite identification
(1) Metabolite identification of 8,9-dihydroxynonanoic acid

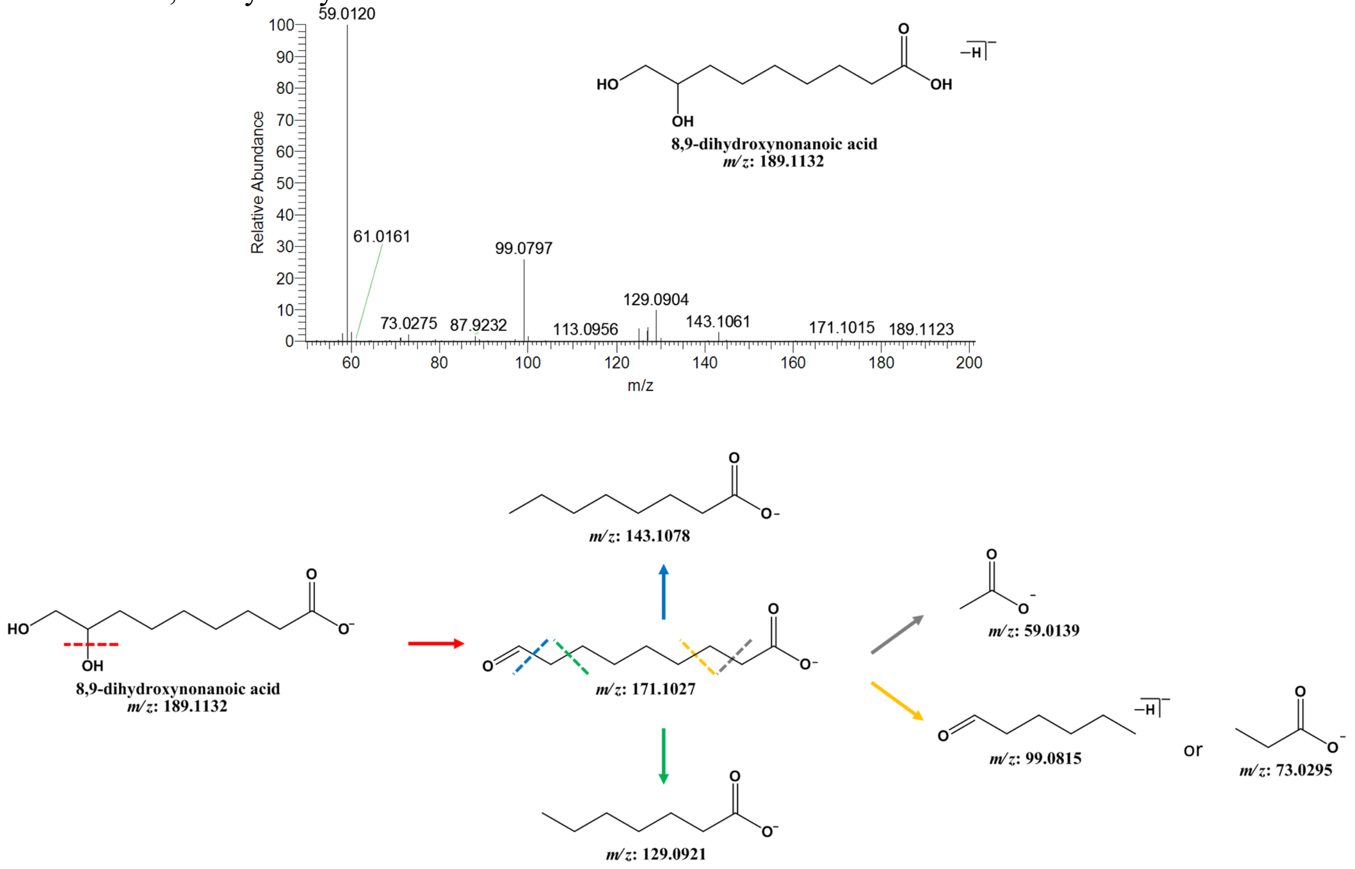

Figure S9. The MS/MS spectrum at *m/z* 189.1123 identified as 8,9-dihydroxynonanoic acid and its potential (ESI) MS fragment pathway (below).

(2) Metabolite identification by standards

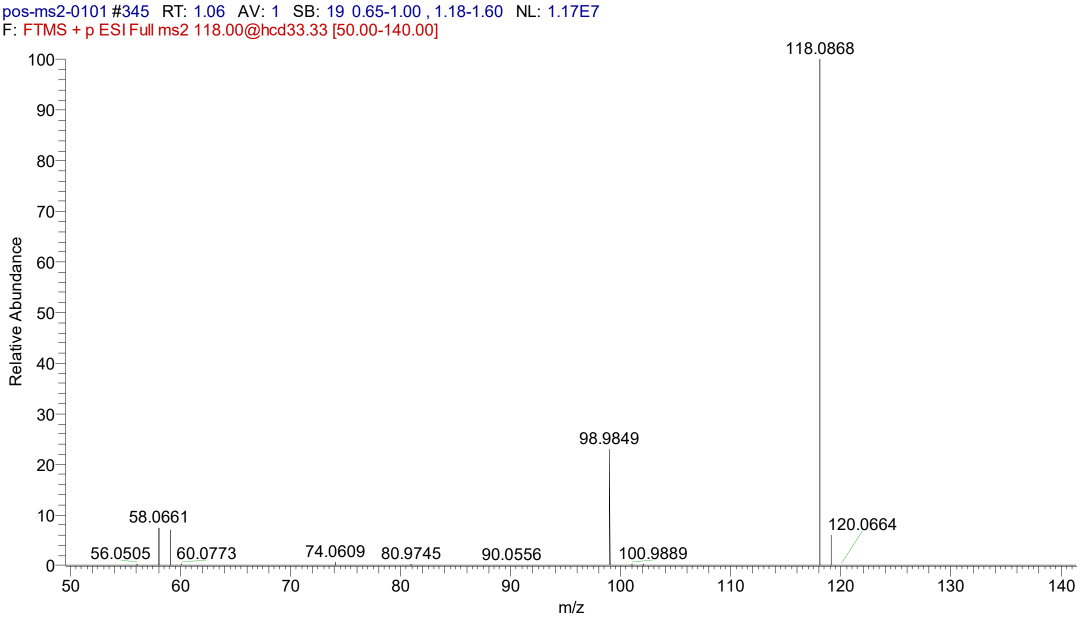

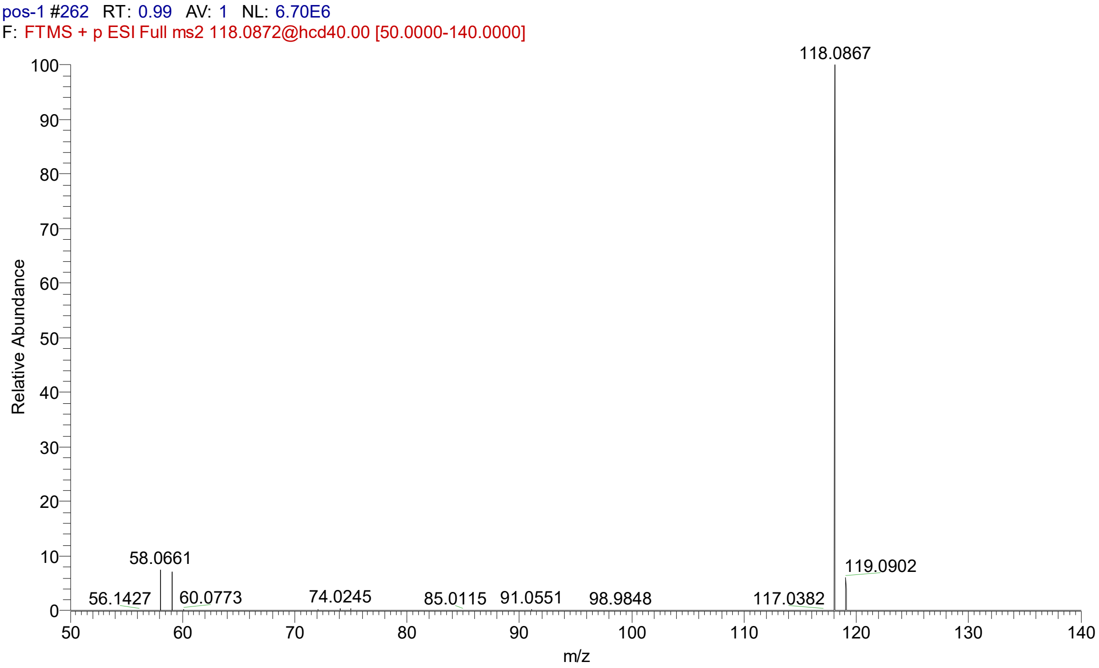

Figure S10. The MS/MS spectrum at *m/z* 118.0868 (Table S10) identifies it as betaine when compared with the standard given in the standard betaine (below).

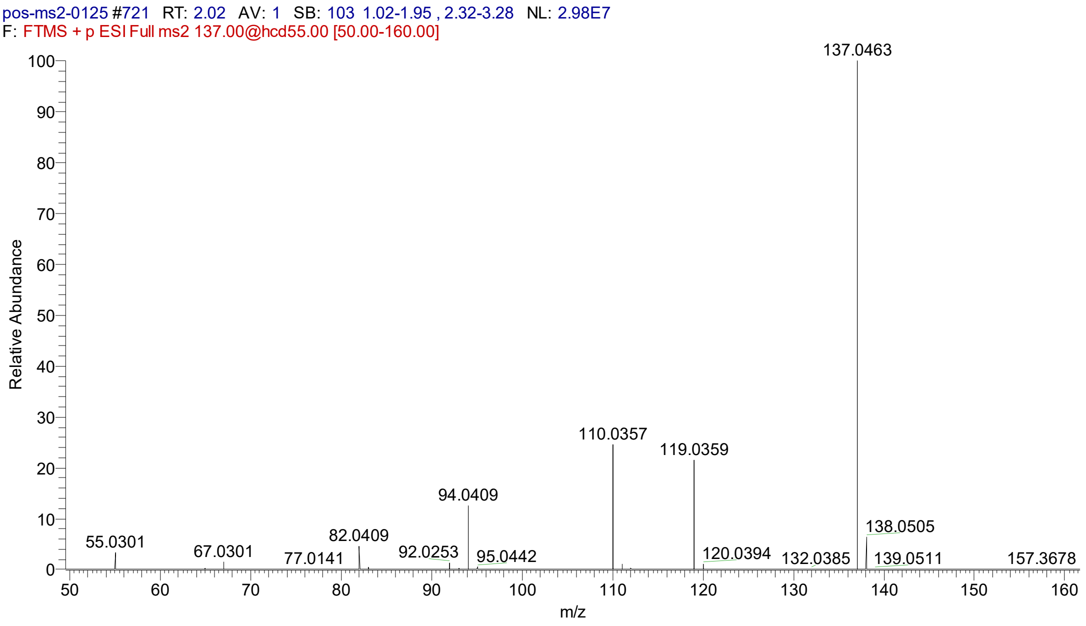

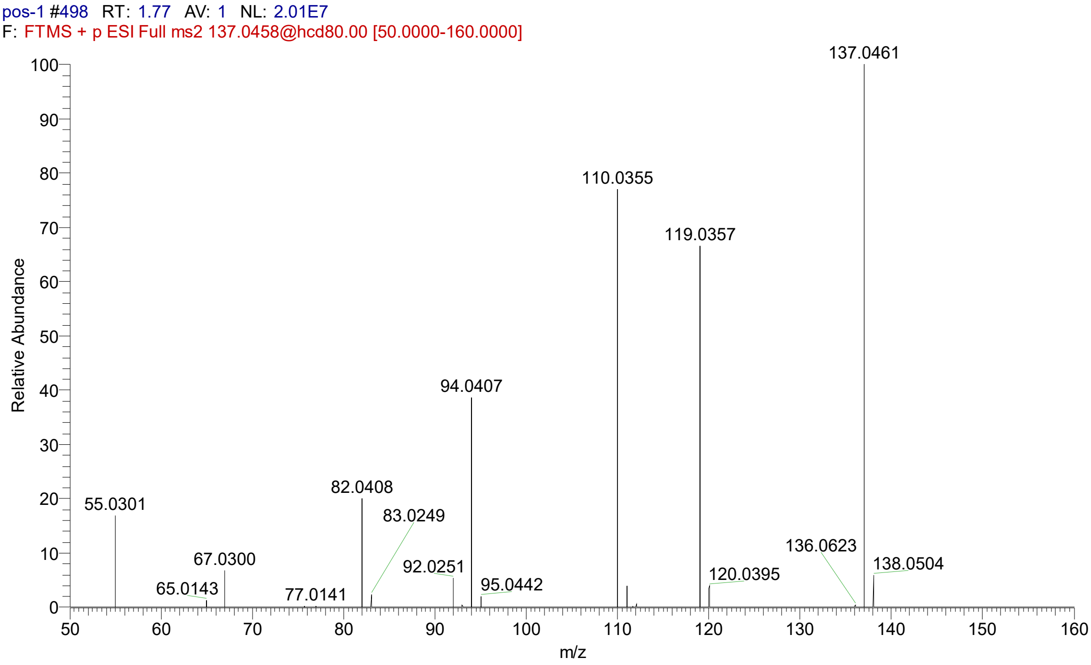

Figure S11. The MS/MS spectrum at *m/z* 137.0463 (Table S10) identifies it as hypoxanthine when compared with the standard given in the standard hypoxanthine (below).

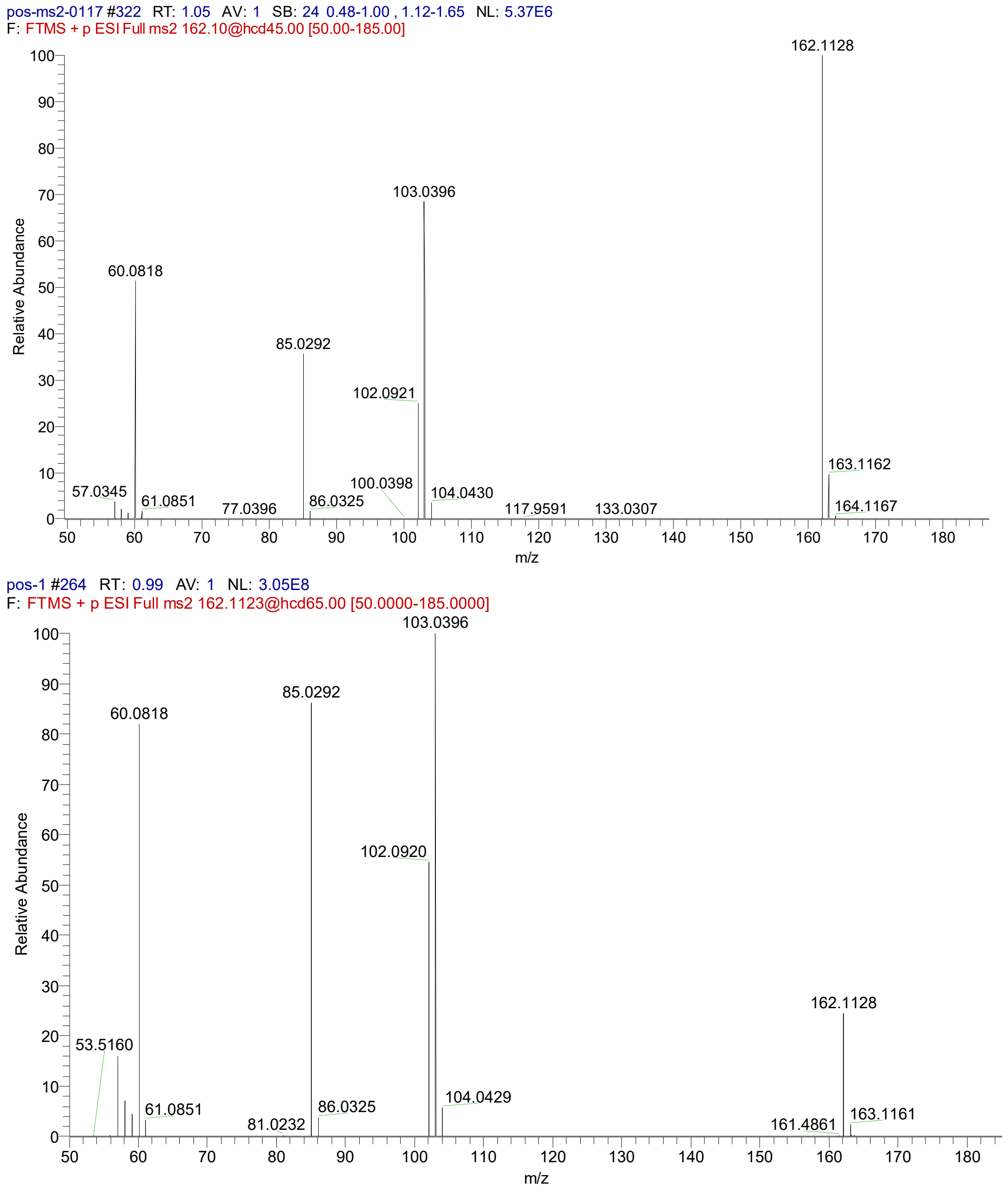

Figure S12. The MS/MS spectrum at *m/z* 162.1128 (Table S10) identifies it as carnitine when compared with the standard given in the standard carnitine (below).

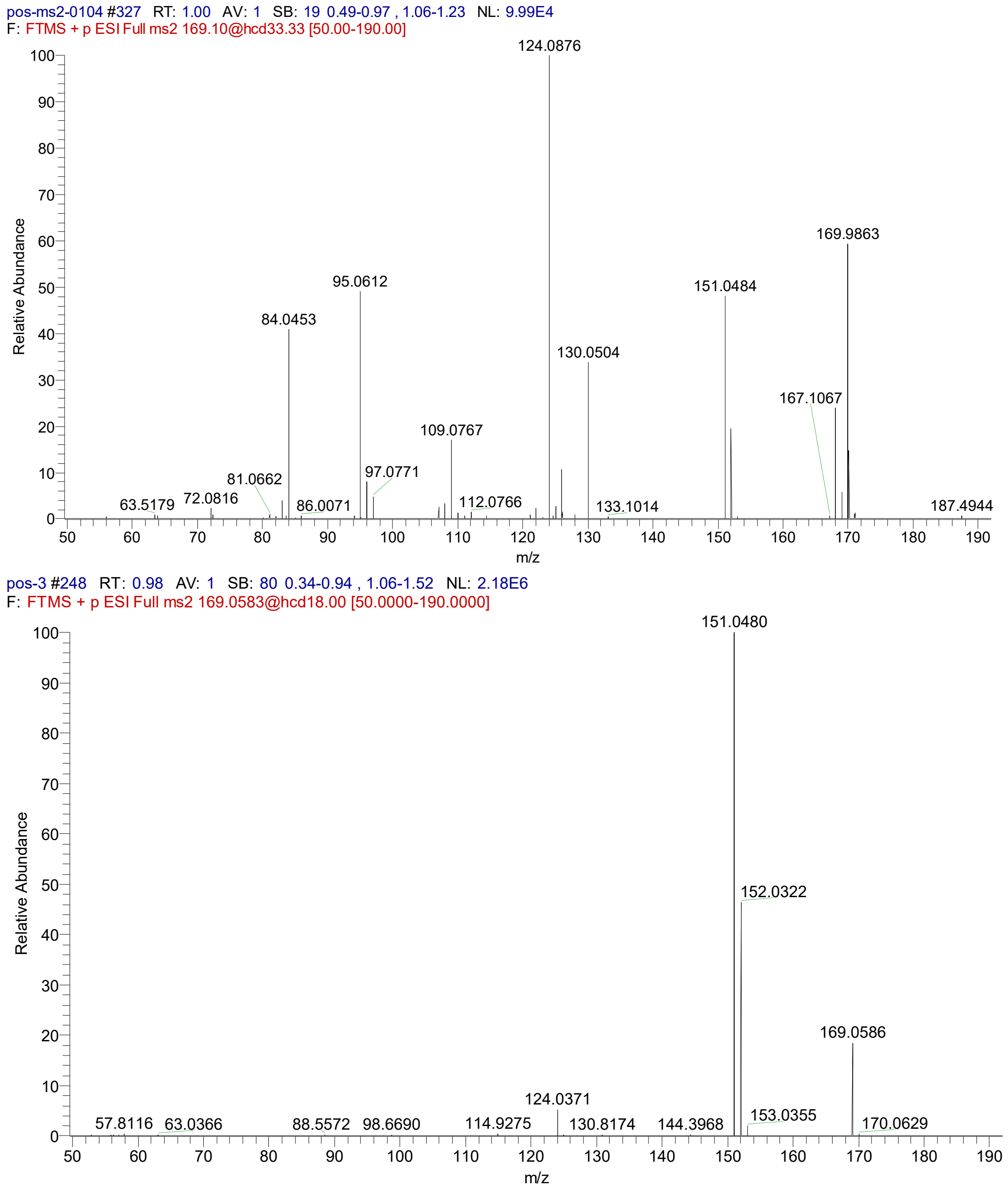

Figure S13. The MS/MS spectrum at *m/z* 169.0583 (Table S10) identifies it as glutamine when compared with the standard given in the standard glutamine (below).

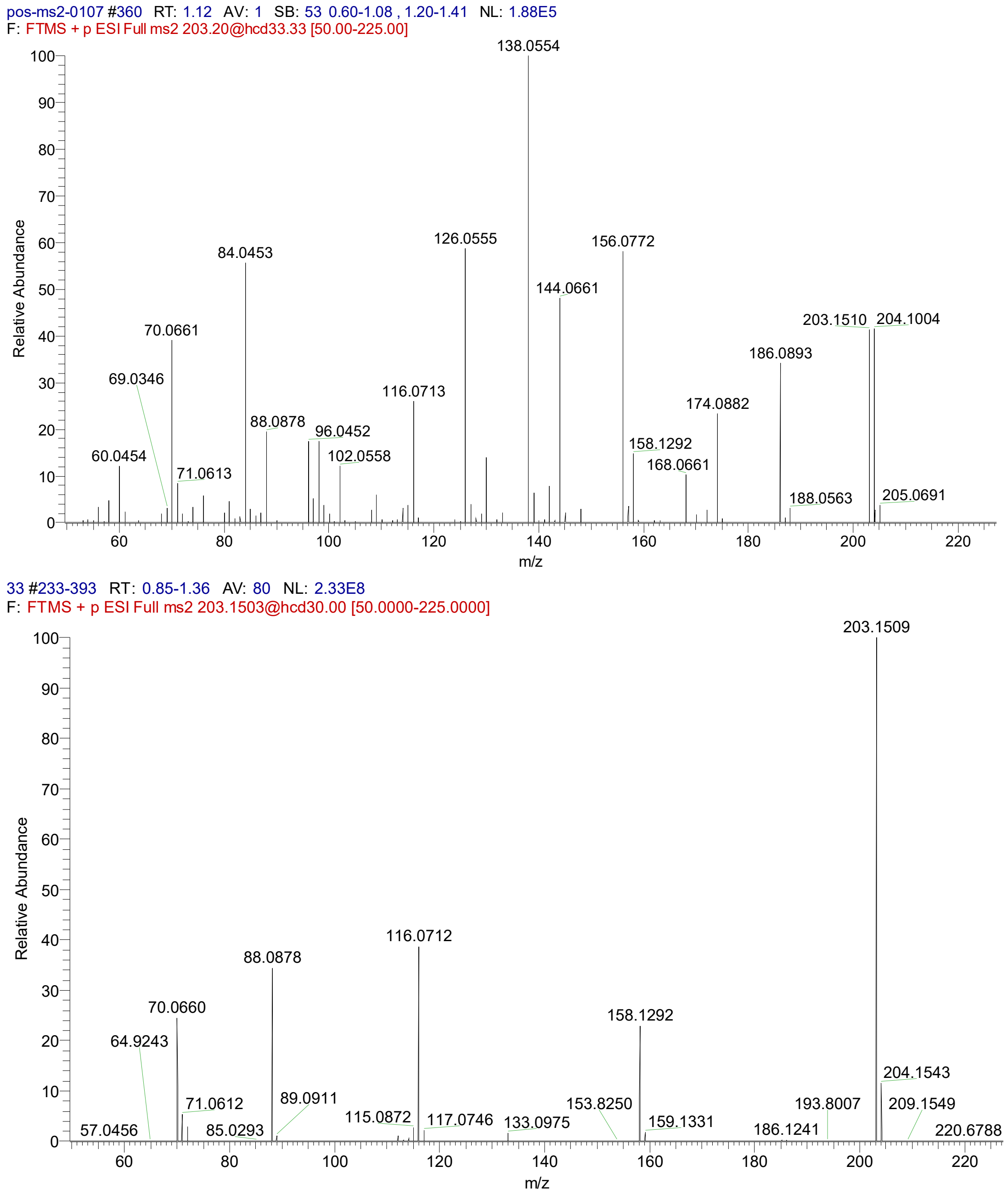

Figure S14. The MS/MS spectrum at *m/z* 203.1510 (Table S10) identifies it as dimethylarginine when compared with the standard given in the standard dimethylarginine (below).

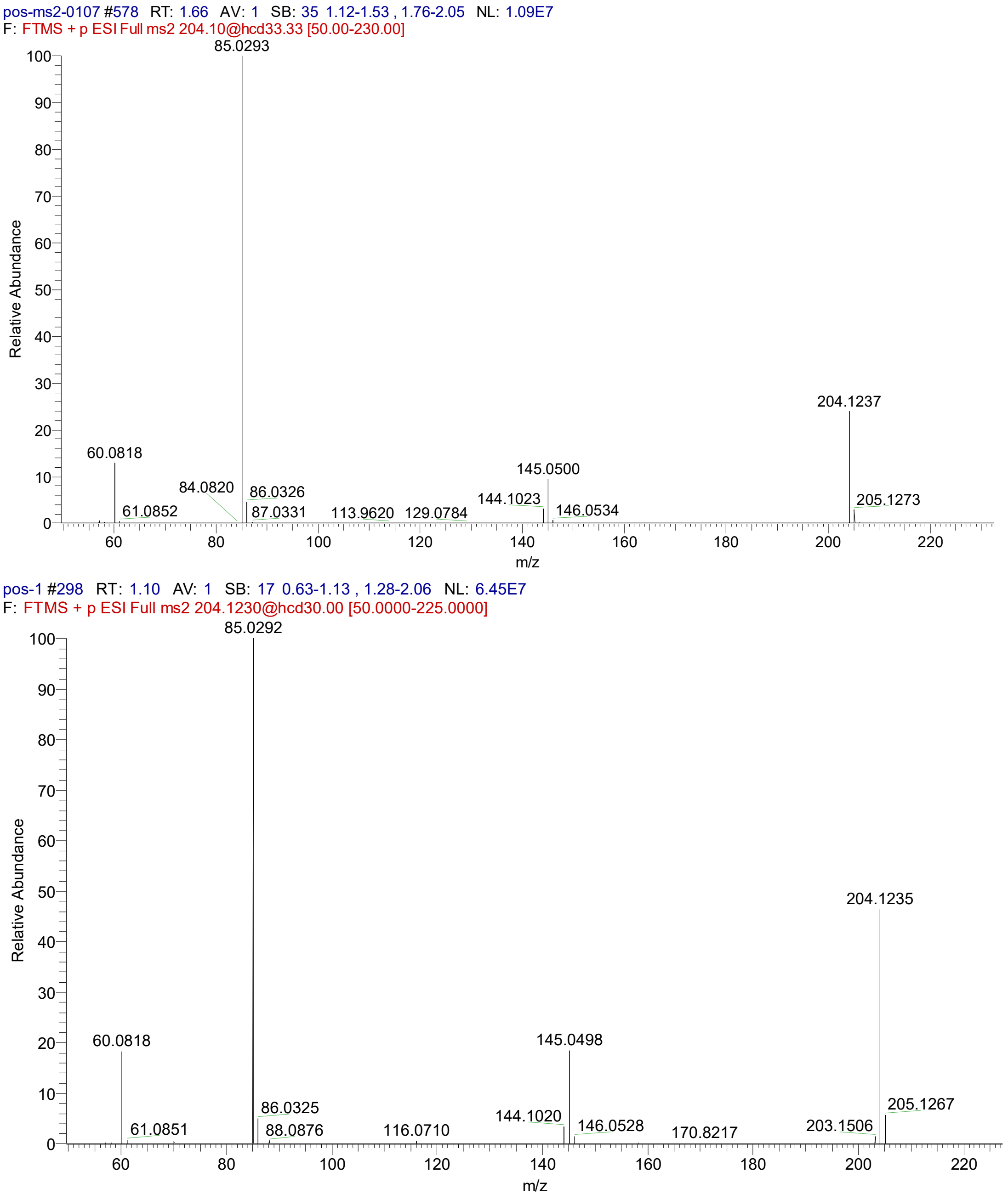

Figure S15. The MS/MS spectrum at *m/z* 204.1237 (Table S10) identifies it as acetylcarnitine when compared with the standard given in the standard acetylcarnitine (below).

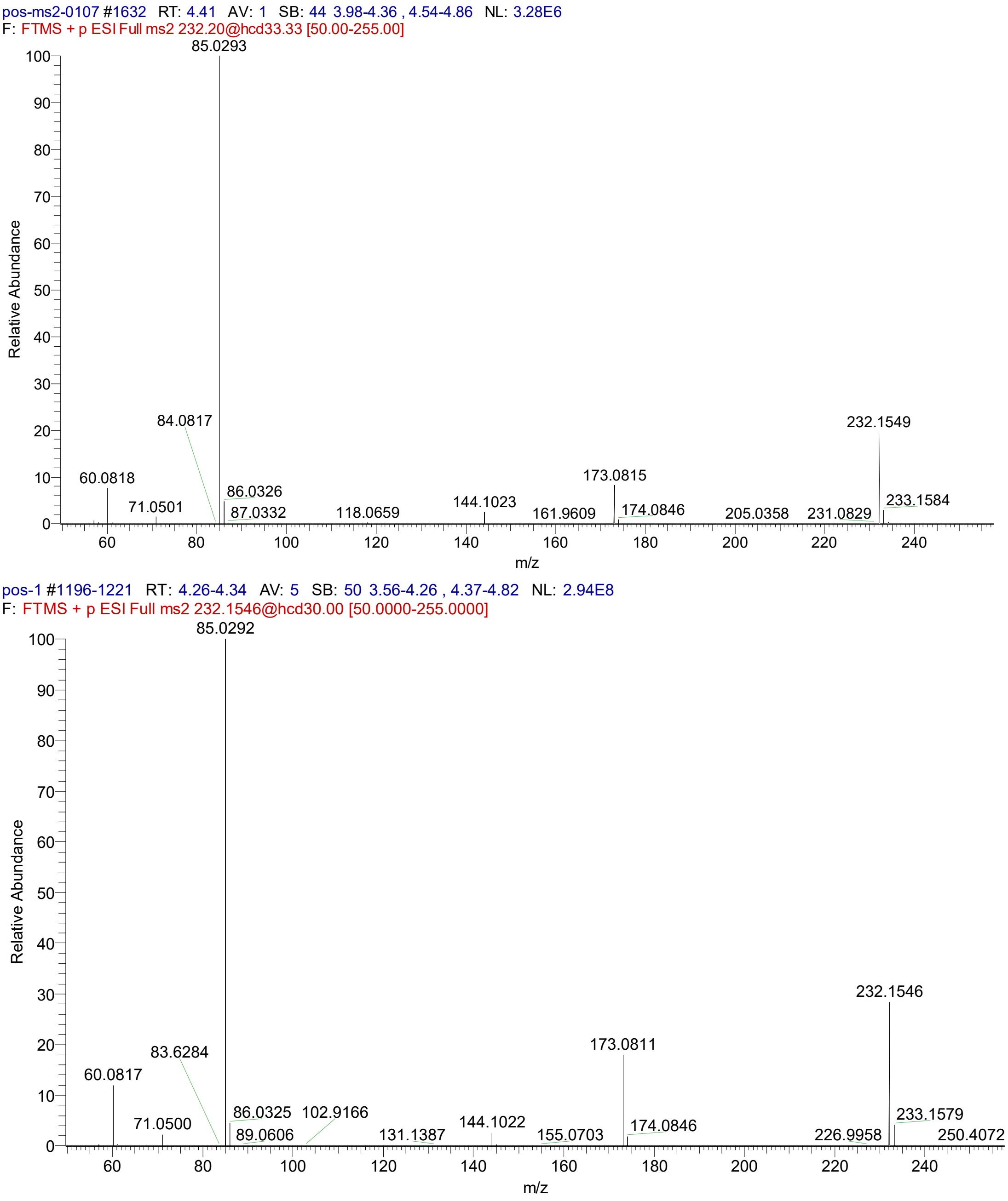

Figure S16. The MS/MS spectrum at *m/z* 232.1549 (Table S10) identifies it as butyryl-L-carnitine when compared with the standard given in the standard butyryl-L-carnitine (below).

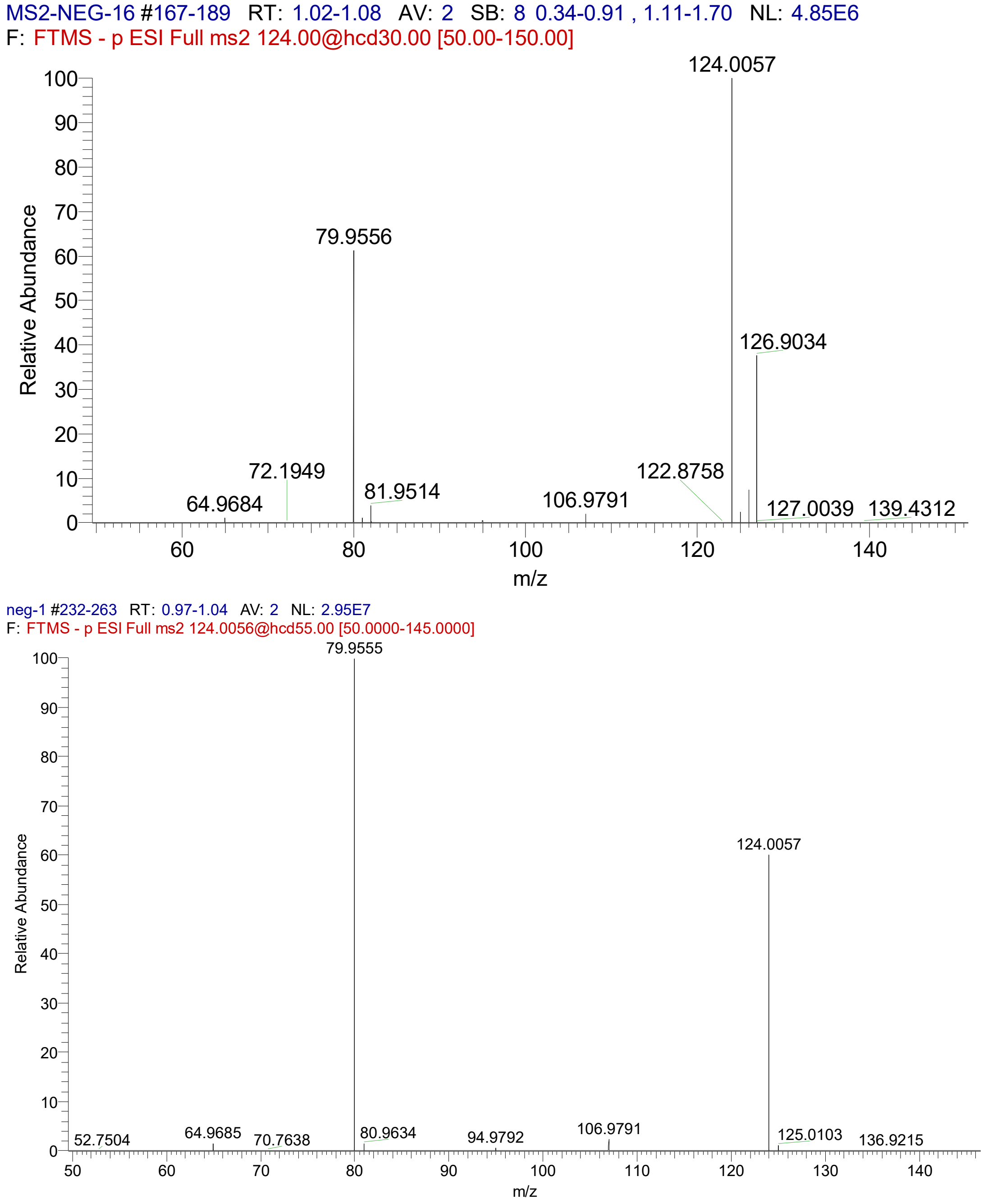

Figure S17. The MS/MS spectrum at *m/z* 124.0057 (Table S10) identifies it as taurine when compared with the standard given in the standard taurine (below).

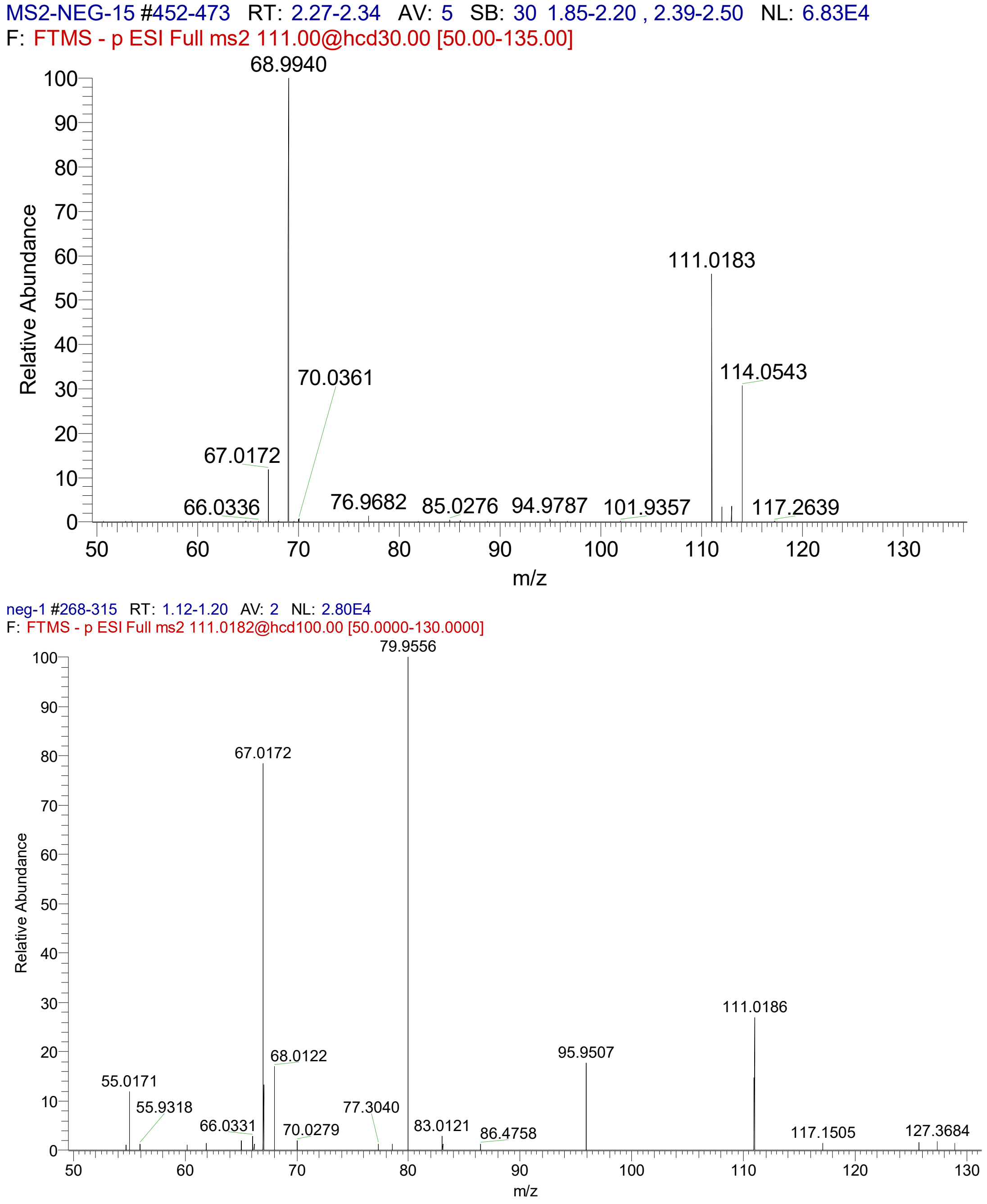

Figure S18. The MS/MS spectrum at *m/z* 111.0183 (Table S10) identifies it as uracil when compared with the standard given in the standard uracil (below).

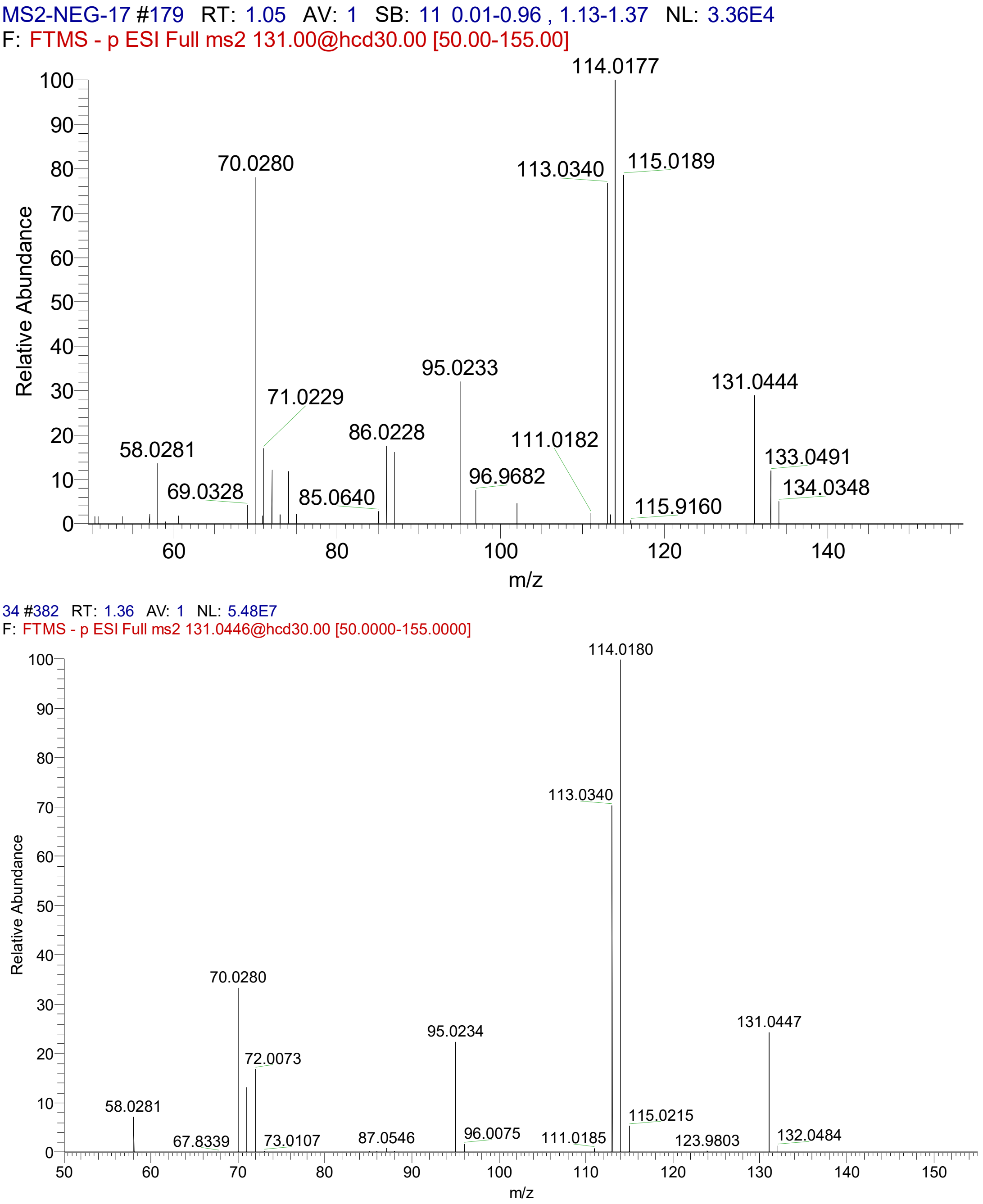

Figure S19. The MS/MS spectrum at *m/z* 131.0444 (Table S10) identifies it as asparagine when compared with the standard given in the standard asparagine (below).

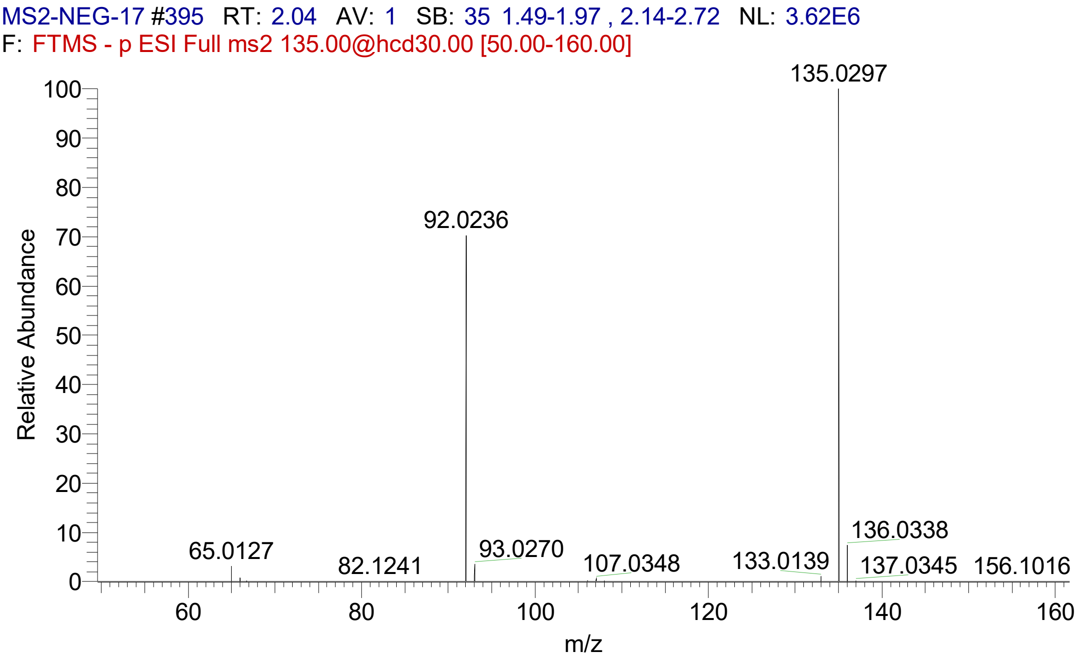

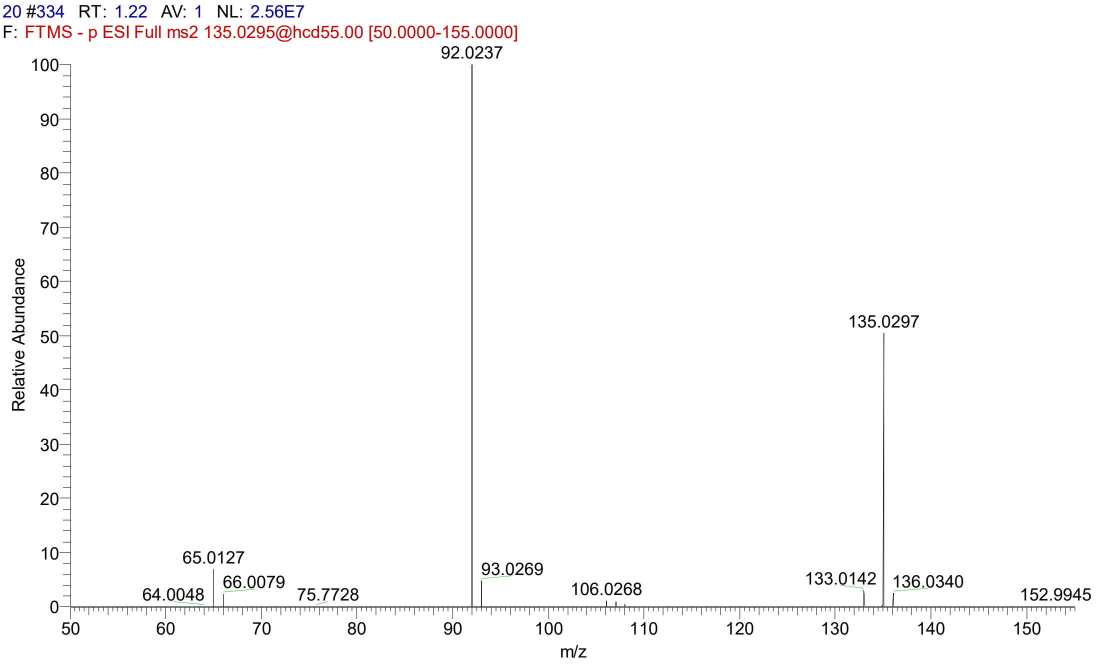

Figure S20. The MS/MS spectrum at *m/z* 135.0297 (Table S10) identifies it as hypoxanthine when compared with the standard given in the standard hypoxanthine (below).

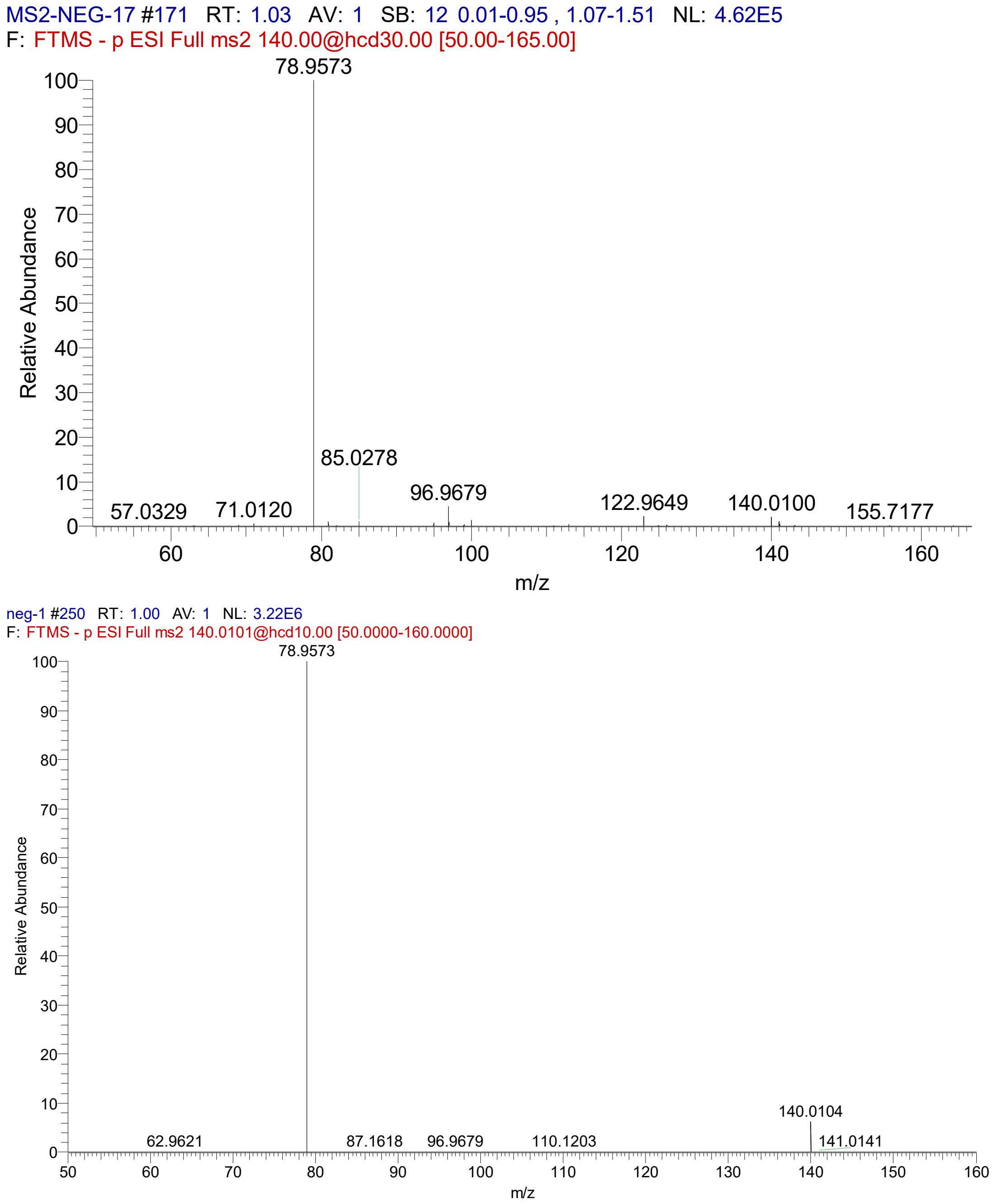

Figure S21. The MS/MS spectrum at *m/z* 140.0100 (Table S10) identifies it as O-phosphorylethanolamine when compared with the standard given in the standard O-phosphorylethanolamine (below).

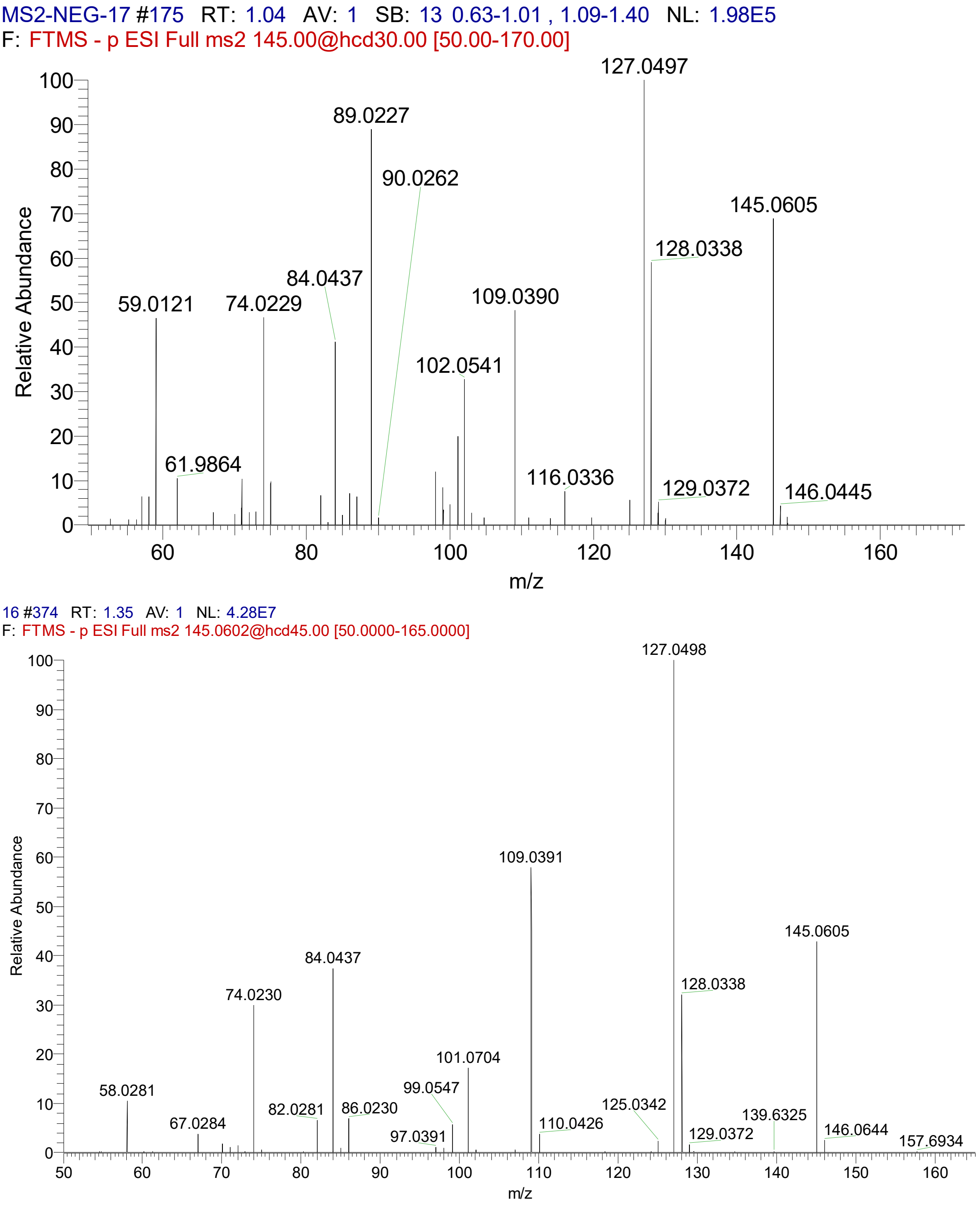

Figure S22. The MS/MS spectrum at *m/z* 145.0605 (Table S10) identifies it as glutamine when compared with the standard given in the standard glutamine (below).

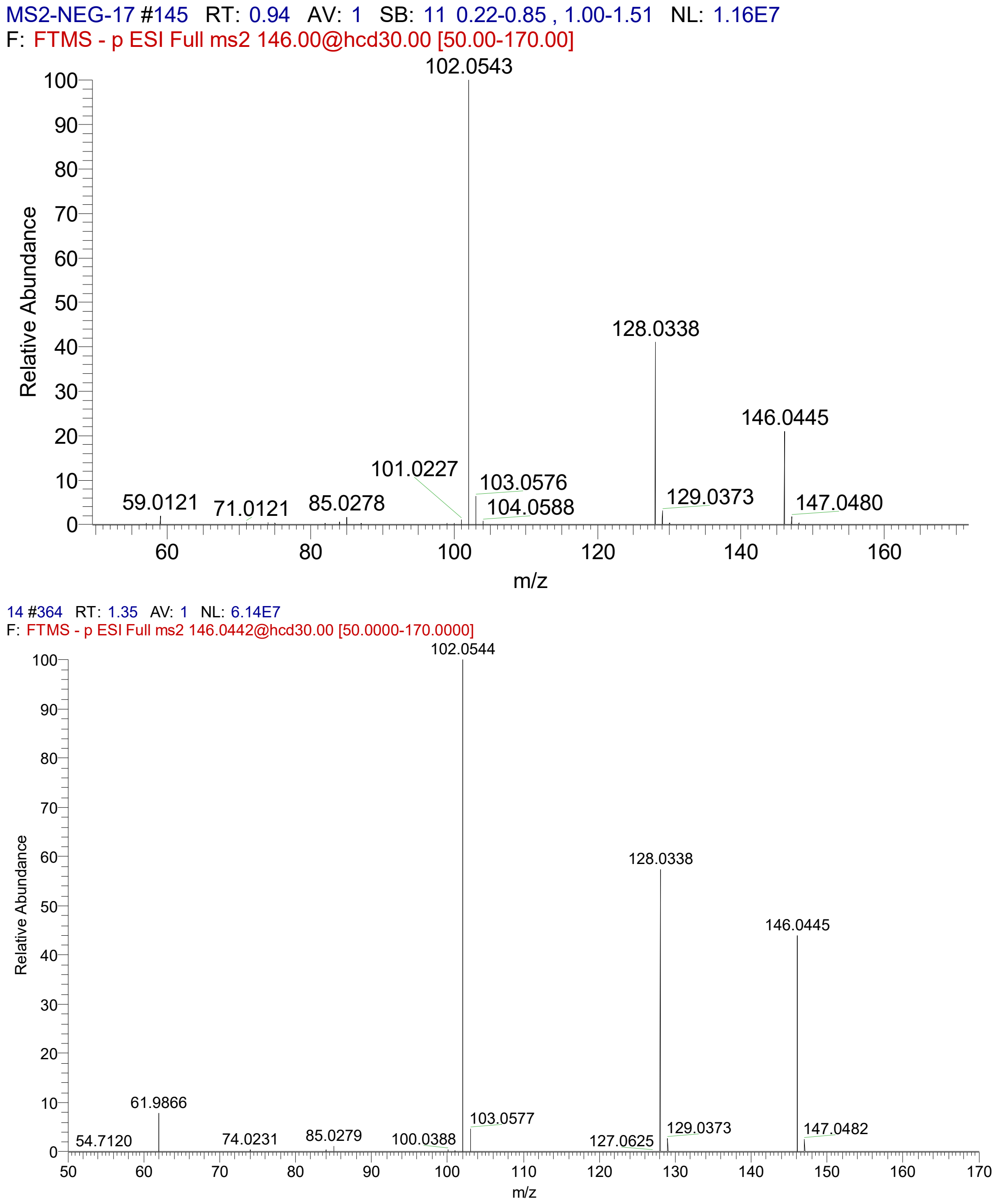

Figure S23 The MS/MS spectrum at *m/z* 146.0445 (Table S10) identifies it as glutamate when compared with the standard given in the standard glutamate (below).

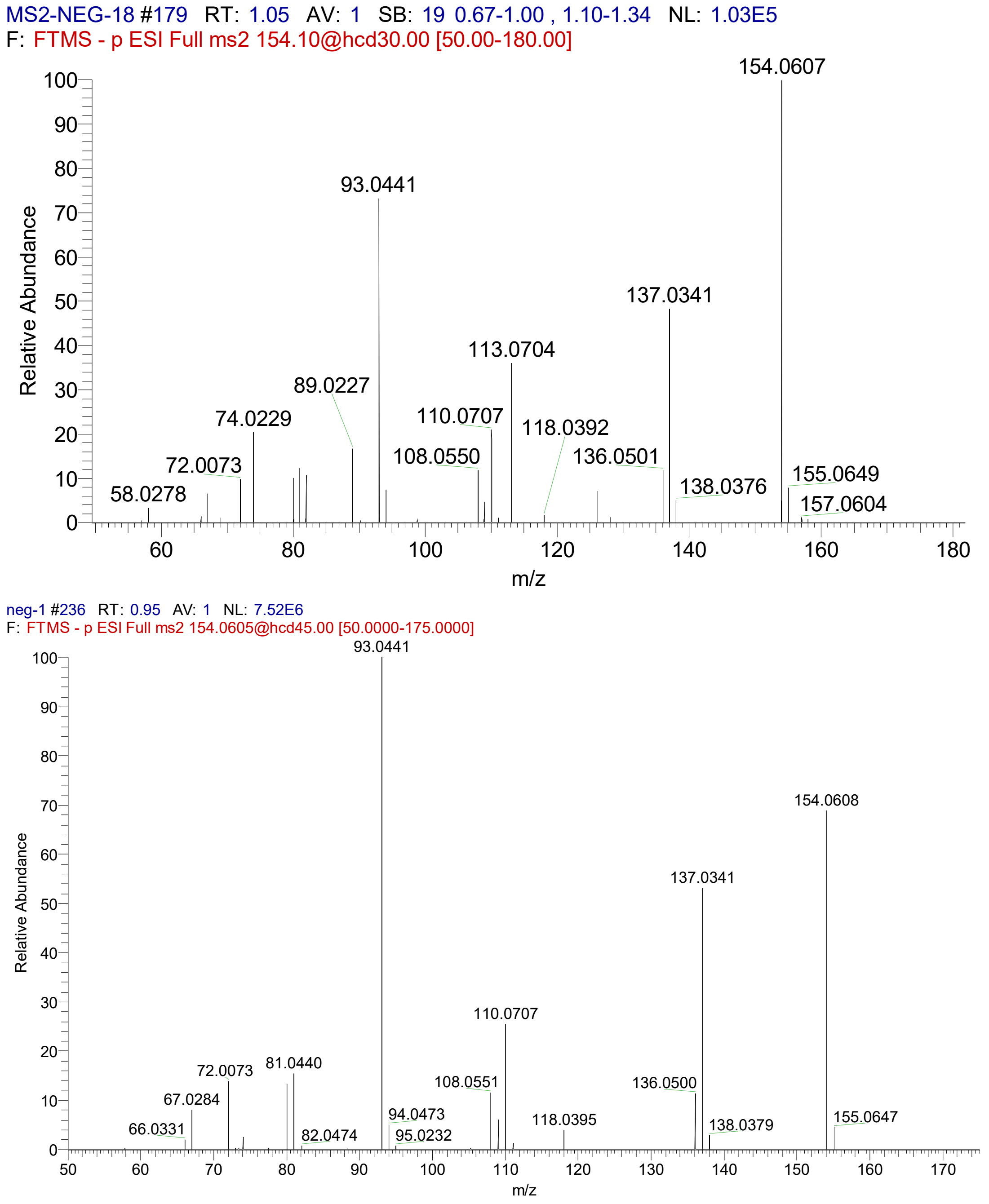

Figure S24. The MS/MS spectrum at *m/z* 154.0607 (Table S10) identifies it as histidine when compared with the standard given in the standard histidine (below).

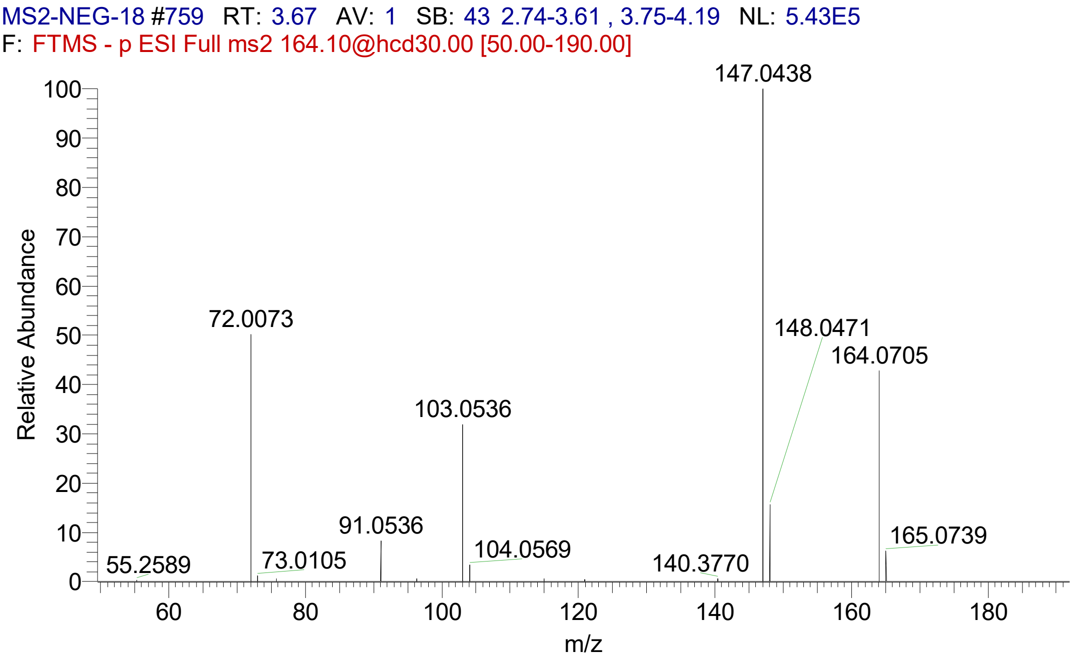

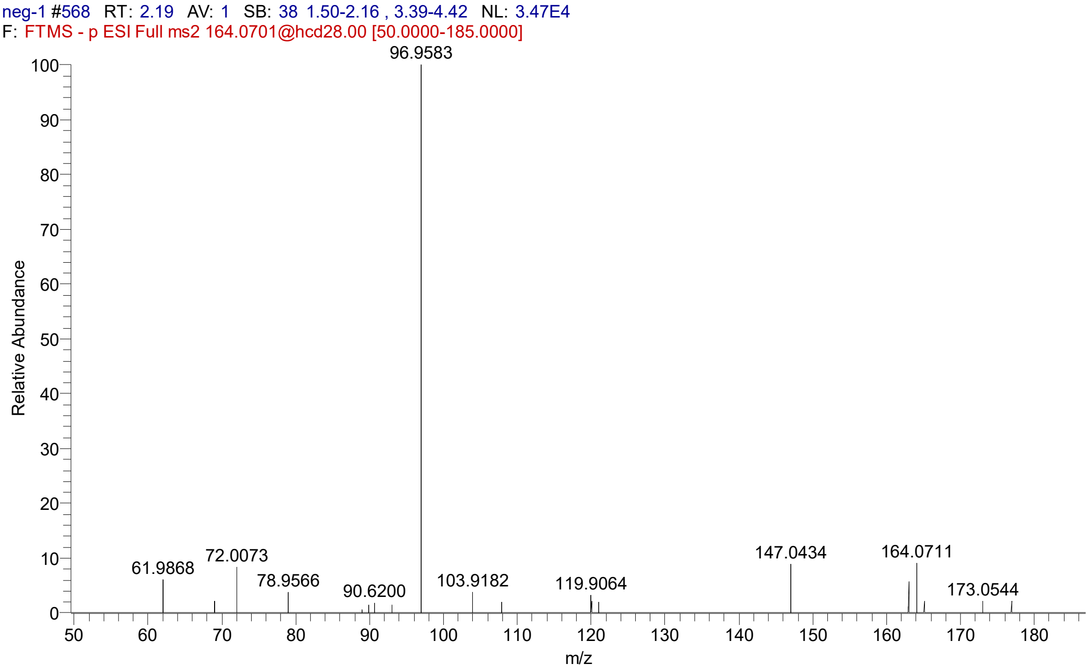

Figure S25. The MS/MS spectrum at *m/z* 164.0705 (Table S10) identifies it as phenylalanine when compared with the standard given in the standard phenylalanine (below).

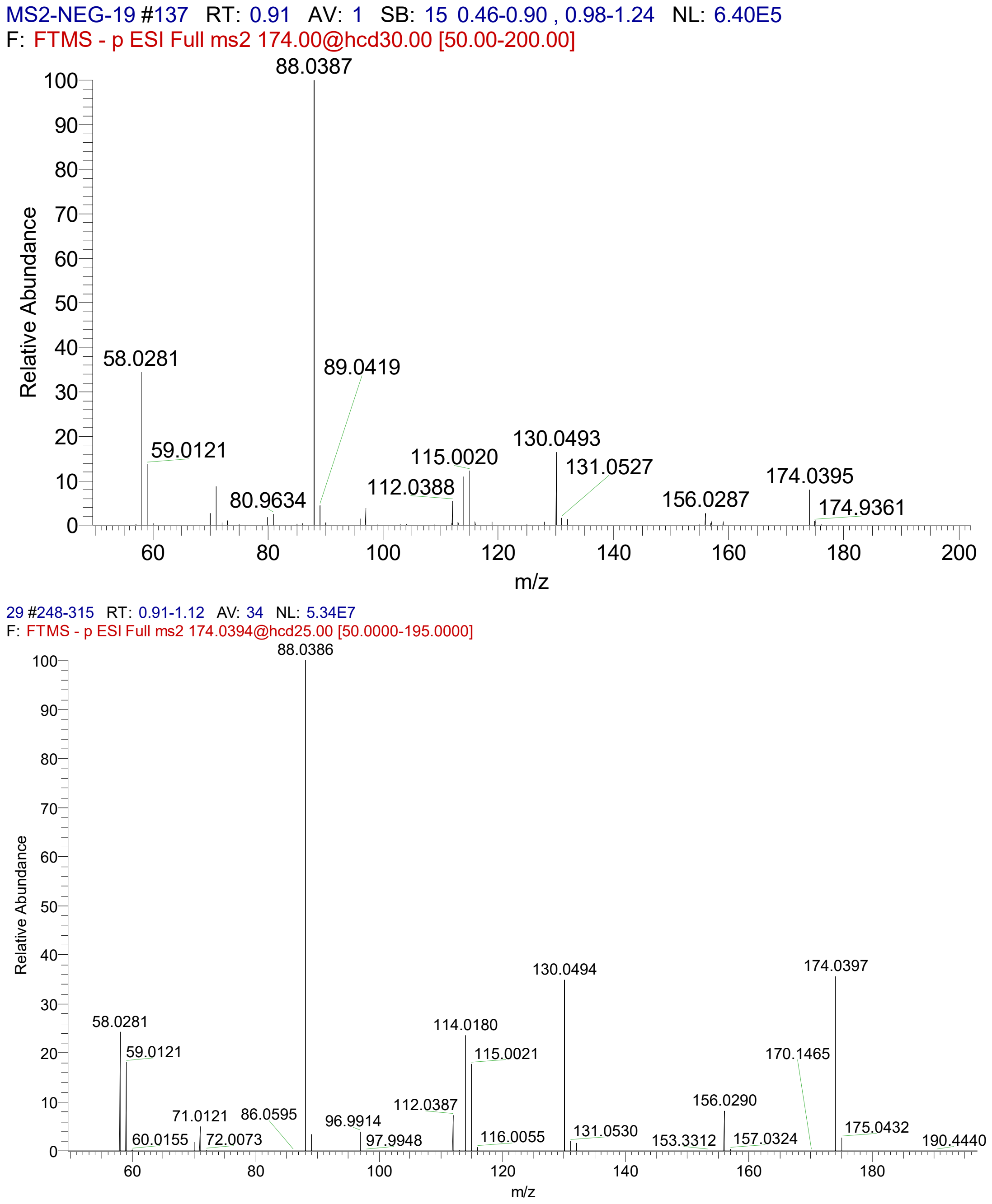

Figure S26. The MS/MS spectrum at *m/z* 174.0395 (Table S10) identifies it as N-acetylaspartate when compared with the standard given in the standard N-acetylaspartate (below).

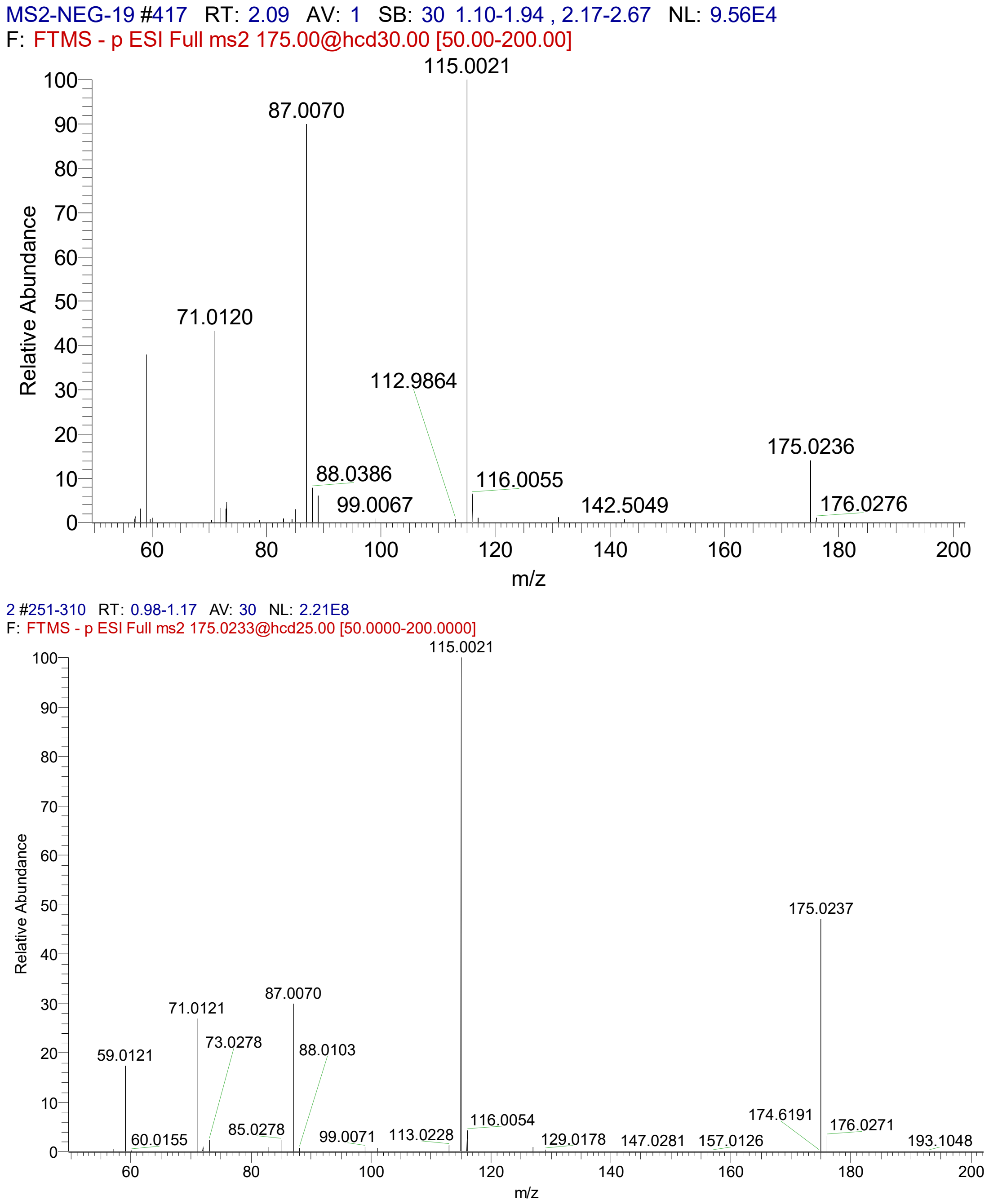

Figure S27. The MS/MS spectrum at *m/z* 175.0236 (Table S10) identifies it as ascorbic acid when compared with the standard given in the standard ascorbic acid (below).

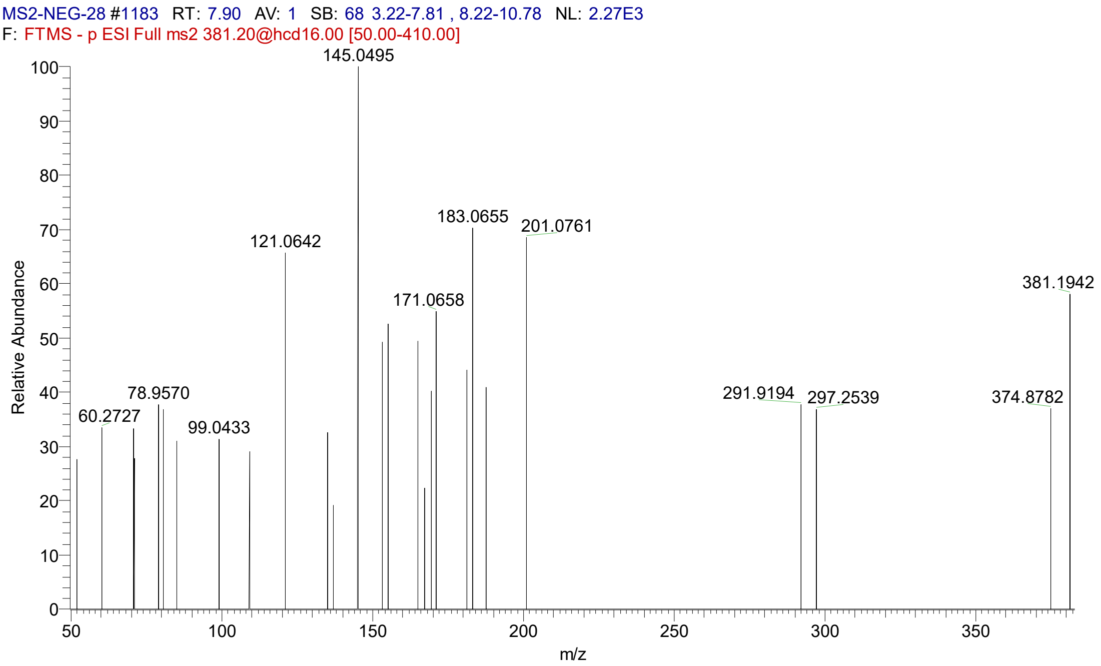

Figure S28. The MS/MS spectrum at *m/z* 381.1942 (Table S10) identifies it as 12-Oxo-20-trihydroxy-leukotriene B4 when compared with the standard given in the online metabolite database.

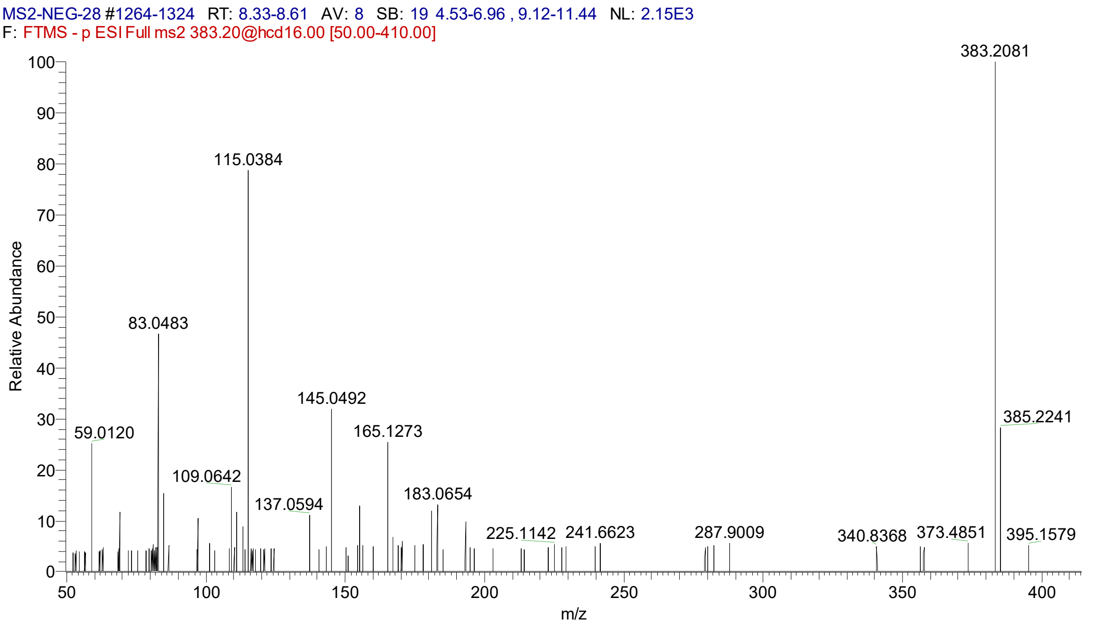

Figure S29. The MS/MS spectrum at *m/z* 383.2081 (Table S10) identifies it as 20-Trihydroxy-leukotriene-B4 when compared with the standard given in the online metabolite database.

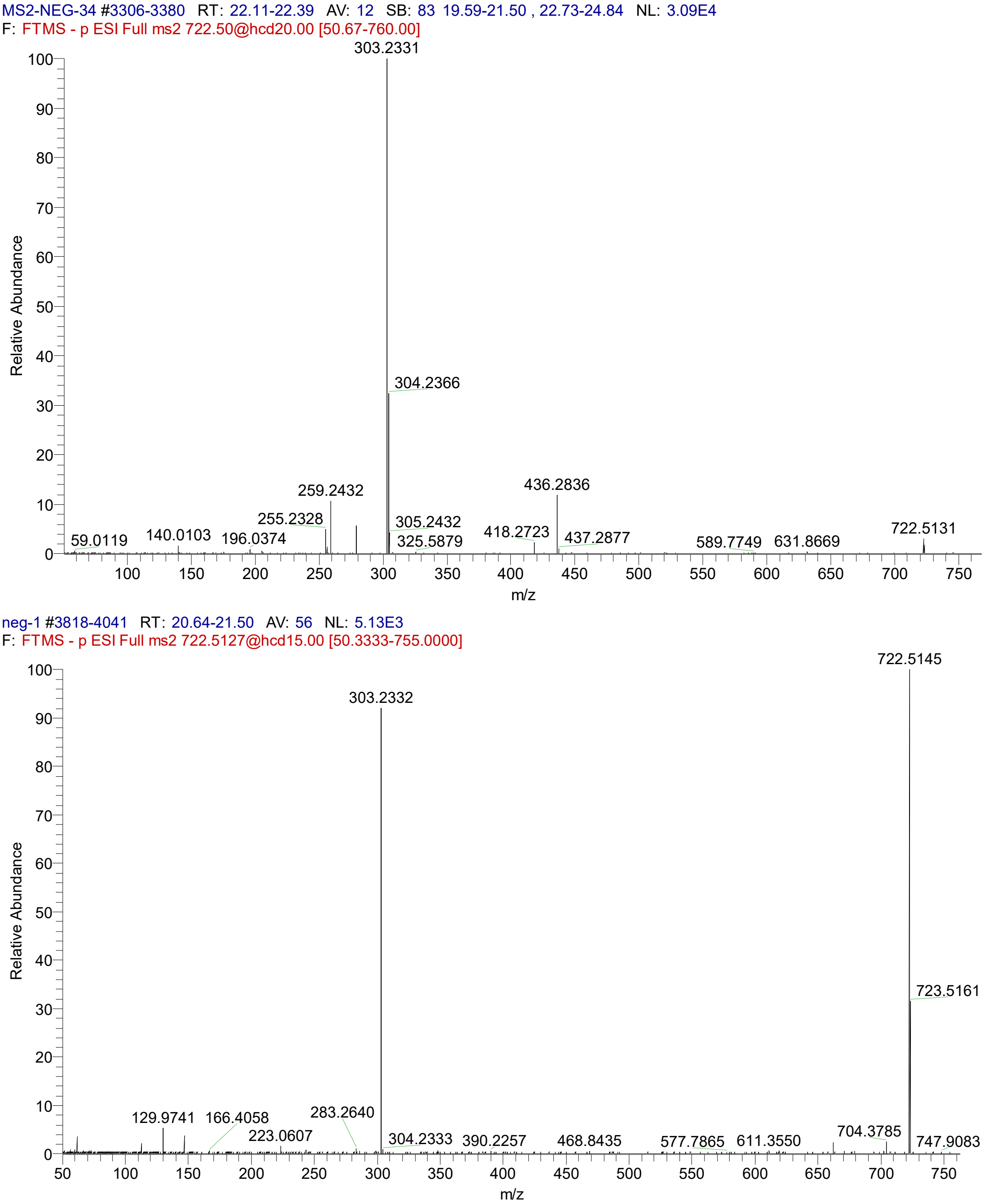

Figure S30. The MS/MS spectrum at *m/z* 722.5131 (Table S10) identifies it as PE(36:4) when compared with the standard given in the standard PE(36:4) (below).

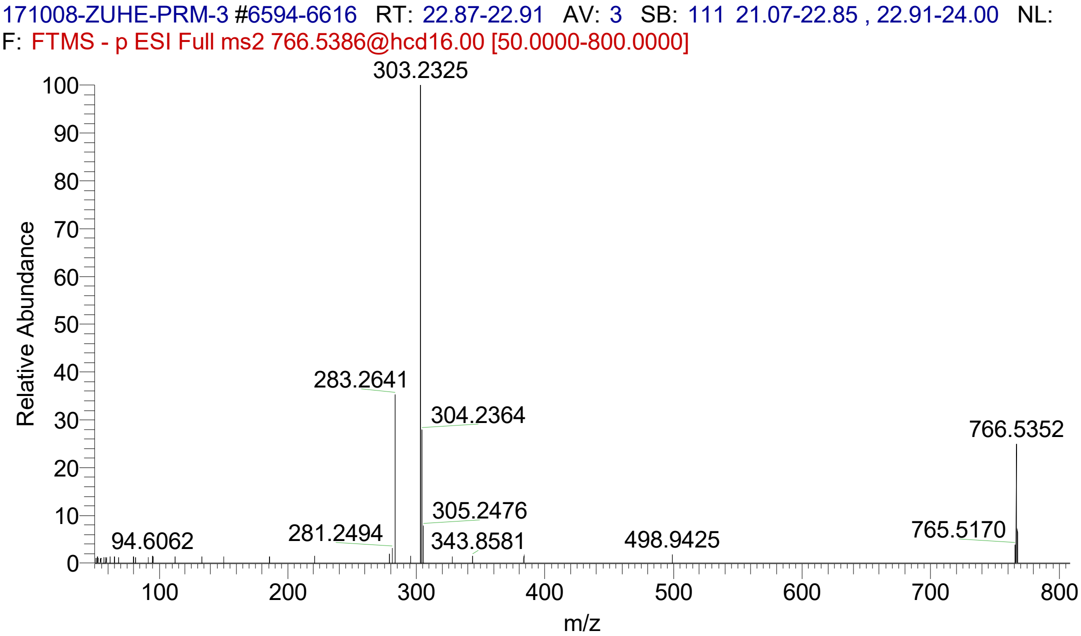

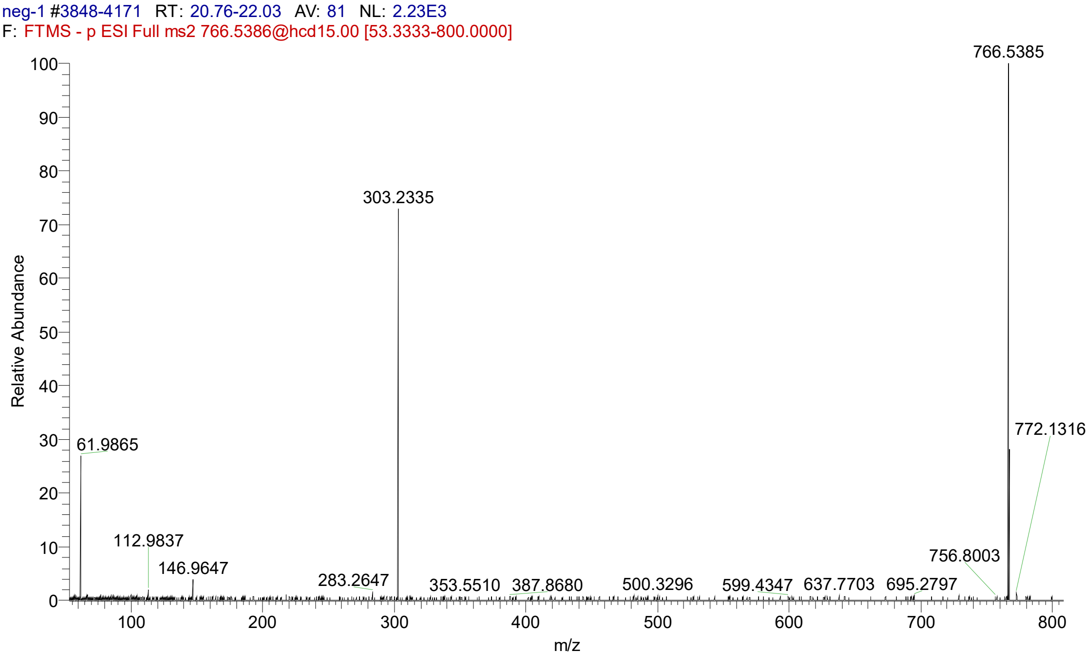

Figure S31. The MS/MS spectrum at *m/z* 766.5352 (Table S10) identifies it as PE(38:4) when compared with the standard given in the standard PE(38:4) (below).

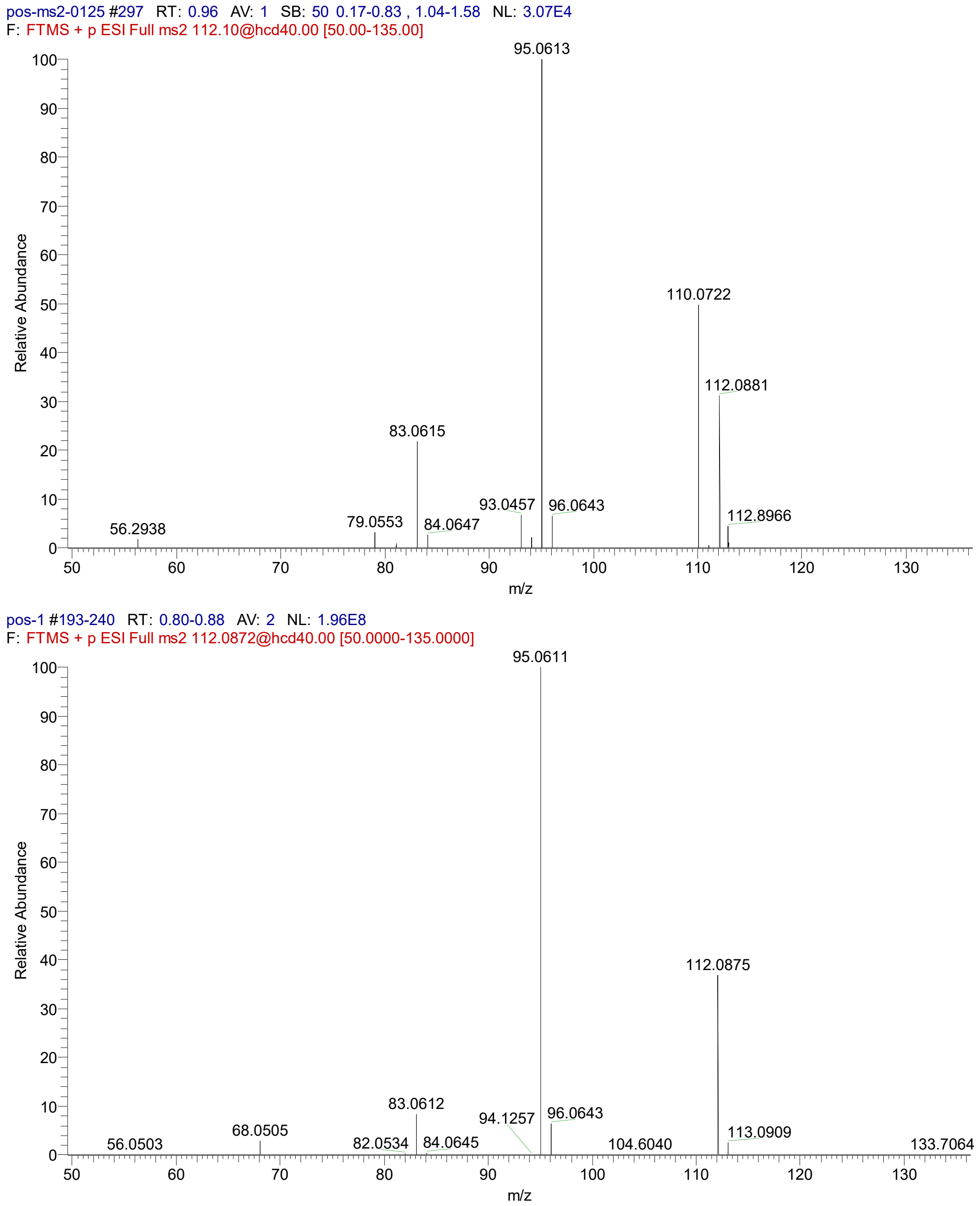

Figure S32. The MS/MS spectrum at *m/z* 112.0881 (Table S13) identifies it as histamine when compared with the standard given in the standard histamine (below).

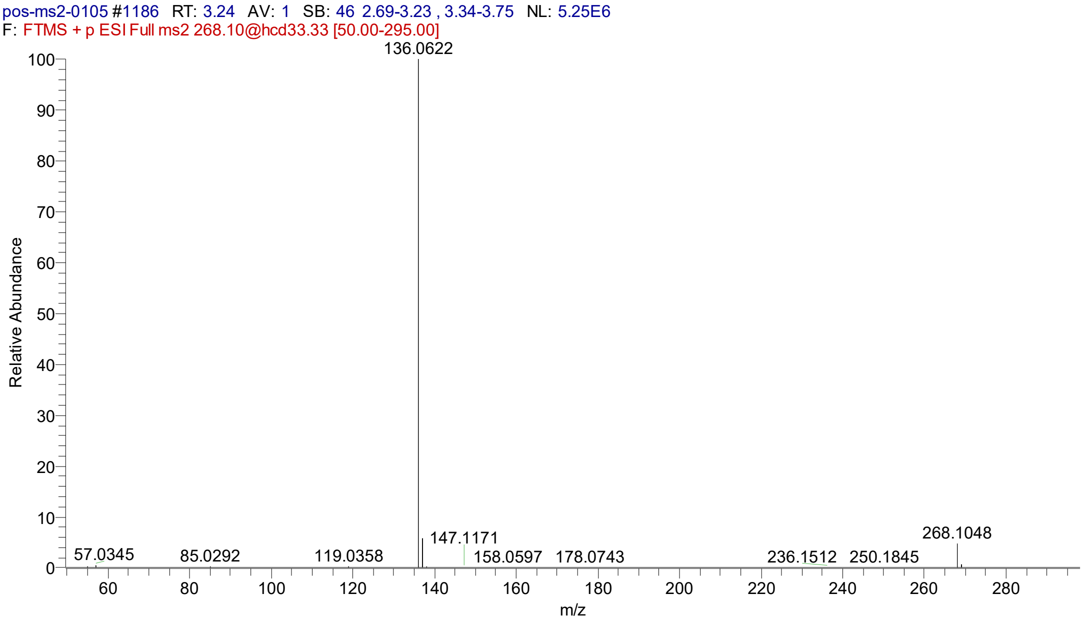

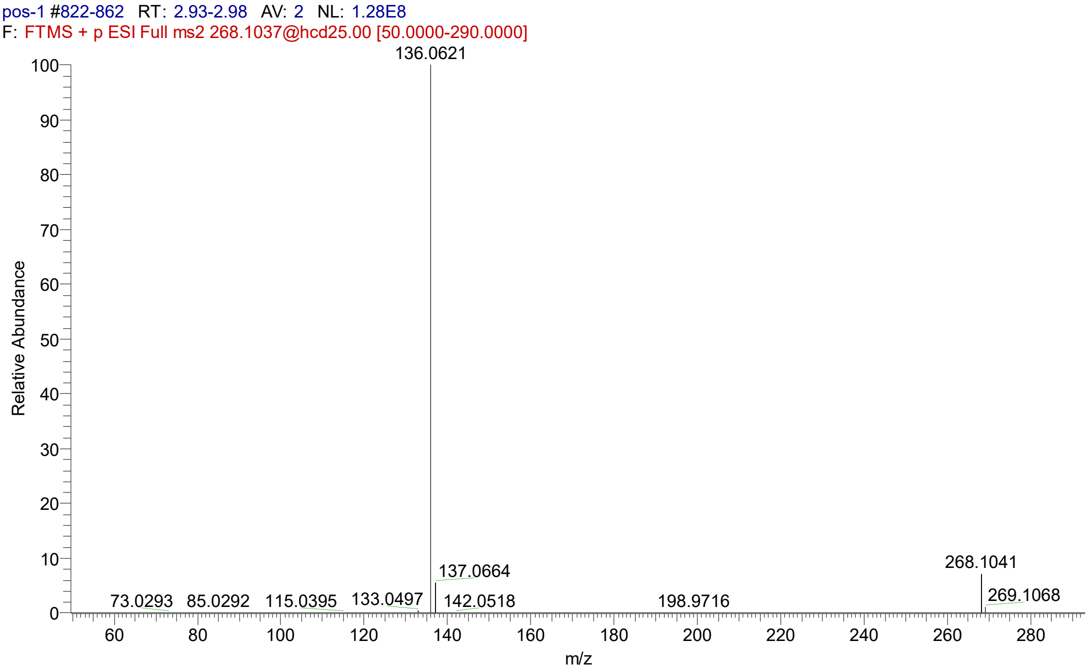

Figure S33. The MS/MS spectrum at *m/z* 268.1048 (Table S13) identifies it as adenosine when compared with the standard given in the standard adenosine (below).

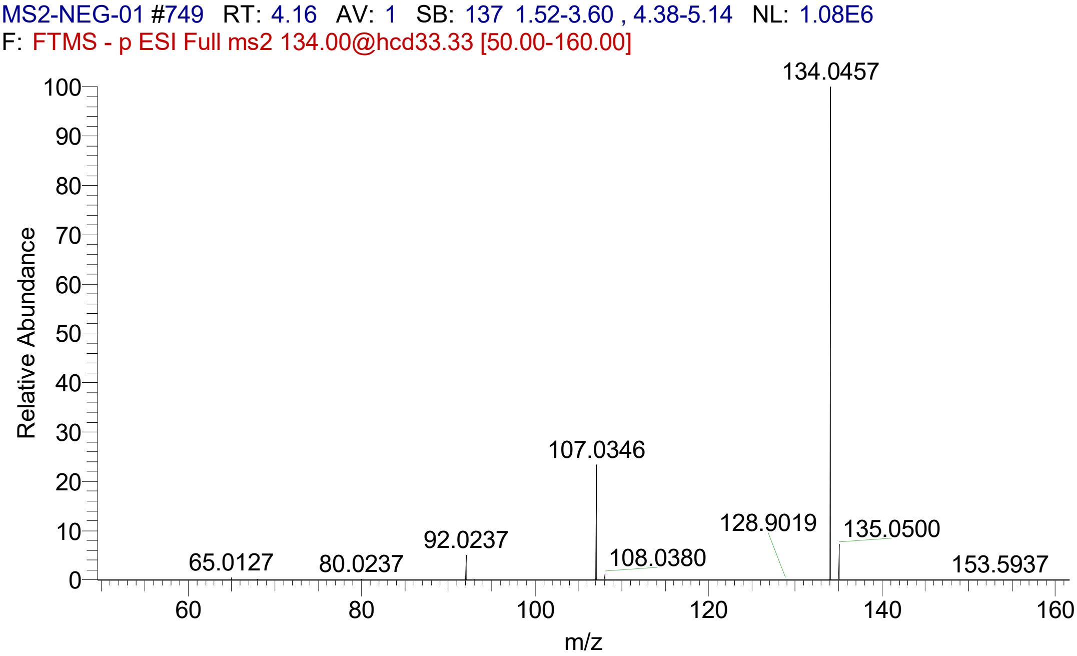

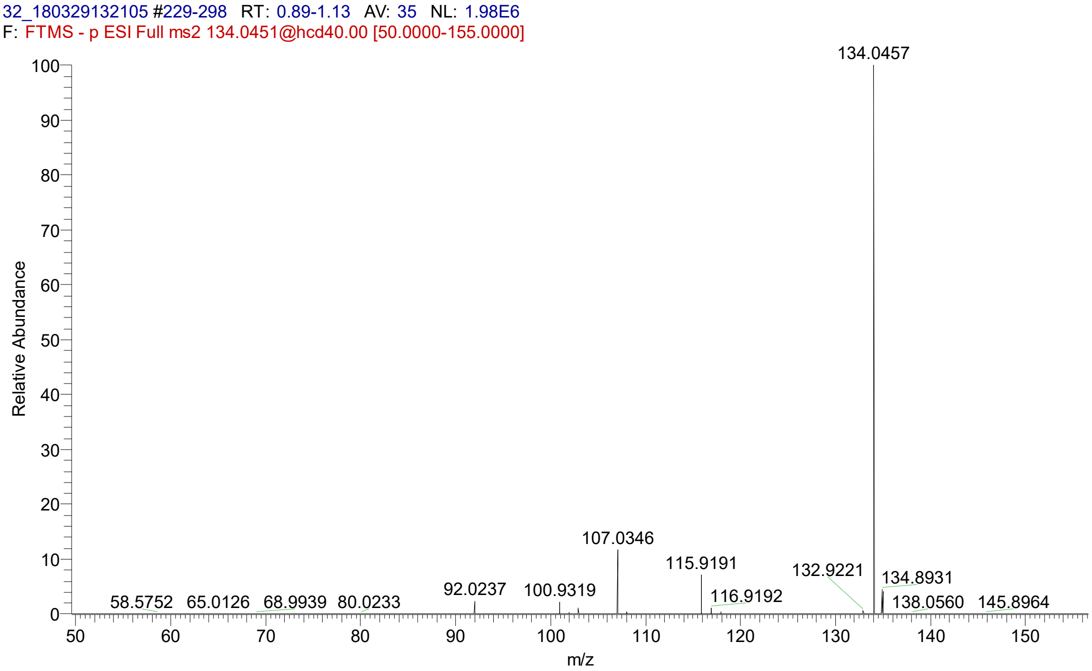

Figure S34. The MS/MS spectrum at *m/z* 134.0457 (Table S13) identifies it as adenine when compared with the standard given in the standard adenine (below).

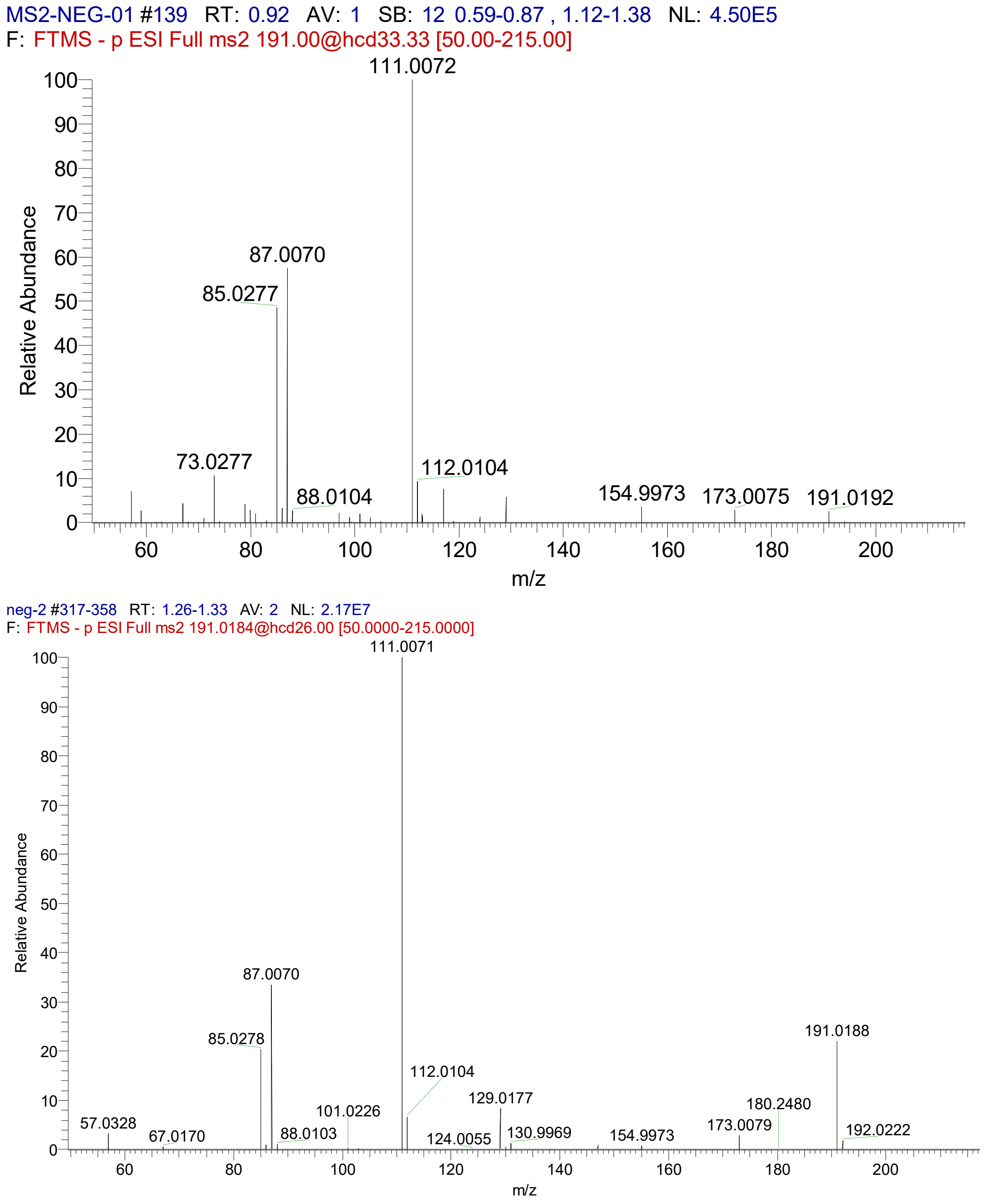

Figure S35. The MS/MS spectrum at *m/z* 191.0192 (Table S13) identifies it as citric acid when compared with the standard given in the standard citric acid (below).

SI References

1. A. Wojakowska, M. Chekan, P. Widlak, M. Pietrowska, Application of metabolomics in thyroid cancer research. *Int. J. Endocrinol* 2015, 258763-258763 (2015).

2. S. Ishikawa et al., Increased Expression of Phosphatidylcholine (16:0/18:1) and (16:0/18:2) in Thyroid Papillary Cancer. *PLoS One* 7, e48873 (2012).

3. M. Nipp et al., S100-A10, thioredoxin, and S100-A6 as biomarkers of papillary thyroid carcinoma with lymph node metastasis identified by MALDI imaging. *J. Mol. Med* 90, 163-174 (2012).

4. S. Guo et al., Tissue imaging and serum lipidomic profiling for screening potential biomarkers of thyroid tumors by matrix-assisted laser desorption/ionization-Fourier transform ion cyclotron resonance mass spectrometry. *Anal. Bioanal. Chem* 406, 4357-4370 (2014).

5. K.-W. Min et al., Imaging mass spectrometry in papillary thyroid carcinoma for the identification and validation of biomarker proteins. *J. Korean Med. Sci* 29, 934-940 (2014).

6. F. Pagni et al., Proteomics in thyroid cytopathology: Relevance of MALDI-imaging in distinguishing malignant from benign lesions. *Proteomics* 16, 1775-1784 (2016).

7. M. Pietrowska et al., Molecular profiles of thyroid cancer subtypes: Classification based on features of tissue revealed by mass spectrometry imaging. *Biochim. Biophys. Acta* 1865, 837-845 (2017).

8. M. Galli et al., Proteomic profiles of thyroid tumors by mass spectrometry-imaging on tissue microarrays. *Biochim. Biophys. Acta* 1865, 817-827 (2017).

9. J. Zhang et al., Cardiolipins Are Biomarkers of Mitochondria-Rich Thyroid Oncocytic Tumors. *Cancer Res* 76, 6588-6597 (2016).

10. J. Zhang et al., Detection of Metastatic Breast and Thyroid Cancer in Lymph Nodes by Desorption Electrospray Ionization Mass Spectrometry Imaging. *J. Am. Soc. Mass Spectrom* 28, 1166-1174 (2017).

11. R. J. DeHoog et al., Preoperative metabolic classification of thyroid nodules using mass spectrometry imaging of fine-needle aspiration biopsies. *P.Natl. Acad. Sci. USA* 116, 21401-21408 (2019).
